# Supplementary material for: Three chromosome-scale Papaver genomes reveal punctuated patchwork evolution of the morphinan and noscapine biosynthesis pathway
Source: Nat Commun. 2021 Oct 15;12:6030. doi: 10.1038/s41467-021-26330-8 (PMC8521590; doi:10.1038/s41467-021-26330-8)
Supplement: Supplementary file 1 — Supplementary Information [file 41467_2021_26330_MOESM1_ESM.pdf]

Three chromosome-scale *Papaver* genomes reveal punctuated patchwork evolution of the morphinan and noscapine biosynthesis pathway

Yang *et al*.

Supplementary Method 1. Plant materials and growth

For sequencing and assembly of the *Papaver setigerum, P. rhoeas* and *P. somniferum* genomes and transcriptomes, *P. setigerum* variety DCW1, *P. rhoeas* variety YMR1 and *P. somniferum* variety HN1 were grown in Azalea pots in a regulated growth chamber with 16 hours of light located at Xi'an Jiaotong University Laboratory of BioData Sciences. The growth substrate is a soil mix of four parts potting mix, two parts natural soil and one-part Vermiculite. For long-read genome sequencing and chromatin conformation capture (Hi-C) sequencing, fresh leaves (the four uppermost ones) were harvested from six weeks old seedlings of three species. For transcriptome sequencing, material was sampled from the following six tissue types on the first day of anthesis: root, leaves, stem (the 2 cm long part just underneath the capsule), capsule, petals, and stamens. All materials for sequencing in this study were collected, rinsed with water and surface-sterilized with 70% ethanol for 10 minutes to remove commensal contaminants before being processed for library construction and sequencing.

Supplementary Method 2. Quantification of morphinans using HPLC-MS

The content of morphine, codeine and thebaine was determined using HPLC-MS/MS method. The system consists of two LC-20ADXR pumps, a CBM-20A communication bus module, an LCMS-8040 triple quadruple mass spectrometer, and a Lab Solutions work station (Shimadzu Corporation, Kyoto, Japan). A HPLC column (VP-ODS, 150 mm × 2.0 mm I.D., 5 µm, Shimadzu Corporation) was used for separation. The mobile phase was acetonitrile - 5 mmol per L ammonium formate in water (0.1% acetic acid) and the rate of acetonitrile gradually increased from 5% to 40% in 10 minutes with a 0.4 mL per minute1 flow rate. The MS/MS conditions: nebulizer gas and drying gas were N_2 (purity > 99.999%) with the flow rate of 3.0 L per minute and 15.0 L per min; interface was ESI source; desolvation line (DL) and heat block temperature were 250 °C and 400 °C respectively; interface voltage was set 4.5 kV; CID gas was Ar, purity > 99.999%), multiple reaction monitoring (MRM) mode was set for determination. The results were show in **Supplementary Fig. 2**.

Supplementary Method 3. Karyotyping for three *Papaver* species

The plant seeds were washed and placed in a culture dish with moist filter paper in an incubator at 25℃ to allow germination until the root grew to about 1 cm. For karyotyping, about 0.5 cm fresh root tips were cut off in the morning, and immediately placed in a 0.004 M 8-hydroxyquinoline solution for 4 hours in darkness, at room temperature. Then root tips were fixed in Carnoy's fluid (absolute ethanol: acetic acid = 3: 1 V/V) overnight, and stored in 70 % ethanol at 4℃ for further studies. In order

to achieve best separation of the chromosomes at metaphase, the root tips were thoroughly washed with distilled water, and then macerated in 1 M HCl for 9 minutes at 60℃ for acid hydrolysis. After dissociation, the root tips were put in distilled water for 15 minutes for hypotension, then the root tips were stained by improved carbol-fuchsin solution for about 10 minutes, and squashed on a glass slide. Finally, chromosomes were examined with a microscope (Olympus CX23, Japan) and photographs were taken, the software photoshop 7.0 was employed for karyotypic analysis. The karyotyping results of three *Papaver* species show in **Supplementary Fig. 1**. We repeated three times for each karyotyping experiment independently with similar results. The experiment results confirmed the karyotype of *P. somniferum* is 2n = 22[1], the karyotype of *P. setigerum* is 2n = 44[2,3], and the karyotype of *P. rhoeas* is 2n = 14[4,5].

Supplementary Method 4. Flow cytometry

To determine the DNA quantity of *Papaver setigerum* and *P. rhoeas* genomes, flow cytometry of nuclei was conducted using a modified version of a previously described method[6]. Basically, one to two young fresh leaves (equivalent to 300–500 mg) of *P. setigerum, P. rhoeas* and *P. somniferum* (internal reference) were collected from four weeks old seedlings, and placed into a 100 mm Petri dish. Then 1.5mL of nuclei isolation buffer was added, and the two types of tissue were chopped simultaneously with a razor blade for 30 s (~60 chops per sample) to release the nuclei. The resulting homogenate was filtered through a 48 μm nylon filter into a 1.5 mL tube. Then, the nuclear suspension was stained with 10 μL of propidium iodide (10 mg/mL), and 10 μL of RNase A (10 mg/mL) was added immediately to prevent the staining of a double-standard RNA. The samples were incubated on ice for 10 minutes. Then, the aqueous suspension of intact nuclei from the samples and the internal reference DNA standard were analyzed on a NovoCyte machine (ACEA Biosciences, Inc.) with NovoExpress software (Version 1.2.4.1602). A green argon laser at a wavelength of 488 nm was used as the light source, and the flow of at least 10000 nuclei was measured in the sample.

Supplementary Method 5. DNA and RNA Preparation for sequencing

Preparation of genomic DNA for Nanopore long-read sequencing

High molecular weight DNA isolation

High molecular weight (HMW) genomic DNA was prepared by the CTAB method and purified with QIAGEN® Genomic kit (Cat#13343, QIAGEN) for regular DNA sequencing following the standard operating procedure recommended by the manufacturer. Ultra-long DNA was extracted by the SDS method[7] without purification step to sustain the length of DNA. The integrity of the extracted DNA was monitored on 1% agarose gels, and DNA purity was then determined using NanoDrop™ One UV-Vis spectrophotometer (Thermo Fisher Scientific, USA), of which OD260/280 ranging

from 1.8 to 2.0 and OD 260/230 is between 2.0-2.2. At last, DNA concentration was further measured by Qubit® 4.0 Fluorometer (Invitrogen, USA).

Library preparation and sequencing

For regular Oxford Nanopore (ONT) sequencing, 4 μg HMW DNA was used as input material for the ONT library preparations. Size-select of long DNA fragments for qualified samples were performed using the PippinHT system (Sage Science, USA). Next, the ends of DNA fragments were repaired, and A-ligation reaction was performed with NEBNext Ultra II End Repair/dA-tailing Kit (New England Biolabs Cat# E7546). The adapter in the SQK-LSK109 (Oxford Nanopore Technologies, UK) was used for further ligation reaction and DNA library was measured by Qubit® 4.0 Fluorometer (Invitrogen, USA). About 700ng DNA library was constructed and sequenced on a Nanopore PromethION sequencer (Oxford Nanopore Technologies, UK) at the Genome Center of Grandomics (Wuhan, China). For ONT ultra-long sequencing, approximately 10 μg of ultra-long gDNA was size selected (>50 kb) with SageHLS HMW library system (Sage Science, USA), and processed using the Ligation sequencing 1D kit (SQK-LSK109, Oxford Nanopore Technologies, UK) according the manufacturer's instructions. About 800ng DNA libraries were constructed and sequenced on the PromethION (Oxford Nanopore Technologies, UK) at the Genome Center of Grandomics (Wuhan, China). After sequencing, Guppy (version 3.2.2) was used to basecalling with parameter '-c dna_r9.4.1_450bps_fast.cfg'. The raw reads were trimmed of sequence adaptor, and consider the reads with 'mean_qscore_template' larger than 7 as 'pass reads'.

For *P. setigerum*, six and four ONT cells were used for ONT regular sequencing and ultra-long read sequencing respectively. In total, 15 million ONT reads passed the quality control as 'pass reads' (~394 Gb, 86X coverage) with N50 of around 30 Kb, and a maximum read length of 270 Kb, and three million ONT ultra-long reads were 'pass read' (~88 Gb and 19X coverage) with N50 of around 45kb, and a maximum read length of 674 Kb (**Supplementary Data 1**). For *P. rhoeas*, four ONT cells were used for both ONT regular sequencing and ONT ultra-long read sequencing. In total, about 8 million ONT reads were 'pass reads' (~168 Gb, 66X coverage) with N50 of around 30 Kb, and a maximize read length of 293 Kb, and a total of 853 thousand ONT ultra-long reads were 'pass read' (~33 Gb and 13X coverage) with N50 of around 80 Kb, and a maximize read length of 512 Kb (**Supplementary Data 1**).

Preparation of genomic DNA for Illumina paired-end read sequencing

A total amount of 1.5μg DNA was used for constructing sequencing libraries, which were generated using Truseq Nano DNA HT Sample Preparation Kit (Illumina USA) following manufacturer's recommendations. The libraries constructed above were sequenced by Illumina NovaSeq platform to generate 150bp paired-end reads with insert size around 400bp. Reads with adaptors, and low-quality bases at 5' and 3'-end were trimmed afterwards. In addition, duplicated reads, reads with more than 10% bases marked as 'N', and reads with more than 50% low quality bases were filtered.

For *P. setigerum*, about 2,127 million Illumina paired-end reads were generated (~319 Gb and 71X coverage). Of them, the quality scores of around 90% bases are larger than Q30. For *P. rhoeas*, about 1 million cleaned Illumina paired-end reads were generated (~150 Gb and 59X coverage). Of them, the quality score of 93% bases were larger than Q30 (**Supplementary Data 1**).

Hi-C sequencing

Leaves were fixed with 1% formaldehyde solution in MS buffer (10 mM potassium phosphate, pH 7.0, 50 mM NaCl; 0.1M sucrose) at room temperature for 30 minutes in a vacuum. After fixation, the leaves were incubated at room temperature for 5 minutes under vacuum in MC buffer with 0.15 M glycine. Approximately two grams of fixed tissue was homogenized with liquid nitrogen and resuspended in nuclei isolation buffer and filtered with a 40-nm cell strainer. The enrichment of nuclei from flow-through and subsequent denaturation were done according to a previous 3C protocol established for maize[8]. The chromatin extraction and library construction were performed following a procedure described previously[9]. Briefly, chromatin was digested for 16 h with 400 U HindIII restriction enzyme (NEB) at 37 °C. DNA ends were labeled with biotin and incubated at 37 °C for 45 min, and the enzyme was inactivated with 20% SDS solution. DNA ligation was performed by the addition of T4 DNA ligase (NEB) and incubation at 16°C for 4~6 h. After ligation, proteinase K was added to reverse cross-linking during incubation at 65 °C overnight. DNA fragments were purified and dissolved in 86μL of water. Unligated ends were then removed. Purified DNA was fragmented to a size of 300–500 bp, and DNA ends were then repaired. DNA fragments labeled by biotin were finally separated on Dynabeads® M-280 Streptavidin (Life Technologies). Hi-C libraries were controlled for quality and sequenced on an Illumina Novoseq sequencer. To avoid reads with artificial bias, we removed the following type of reads: (a) Reads with over 10% unidentified nucleotides; (b) Reads with more than ten nucleotides aligned to the sequencing adapters, allowing fewer than 10% mismatches; (c) Reads with over 50% bases having Phred quality lower than 5; (d) Putative PCR duplicates generated in the library construction. As a result, about five million (~765 Gb, 282X coverage), four million (~655 Gb, 143X coverage) and two million Hi-C clean reads (~356 Gb, 140X coverage) were generated for *P. somniferum*, *P. setigerum*, and *P. rhoeas*, respectively (**Supplementary Data 1**).

RNA isolation and transcriptome sequencing

Total RNA was extracted by grinding tissue in TRIzol reagent (TIANGEN) on dry ice and processed following the protocol provided by the manufacturer. The integrity of the RNA was determined with the Agilent 2100 Bioanalyzer (Agilent Technologies) and agarose gel electrophoresis. The purity and concentration of the RNA were determined with the Nanodrop (Thermo Fisher Scientific) and Qubit (Thermo Fisher Scientific). Only high-quality RNA sample (OD260/280 within range [1.8, 2.2], OD260/230 ≥ 2.0, RIN ≥ 8, > 1 μg) was used to construct sequencing library. For transcriptome sequencing, a total amount of 1 μg RNA per sample was used as input material for the

RNA sample preparations. Sequencing libraries were generated using TruSeq RNA Library Preparation Kit (Illumina, USA) following manufacturer's recommendations. The library preparations were sequenced on an Illumina Novaseq platform and paired-end reads of 150 bp were generated. We generated the RNA-seq data from six tissues harvested on the first day of anthesis, including capsule, stamen, petal, stem, leaf, and root, for *P. setigerum* and *P. rhoeas*. For *P. setigerum*, we sequenced about 60 million RNA-seq reads for each tissue, of which more than 90% had a quality score of larger than Q30. For *P. rhoeas*, we sequenced about 60 million RNA-seq reads for each tissue, of which more than 93% reads had a quality score of larger than Q30 (**Supplementary Data 1**).

Supplementary Method 6. Genome assembly and evaluation

Genome size estimation

We estimated genome size of *P. setigerum* and *P. rhoeas* based on whole genome Illumina paired-end sequencing data using kmer frequency analysis with k = 17 following Lander Waterman algorithm: $G = K_{num} / K_{depth}$, where K_{num} denotes the number of kmer, and K_{depth} denotes the depth of kmer[10]. For *P. setigerum*, we obtained 154,497,968,202 kmers, and the depth is 32 (**Supplementary Fig. 3b**). Therefore, the estimated genome size is 4.82 Gb. For *P. rhoeas*, we obtained 131,867,393,561 kmers, and the major peak depth is 55 (**Supplementary Fig. 5b**). Therefore, the estimated genome size is 2.31 Gb. In addition, we found a second kmer peak in *P. rhoeas*, indicating it's a complex genome with high heterozygosity (**Supplementary Fig. 5b**). To estimate its heterozygosity rate, we simulated 57X *Arabidopsis thaliana* Illumina paired-end sequencing data using pIRS (https://github.com/galaxy001/pirs) software[11] with heterozygosity from 1% to 5% with a step of 0.01% based on *Arabidopsis thaliana* TAIR10.1 reference genome (https://www.ncbi.nlm.nih.gov/assembly/GCF_000001735.4). We found the kmer (*k* = 17) frequency distribution of simulated *Arabidopsis thaliana* data of around 3.18% heterozygosity rate fitted with that of *P. rhoeas* data of the same peak depth, indicating the heterozygosity rate of *P. rhoeas* was around 3.18% (**Supplementary Fig. 5b**).

The command for simulation runs like following:

```
pirs diploid -i $REF -s $hr -o $REF.simulation.fa.gz
pirs simulate -l 150 -m 500 -i $REF -I $REF.simulation.fa.gz -x 57 -e 0.001 -Q 33
```

Genome assembly

The sequence data used for *de novo* assembly of *P. setigerum* and *P. rhoeas* included regular and ultra-long ONT reads, Illumina paired-end reads, and the Hi-C reads. For *P. somniferum*, the improved assembly was based on the published draft genome

assembly[1] and newly generated Hi-C reads.

We assembled the genome contigs by NextDenovo (v2.2)[12] (https://github.com/Nextomics/NextDenovo) software with parameters seed_cutoff = 35k, reads_cutoff = 1k for *P. rhoeas* and seed_cutoff = 33k, reads_cutoff = 1k for *P. setigerum* based on the ONT reads. NextCorrect and NextGraph are two major steps in genome assembly using NextDenovo. NextCorrect corrects raw reads to generate the consensus reads, and NextGraph assemblies the consensus reads to generate the primary contigs. After primary contigs generated, ONT reads were firstly used to polish the primary contigs in three rounds, and the Illumina paired-end reads were used to further polish the contigs in four rounds. All polishing processes were achieved by Nextpolish (v1.2.0)[13]. For P. rhoeas, we further applied purge_dups[14] (https://github.com/dfguan/purge_dups) to reduce redundancy in the polished contigs with cutoffs as '5 34 56 67 112 201', which is automatically calculated by calcuts module in purge_dups. Then breakhic (v1.1) (https://github.com/wtsi-hpag/scaffHiC) was used to identify assembly breakpoints of polished contigs by screening paired Hi-C reads. Finally, 3d-DNA (v180922)[15] (https://github.com/aidenlab/3d-dna) pipeline was used to reorder and anchor contigs into scaffolds and chromosomes. The scaffolds and chromosomes were subjected to a final n expert manual check to correct misassembles.

We applied the assembly pipeline on *P. setigerum* and *P. rhoeas* data. For *P. setigerum*, we assembled the genome size was 4.59 Gb with scaffold N50 was 211.16 Mb and contig N50 was 65.573 Mb. Most (97.55%) assembled sequences were assembled on 22 chromosomes. (**Supplementary Fig. 4**) For *P. rhoeas*, we assembled the genome size was 2.54 Gb with scaffold N50 was 329.41 Mb and contig N50 was 5.29 Mb. Most (87.87%) assembled sequences were assembled on 7 chromosomes. (**Supplementary Fig. 6**) For *P. somniferum*, we first applied breakhic to the published HN1 genome[1] and then re-scaffolded the contigs by 3d-DNA based on Hi-C data, followed by manual checks to identify, and correct misassembles. After improving, we assembled the genome size is 2.71 Gb with scaffold N50 improving from 204.47 Mb to 249.6 Mb and contig N50 being 1.74 Mb. 92.37% assembled sequences, which is much improved over the published one (81.6%)[1], were assembled on 11 chromosomes (**Supplementary Fig. 7**). The details of the genome size, scaffold N50, and contig N50 of each assembly step were show in **Table 1** and **Supplementary Data 2**.

Assembly evaluation

To evaluate the completeness of three *Papaver* genome assemblies, we applied Benchmarking Universal Single-Copy Orthologs (BUSCO) (v3) using the plant early release version (v1.1b1, release May 2015)[16] to evaluate the completeness of genome assemblies. The BUSCO test reports 92.8%, 95.3% and 94.5% of complete gene models for *P. rhoeas*, *P. somniferum* and *P. setigerum*, respectively (**Supplementary Fig. 8**), suggesting the high completeness of three *Papaver* genome assemblies. Furthermore, we aligned the Illumina paired-end reads to the assembled genome by BWA (v0.7.17-

r1188)[17] with default parameters, and calculated the read depth by SAMTools (version 1.9)[18]. We found that the mean coverage of 87.89%, 98.51%, and 97.17% assembled sequences were larger than 5 in *P. rhoeas*, *P. setigerum*, and *P. somniferum*, confirming their high completeness. To validate assembly base accuracy, we detect SNPs and Indels from the Illumina paired-end reads alignment BAM file by GATK (version 4.1.8)[19] of three *Papaver* species.

The command to detect SNPs and Indels like following:

```
gatk HaplotypeCaller -R ref.fa -ERC GVCF   -I sample.bam -O sample.g.vcf.gz
gatk GenotypeGVCFs -R ref.fa -V sample.g.vcf.gz -O sample.raw.vcf.gz
gatk SelectVariants -V sample.raw.vcf.gz -select-type SNP -O sample.raw.SNV.vcf.gz
gatk VariantFiltration   -V sample.raw.SNV.vcf.gz \
                -filter "QD < 2.0"   --filter-name "QD2" \
                -filter "QUAL < 30.0" --filter-name "QUAL30" \
                -filter "SOR > 3.0" --filter-name "SOR3" \
                -filter "FS > 60.0" --filter-name "FS60" \
                -filter "MQ < 40.0" --filter-name "MQ40" \
                -filter "MQRankSum < -12.5" --filter-name "MQRankSum-12.5" \
                -filter "ReadPosRankSum < -8.0" --filter-name "ReadPosRankSum-8"
\
                -O sample.pass.SNV.vcf.gz
gatk SelectVariants -V sample.raw.vcf.gz -select-type INDEL -O
sample.raw.INDEL.vcf.gz
gatk VariantFiltration \
                    -V sample.raw.INDEL.vcf.gz \
                    -filter "QD < 2.0"   --filter-name "QD2" \
                    -filter "QUAL < 30.0" --filter-name "QUAL30" \
                    -filter "FS > 200.0" --filter-name "FS200" \
                    -filter "ReadPosRankSum < -20.0" --filter-name
"ReadPosRankSum-20" \
                    -O sample.pass.INDEL.vcf.gz
```

For *P. rhoeas*, we detected 544,211 homozygous SNPs and 864,632 homozygous Indels at read depth larger than five, suggesting the assembly base accuracy is 99.9% and the quality value is Q30. For *P. setigerum*, we detected 270,130 homozygous SNPs and 351,419 homozygous Indels at read depth larger than five, indicating the assembly base accuracy is 99.99% and the quality value is Q40. For *P. somniferum*, we detected 169,654 homozygous SNPs and 65,322 homozygous Indels at read depth larger than five, indicating the assembly base accuracy is 99.99% and the quality value is Q40.

The contiguity of genome assembly is usually affected by multiple factors, such as heterozygosity rates, polyploidy, raw reads quality, repeat content in genome etc. In our study, raw reads quality and genome heterozygosity are two main reasons on the rates of assignment to the scaffolds of three *Papaver* species. We have high quality raw reads

for all three species (**Supplementary Data 1**), *e.g.* the ONT raw reads N50 are about 30Kb in both *P. setigerum* and *P. rhoeas*, the Q30 of Hi-C data for *P. setigerum*, *P. rhoeas*, and *P. somniferum* are larger than 91%. The main difference of three *Papaver* species is the heterozygosity rate. *P. setigerum*, despite its large genome size, has a low heterozygosity rate, as shown by a lack of clear heterozygosity peak in the k-mer frequency distribution of *P. setigerum* sequencing reads (**Supplementary Fig. 3**). By contrast, despite the relatively smaller genome size, *P. rhoeas* has a high heterozygosity rate of 3.18% as shown by a clear heterozygosity peak in the k-mer frequency distribution of *P. rhoeas* sequencing reads (**Supplementary Fig. 5**). Therefore, although the read length, quality of sequencing data and assembly methods for both genomes are similar, the genome assembly contiguity differed a lot.

Supplementary Method 7. Genome annotation

Annotation of repetitive elements

We used Repbase[20] and the species-specific *de novo* constructed repeat library to annotate the repetitive elements in three *Papaver* species. Repbase was downloaded from http://www.girinst.org/repbase/ and the species-specific *de novo* repeat library was constructed by RepeatModeler (vopen-1.0.8, http://repeatmasker.org/RepeatModeler/) . RepeatMasker (vopen-4.0.7, http://www.repeatmasker.org/RepeatMasker/) was applied to annotated the repeat elements. In addition, we applied LTR_Finder (v1.1, https://github.com/xzhub/LTR_Finder)[21], LTRHarvest (v1.5.9, http://genometools.org/)[22] and LTR_retriever (v2.8.5, https://github.com/oushujun/LTR_retriever)[23] to detect LTR elements. The length distribution of the repetitive elements like a uniform distribution with mean value around 1 kb in three *Papaver* species (**Supplementary Fig. 11**). The most abundant repetitive element type is long terminal repeat (LTR) in three *Papaver* species, making up 50.5%, 56.12% and 54.9T of the *P. rhoeas*, *P. somniferum*, and *P. setigerum* genome respectively. The major LTR types are Copia and Gypsy in all three species (**Supplementary Fig. 11**).

Protein-coding gene prediction and functional annotation

Protein-coding genes were predicted using the MAKER2 pipeline (v2.31.8)[24] in three *Papaver* species. In short, MAKER2 first masked repetitive elements in the assembled genomes using RepeatMasker (http://repeatmasker.org/). Then, both evidence-based and *ab initio* gene predictors were applied to predict protein-coding genes. For the evidence-based model, MAKER2 uses Blast algorithms to align protein and transcripts data to the genome. The alignments were further polished by Exonerate to produce gene models[25]. MAKER2 performed the ab initio gene prediction based on the assembly sequence itself and then compared predicted gene models to those determined by transcripts and protein alignment to revise the gene predictions. The confidence of each

predicted gene model was then measured using the Annotation Edit Distance (AED) and exon AED (eAED) method, which quantified the normalized distance between gene model and its supporting evidence.

Three ab initio gene predictors were used: AUGUSTUS (v3.3)[26], SNAP (v2006-07-28)[27] and GeneMark_ES (v3.48)[28]. Tomato (*Solanum lycopersicum*) was used as species model for the AUGUSTUS gene prediction, and the pre-trained model of *Arabidopsis thaliana* was used as input for the Hidden Markov Models of SNAP and GeneMark_ES. Swiss-Prot (in January 2020) was downloaded (https://www.uniprot.org/downloads), and protein sequences of three species, *A. thaliana*[29], *Beta vulgaris*[30] and *Vitis vinifera*[31] were obtained from the Ensembl Plants database (http://plants.ensembl.org/index.html). Transcripts were *de novo* assembled by Trinity (v2.1.1)[32] using the RNA-seq data of three species.

MAKER2 pipeline initially predicted 161,909, 312,318, and 148,269 candidate gene models in *P. rhoeas*, *P. setigerum*, and *P. somniferum*, respectively. We filtered these genes to produce a high-confidence annotated gene sets of 41,470, 106,517, and 55,316 genes in *P. rhoeas*, *P. setigerum*, and *P. somniferum*, respectively **(Table 1, Supplementary Fig. 9)** using the following criteria: 1). genes lacking transcript or protein homolog support; 2). genes with AED or eAED larger than 0.5; 3). genes overlapped with annotated ncRNAs (non-coding RNAs). Annotation features such as length distribution of gene, transcript, protein sequence and exon number distribution are shown in **Supplementary Fig. 9**. We functionally annotated the predicted protein-coding genes using InterProScan (v5.25-64.0) with default parameters[33]. In total, about 70.14%, 70.56%, and 70.05% predicted genes of *P. rhoeas*, *P. setigerum*, and *P. somniferum,* respectively, have annotated functional domains.

Non-coding RNA annotation

Non-coding RNAs (ncRNAs) were annotated using cmscan from INFERNAL (v1.1.2) package[34] based on Rfam database (v14.1) (ftp://ftp.ebi.ac.uk/pub/databases/Rfam/14.1/Rfam.cm.gz)[35]. Firstly, we created the index of Rfam database by the command ‘cmpress Rfam.cm’. Then, we predicted the ncRNAs using cmscan based on the indexed Rfam database as following command. Finally, we predicted 12,429, 23,109, and 12,636 ncRNAs in *P. rhoeas*, *P. setigerum*, and *P. somniferum*, respectively (**Table 1, Supplementary Fig. 10**) and classified the ncRNAs into different class, e.g. miRNA, snoRNA, rRNA, tRNA using class information from http://rfam.xfam.org/search#tabview=tab4.

```
cmscan -Z $genome_size --cut_ga --rfam --nohmmonly --tblout $out_sign.tblout --fmt 2 --clanin $RFAMDIR/Rfam.clanin --cpu $t $RFAMDIR/Rfam.cm $REF > $out_sign.cmscan
grep -v '=' $out_sign.tblout >$out_sign.deoverlapped.tblout
```

Supplementary Method 8. Genome synteny analysis

Whole genome duplication events

To study the evolution of three *Papaver* genomes, we investigated the genome-wide duplications in our chromosomal-scale assemblies of *P. rhoeas*, *P. setigerum*, and *P. somniferum*. Firstly, we performed synteny analysis within each species. We performed intraspecies all-vs-all paralog analysis in three genomes by BlastP using annotated protein sequences. MCScanX[36] were then ran with default parameters from top-five self-BlastP hits. We detected 290 synteny blocks including 3,929 syntenic gene pairs and 7,181 genes (17.3%) in *P. rhoeas*, 2,908 synteny blocks including 89,225 syntenic gene pairs and 71,351 genes (67.0%) in *P. setigerum*, and 647 synteny blocks including 16,944 syntenic gene pairs and 29,009 genes (52.4%) in *P. somniferum*. We found the majority (61.9%) of the syntenic gene pairs are located intra-chromosomally in *P. rhoeas*, *i.e.*, 406 within chromosome 1, 380 within chromosome 2. While in *P. setigerum* and *P. somniferum*, we found 98.2% and 91.6% syntenic gene pairs are located inter-chromosomally, respectively. These results suggested the occurrence of one WGD event in *P. somniferum*, two WGD events in *P. setigerum* but segmental duplication rather than WGD in *P. rhoeas*. We calculated the synonymous substitution rate (Ks) of each syntenic gene pair in three *Papaver* species by KaKs_Calculator (v2.0)[37], and found major peaks at around 0.1 in *P. setigerum* and *P. somniferum* but no dominant peak in *P. rhoeas* (**Fig. 1d**), confirming the WGDs in *P. setigerum* and *P. somniferum*. In addition, the widespread and well-maintained two copies of the syntenic blocks in *P. somniferum* and four copies of the syntenic blocks in *P. setigerum* indicated one WGD in *P. somniferum* and two WGDs in *P. setigerum* (**Supplementary Fig. 12**). Indeed, analysis of gene duplication types of the *P. somniferum* and *P. setigerum* and *P. rhoeas* paralogs by MCScanX indicate that WGD/segmental duplication is the dominant type in *P. somniferum* and *P. setigerum*, while dispersed duplication is the dominant type in *P. rhoeas* (**Supplementary Fig. 14**), confirming WGDs in *P. somniferum* and *P. setigerum* while no WGD in *P. rhoeas*. Of genes with the WGD/segmental duplication types, we found 91% exist as two copies in *P. somniferum* and 45% exist as four copies in *P. setigerum* (**Supplementary Fig. 15**), further confirming one WGD in *P. somniferum* and two WGDs in *P. setigerum*. We did synteny analysis between the three genomes by MCScanX, and found a clear 1:2:4 synteny relations (**Fig. 1c and Supplementary Fig. 13**), providing additional evidence for no WGD in *P. rhoeas*, one WGD in *P. somniferum*, and two WGDs in *P. setigerum*.

For core eudicots such as *Vitis vinifera* (grape), a γ hexaploidization event occurred before divergence of Rosids and Asterids. Grape is often used as a reference for investigating the evolutionary history of eudicot genomes since its genome underwent no further whole genome duplications except only a few minimal rearrangements following the γ event[31,38]. Synteny analysis using three *Papaver*

genomes and grape genome suggested that the three *Papaver* species did not experience the γ event as suggested by a 3:1, 3:2, and 3:4 syntenic relationships between grape and *P. rhoeas*, grape and *P. somniferum*, and grape and *P. setigerum*, respectively (**Supplementary Fig. 17**). Murat *et al*. constructed the genome of the most recent ancestor of flowering plants, referred to as the ancestral eudicot karyotype (AEK)[38]. We compared the three *Papaver* genomes to AEK in addition to the grape genome. The synteny dot plots and genome painter images (**Supplementary Fig. 18**) illustrated that most AEK and grape segments have one, two, and four syntenic copies in *P. rhoeas*, *P. somniferum*, and *P. setigerum*, suggesting that *P. rhoeas* had no WGD event, while *P. somniferum* and *P. setigerum* clearly underwent one and two WGD events, respectively. Moreover, we calculated the ortholog depth of *P. rhoeas*, *P. somniferum* and *P. setigerum* per AEK and grape genes from the synteny analysis (**Supplementary Figs. 17, 18**). We found a dominant peak (2,372 (75%) and 5,771 (77%) collinear genes in AEK and grape, respectively) at depth of one in *P. rhoeas*, while a dominant peak (1,665 (48%) and 4,972 (53.1%) collinear genes in AEK and grape, respectively) at depth of two in *P. somniferum*, and a high peak (781 (21.3%) and 2,889 (29%) collinear genes in AEK and grape, respectively) at depth of four in *P. setigerum*. Taken together, our analysis provides strong evidence for no WGD event in *P. rhoeas* genome, one WGD event in the *P. somniferum* genome, and two WGD events in the *P. setigerum* genome.

Phylogenomic analysis and divergence time estimation

To investigate the evolutionary history of three *Papaver* genomes, we conducted phylogenomic analysis of three *Papaver* genomes with other five angiosperm species including the monocot *Oryza sativa*[39] (http://plants.ensembl.org/Oryza_sativa/Info/Index), *Aquilegia coerulea* (*A. coerulea*)[40] (https://phytozome.jgi.doe.gov/pz/portal.html), *Macleaya cordata*[41] (GenBank accession: GCA_002174775.1), *Arabidopsis thaliana* (*A. thaliana*)[42] (http://plants.ensembl.org/Arabidopsis_thaliana/Info/Index), and *Vitis vinifera* (*V. vinifera*)[31] (http://plants.ensembl.org/Vitis_vinifera/Info/Index). Single-copy orthologs are commonly used to achieve robust phylogenetic reconstruction with high confidence and concordance. Applying OrthoFinder *v.2.3.4*[43] we detected 48 single-copy orthologs from eight angiosperm genomes. To construct a phylogenetic tree, single-copy ortholog pairs were aligned with MAFFT (v7)[44], and the conserved sites in the alignments were further extracted using Gblocks (v0.91b)[45] with the default parameters, followed by the maximum likelihood phylogenomic tree construction with RAxML (v8.2.12)[46] with 100 bootstraps (**Fig. 1e**). The divergence times between species were estimated using the Penalized likelihood method and parameter of 'setsmoothing = 1000' with r8s v.1.8[47], based on the constructed phylogenetic tree and the fixage times of monocot-dicot split time (152 Mya, http://timetree.org/), constrain taxon time of *Aquilegia-Papaver* (127.9~139.4 Mya, http://timetree.org/)[48], and constrain taxon time of *A. thaliana* and *V. vinifera* (107~135 Mya, http://timetree.org/). We estimated the *P. rhoeas and P. somniferum* diverged time at around 7.7 Mya, consisting with timetree website (http://timetree.org/) reports. In addition, we estimated the divergence time of *P.*

somniferum and *P. setigerum* is 4.9 Mya.

Time estimation of whole genome duplications

To estimate the timing of the WGD event in *P. somniferum* and *P. setigerum*, *Ks* values of *P. somniferum* and *P. setigerum* syntenic block genes were calculated using YN model in KaKs_Calculator (v2.0)[37]. *P. setigerum* underwent two WGDs. We considered the reciprocal best matches among the syntenic gene pairs were as the pairs from WGD-2 (the second WGD event) while other syntenic gene pairs were grouped as the pairs from WGD-1 (the first WGD event). The Ks value distributions were then fitted to a mixture model of Gaussian distribution (**Fig. 1d, Supplementary Data 3**) using the Mclust R package[49]. We identified components associated with WGD peaks and calculated their mean and standard deviation of Ks values. To time the WGDs in three *Papaver* species, we estimated the average evolutionary rate for *Papaverance* using *P. somniferum* and *P. rhoeas*. Divergence time between *P. somniferum* and *P. rhoeas* is estimated as 7.7 Mya. Given the mean Ks value (0.12) of *P. somniferum*-*P. rhoeas* and their divergence date T (7.7 Mya), we calculated the synonymous substitutions per site per year (r) for *Papaveraceae* as 8.08e-9 ($T = Ks/2r$) (**Supplementary Data 3**) which was applied to time the WGDs of *P. somniferum* and *P. setigerum*. We dated the WGD in *P. somniferum* (Ks = 0.116 ± 0.028) around 7.2 ± 1.7Ma, the first WGD in *P. setigerum* (Ks = 0.115 ± 0.018) around 7.1 ± 1.1 Mya, and the second WGD in *P. setigerum* (Ks = 0.065 ± 0.017) around 4.0 ± 1.0 Mya (**Fig. 1d, Supplementary Data 3**). The divergence time between *P. somniferum* and *P. setigerum* is around 4.9 Mya, later than the WGD-1 in *P. setigerum* (7.1 Mya) and the WGD in *P. somniferum* (7.2 Mya) and earlier than WGD-2 in *P. setigerum* (4.0 Mya), indicating the WGD-2 is a *P. setigerum* specific event while WGD-1 is shared by *P. somniferum* and *P. setigerum* (**Fig. 1d**). The previously reported WGD/WGT (whole genome triplication) events in five other angiosperm species (*N. nucifera* (65 Mya), *O. sativa* (66 Mya), *A. thaliana* (67 Mya), and *A. coerulea* (110 Mya) are displayed in the phylogenetic tree (**Fig. 1d**).

Protein coding gene number comparison based on synteny analysis

The protein coding gene numbers are quite comparable between *P. somniferum* and *P. rhoeas*, while the former have undergone WGD while the latter not. To investigate the functions of species-specific genes, we performed the syntenic analysis of three *Papaver* species, and found 28,660 genes in *P. somniferum* were syntenic with 19,512 genes in *P. rhoeas* with syntenic depth from one to five (**Supplementary Fig. 16a**), indicating that 28,660 genes are kept in *P. somniferum* following WGD-1 and diploidization. For any two-species comparison, it is difficult to differentiate gene 'gain' and 'loss' because gain for one species means loss for the other species, and *vice versa*. Alternatively, we found 21,958 and 26,654 genes are specific to *P. rhoeas* and *P. somniferum* respectively, by comparing *P. rhoeas* and *P. somniferum* genes. We performed the functional enrichment for species-specific genes to understand their functional roles. Based on the functional enrichment analysis, and the *P. somniferum* specific genes were significantly enriched in energy, photosynthesis, and metabolism

related pathways, while *P. rhoeas* specific genes were significantly enriched in oxidative phosphorylation, ubiquitin system, ABC transporters related pathways (**Supplementary Fig. 16c**).

Similarly, we compared *P. somniferum* with *P. setigerum,* and found 41,073 genes in *P. somniferum* were syntenic with 71,398 genes in *P. setigerum* with synteny depth from one to 11 (**Supplementary Fig. 16b**), indicating that 71,398 genes in *P. setigerum* were related with WGD-2, while 14,241 genes in *P. somniferum* were specific and 35,119 genes in *P. setigerum* were specific based on the comparison between *P. somniferum* and *P. setigerum*. The functional showed the *P. somniferum* specific genes were significantly enriched in photosynthesis, ribosome, metabolism related pathways, while *P. setigerum* specific genes were significantly enriched in Spliceosome, metabolism, Endocytosis related pathways (**Supplementary Fig. 16d**).

Gene family evolution analysis

To understand the genomic basis of adaptation evolution in *Papaver*, we compared *P. somniferum, P. setigerum* and *P. rhoeas* genomes with other five representative angiosperm genomes, *Aquilegia coerulea, Macleaya cordata, Vitis vinifera, Arabidopsis thaliana,* and *Oryza sativa,* and identified *Papaver* gene families that have gone through significant expansion and contraction using OrthoFinder (v2.3.4) and CAFE (v3)[50]. CAFE was used to test whether protein family sizes were compatible with a stochastic birth and death model, and the Viterbi algorithm in the CAFE program was determine the significance of expansions/contractions experienced at each branch with a cutoff of p-value < 0.05. Among 27,386 orthogroups (gene families) in eight plant species, 466 ,58, 152 have gone through significant expansion in *P. setigerum, P. somniferum* and *P. rhoeas* (p-value < 0.05), respectively. Enrichment of Pfam domains by FunRich (v3.1.3)[51] using the expanded families in *P. setigerum* and *P. somniferum* suggests enriched Pfam domains such as cytochrome P450, 2-oxoglutarate (2OG) and Fe (II)-dependent oxygenase, key enzymes in plant specialized metabolism, and major latex protein, wound-associated kinase. *P. setigerum* and *P. rhoeas* were also enriched in NB-ARC domain proteins and receptor-like protein kinases that are important players in defense responses (**Supplementary Data 6-8**). This suggests *Papaver* genomes have gone through gene family expansions that facilitate adaptive evolution in coping with environmental stresses through secondary metabolism and defense response.

Supplementary Method 9. Inferring the most recent common ancestor for *Papaver* and downstream analysis

To reconstruct the pre- and post-WGD ancestor genomes, we proposed a computational workflow containing three stages including synteny block reconstruction, inferring

ancestral protochromosomes and inferring gene orders in ancestors **(Supplementary Fig. 19)**. We also conducted downstream analysis to investigate the functions of post WGD genome rearrangements (**Fig. 2**). Our workflow is based on the accuracy of genome assembly. But now even with the cutting-edge sequencing data and widely used assembly methods, assembly errors are inevitable[52]. The potential misassembly may affect the reconstruction. Therefore, computational evaluation and experimentally validation of genome assemblies are important to obtain more reliable results.

Synteny block reconstruction

In the first stage, we attempted to detect syntenic blocks between *P. rhoeas*, *P. somniferum* and *P. setigerum*. First, we preformed sequence alignment using protein sequences from the three species by BlastP with e-value threshold of 1e-5. Secondly, ortholog gene pairs were detected by MCScanX[36] with default parameters. Then, a gene graph was built based on the detected ortholog gene pairs, where nodes denoted genes and edges represented the ortholog relations. We detected each graph component as ortholog gene groups (that is putative protogenes, pPGs) with an assigned identification (ID). For each chromosome of each species, we generated the pPG order based on the gene order, defined as its completed pPG order. We defined a pPG, consisting with four genes from *P. setigerum*, two genes from *P. somniferum*, and one gene from *P. rhoeas*, as a core pPG based on the corresponding numbers of WGD. We filtered non-core pPGs in the completed pPG orders to get the anchor orders (that is core pPG orders). These anchor orders were used to build non-overlapping (NO) synteny blocks using DRIMM-Synteny[53] with parameters of cycle length threshold as 100 and dust threshold as 8. The result of DRIMM-Synteny mainly contains the detected syntenic blocks with identical ID (synteny.txt) and the block orders as well as the directions on each chromosome of three *Papaver* species (blocks.txt). Each block has multiple copies and we defined each copy as a block object. We kept the blocks with four, two and one block object in *P. setigerum*, *P. somniferum* and *P. rhoeas*, respectively. For each chromosome, a core pPG order was generated based on the corresponding block order. We applied a dynamic programming algorithm to find the longest common sequence (LCS) between the new generated core pPG order and the completed pPG order for each chromosome. Based on the LCS, we extracted the pPG order of each block object. Next, we kept the blocks that contain more than five pPGs occurring in all seven copies. Finally, we obtained 30 blocks.

Inferring ancestral protochromosomes

We used the bottom-up strategy to infer each intermediate ancestral protochromosomes following the evolution tree of three species (**Fig. 2**) based on the final 30 synteny blocks. In the three *Papaver* species, *P. setigerum* underwent a lineage-specific WGD (WGD-2) following a shared WGD (WGD-1) with *P. somniferum*, while no WGD in *P. rhoeas* which can make ancestral blocks become multiple copy making it impossible to model the ancestral genomes reconstruction as either a genome median problem (GMP)[54,55] or a guided genome halving problem (GGHP)[56] which are proposed for modeling the ancestral blocks with single copy. Therefore, we proposed a new

reconstructed method. We attempted to use block matching strategy to match block copies in related species first by minimizing the genomic distance and then relabeled block copies to transform problems in *Papaver* species to traditional GMP and GGHP and solved the ancestral states in three *Papaver* species. We built three integer programming solving frameworks including GMP, GGHP and block matching optimazation (MO) based on the single cut or join (SCoJ) distance[57].

SCoJ is defined as:

$$d_{SCoJ} = |\mathbf{A} - \mathbf{B}| + |\mathbf{B} - \mathbf{A}| \quad \textbf{(1)}$$

where $\mathbf{A}$ and $\mathbf{B}$ are adjacency relation lists of synteny blocks. For example, a genome $\{(a,b,c)\}$ has only one chromosome and three blocks. It can be represented as adjacency relation list like $\{\langle a_h, b_t\rangle, \langle b_h, c_t\rangle\}$, which includes two adjacency relations. t represents block tail (start) and h represents block head (end). d_{SCoJ} is the difference between two genome adjacency lists.

But SCoJ does not consider the adjacency with telomeres (chromosome ends), making the ancestor genome more fragmental. So, we added the telomere adjacency in SCoJ to constrain the number of chromosomes. For the above example, the improved adjacency relation list can be represented as $\{\langle \$, a_t\rangle, \langle a_h, b_t\rangle, \langle b_h, c_t\rangle, \langle c_h, \$\rangle\}$, where $\$$ is telomere. Detailed implementation is available on GitHub at https://github.com/XJTU-YeLab/IAG[58].

In order to verify our frameworks, we simulated the evolutionary scenario from top to bottom with two whole genome duplications same with three Papaver species (**Supplementary Fig. 20a**) under infinite sites (IS) assumption, which means a mutation does not occur at the same locus more than once during evolution and is commonly used in evolutionary studies[59]. We simulated block sequences with some random block adjacencies change (default number is five) between each species. Here, we required that the endpoints involved in changes do not overlap to make sure IS assumption. And then, we applied our model to reconstruct each middle species, *e.g.* Species 2, Species 3, and Species 5 in **Supplementary Fig. 20**. We repeated 200 times and found that Species 2 (simulated pre-WGD-1 ancestor) and Species 3 (simulated post-WGD-1 ancestors) can be reconstructed with 100% block adjacency accuracy, and Species 5 (simulated pre-WGD-2 ancestor) can be correctly reconstructed with average 99.68% block adjacency accuracy (**Supplementary Fig. 20b**). This result indicated the accuracy and robustness of our framework under IS assumption.

Then, we applied our model to reconstructing the ancestor of the three *Papaver* species. The first step is inferring pre-WGD-2 ancestor at around 4.0 Mya. We used

MO solving framework to find the 1:2 block matching relation between *P. somniferum* and *P. setigerum* and then split one block in two species with 2:4 block ratio into two blocks with 1:2. Finally, we applied GGHP solving framework. And then, we inferred post-WGD-1 ancestor at around 4.9 Mya based on genomes of pre-WGD-2 ancestor, *P. somniferum* and *P. rhoeas*. We duplicated *P. rhoeas* and applied MO solving framework to convert the block ratio of 2:2:2 to 1:1:1 in three genomes like the first step. GMP solving framework then was used to find the post-WGD-1 ancestral genome. Finally, we directly preformed GGHP solving framework on post-WGD-1 ancestral genome by using *P. rhoeas* as outgroup to find pre-WGD-1 ancestor, since the block ratio is 1:2 in *P. rhoeas* and post-WGD-1.

Next, we evaluated the block adjacency reliability for three ancestors in real *Papaver* evolutionary scenarios. We found all block endpoints in the reconstructed pre-WGD-2 and post-WGD-1 ancestors satisfied IS assumption. We inferred that the block adjacency reliability of both pre-WGD-2 and post-WGD-1 ancestors were 99.68% (pre-WGD-2 ancestor is 99.68% and post-WGD-1 ancestor is 99.68%×100%) based on the simulated results under IS assumption. We adjusted the block adjacency reliability by accumulated multiplication bottom-to-up. However, the pre-WDG-1 ancestor has 11.67% non-IS block endpoint. So, we simulated the pre-WGD-1 ancestor reconstruction under non-IS assumption 1000 times with non-IS block ratio from 0 to 100% (**Supplementary Fig. 20c**). We used quadratic polynomial to fit the correlation between non-IS endpoint rate and endpoint adjacency inconsistence rate, and obtain the fitting curve with R^2 of about 0.99. Finally, we estimated the reconstructed endpoint adjacency inconsistence rate of pre-WGD-1 ancestor being 5.70%. Therefore, the adjacency reliability for this ancestor is 94.0% (99.68%×100%×(1-5.7%)). So, pre-WGD-1 ancestor may have two endpoint adjacencies inconsistence ((1-94%)*36=2.16). All above optimization instances were solved with the *GUROBI* solver (http://www.gurobi.com) (v9.0.2).

Inferring gene orders in ancestors

We inferred the gene orders in post-WGD-1 ancestor based on pPG orders of block objects with matching ratio of 1:2 in *P. somniferum* and *P. setigerum* detected in section 9.1 and 9.2. And we inferred the gene orders in pre-WGD-1 ancestor based on pPG orders of all block objects in *P. somniferum* and *P. setigerum* detected in section 9.1. For each ancestor gene order inferring, we removed the species-specific and duplicated pPG in each block object, and built a directed weighted pPG graph for each syntenic block, where nodes, directed edge and weight represented pPGs, the downstream adjacency relations and support number, respectively. A topological sorting method with a greedy strategy was performed on each graph to find possible gene orders. The greedy strategy aiming to process the cycle based on the sum of weights of edges connecting sorted nodes and unsorted nodes. Finally, we obtained the ancestral pPG order of each block object. The post-WGD-1 ancestor was estimated as eleven protochromosomes with 27,355 genes, and the pre-WGD-1 ancestor was estimated as six protochromosomes with 19,816 inferred genes (**Fig. 2a**).

Downstream analysis

After ancestor genomes construction, we identified the chromosomal rearrangements in *Papaver* evolutionary history. From pre-WGD-1 ancestor to post-WGD-1 ancestor, we doubled pre-WGD-1 ancestor block sequences first and then used MO solving framework to get 1:1 matching relation. Then, the block adjacencies absent in pre-WGD-1 ancestor genome are chromosomal fusion events, and the block adjacencies absent in post-WGD-1 ancestor genome are chromosomal fission events. We found at least 11 chromosomal fissions and 12 chromosomal fusions compared between pre-WGD-1 ancestor and post-WGD-1 ancestor. Similarly, we counted the shuffling events from post-WGD-1 ancestor to *P. setigerum* genome and found at least 20 chromosomal fissions and 20 chromosomal fusions. We did not find any chromosomal fissions or fusions from post-WGD-1 ancestor to *P. somniferum* genome. To figure out the chromosomal shuffling events occurred in *P. rhoeas* evolution history, we considered the pre-WGD-1 ancestor as the most recent common ancestor of three *Papaver* species since time was close (7.2 Mya vs 7.7 Mya). By comparing *P. rhoeas* genome and pre-WGD-1 ancestor genome, at least five chromosomal fissions and four chromosomal fusions were detected (**Fig. 2a**).

Next, we tested whether the fissions in ancestor genomes and the fusions in modern genomes are randomly distributed. We randomly generated the same number of shuffling events based on a uniform distribution across whole genome 10,000 times to generate the background distribution of the shuffling events. We calculated mean (μ) and standard deviation (σ) of the background number of shuffling events on each chromosome or protochromosome, and then calculated the $z = \frac{N_{obv} - \mu}{\sigma}$, where N_{obv} is the observed number of shuffling events. *P*-value is calculated by z based on the standard normal distribution.

Finally, we performed Kyoto Encyclopedia of Genes and Genomes (KEGG) pathway enrichment of the genes around the breakpoint of these shuffling events by TBtools (v1.0692)[60] to explore the functions associated with the shuffling events (**Supplementary Fig. 22**). For each fusion breakpoint at the modern genomes, the genes were selected as ones between related block endpoints as well as 40 genes extending at each endpoint.

Supplementary Method 10. Detecting key genes of Benzylisoquinoline alkaloid (BIA) metabolism

We applied BlastP to identify BIA pathway related genes in *P. rhoeas*, *P. somniferum*, and *P. setigerum*. The genes related with morphinan biosynthesis pathways includes:

PSSDR1 (uniprot ID: I3PLR3), *PSCXE1* (uniprot ID: I3PLR2), *CYP82X1* (uniprot ID: I3V6B7), *CYP82X2* (uniprot ID: I3PLR0), *PSAT1* (uniprot ID: I3PLR4), *PSMT2* (uniprot ID: I3PLQ6), *CYP82Y1* (uniprot ID: I3PLR1), *PSMT3* (uniprot ID: I3PLQ7), *TMNT* (uniprot ID: Q108P1), *CYP719A21* (uniprot ID: I3QBP4), *PSMT1* (uniprot ID: I3V6A7), *STORR* (uniprot ID: P0DKI7), *SALSYN* (uniprot ID: B1NF18), *SALAT* (uniprot ID: Q94FT4, *SALR* (uniprot ID: Q071N0), *THS* (unipro ID: A0A2U9GHG9), *CODM* (unipro ID: D4N502), *T6ODM* (unipro ID: D4N500), and *COR* (unipro ID: Q9SQ7). The results summarized in **Supplementary Data 5**.

Supplementary Method 11. The evolution of BIA pathway in three *Papaver* species

We integrated the evidence of multiple sources from synteny, phylogeny and WGD to inferring the evolution of BIA gene cluster. We first dissected the impact of WGD and structural variants on the evolution of morphinan branch genes (*STORR, SALR, SALAT, SALSYN, THS, T6ODM, COR and CODM*) in the three *Papaver* species.

In three species, we found one copy of *STORR* and one copy of pre-fusion module in *P. somniferum*, two copies of *STORR* and pre-fusion modules in *P. setigerum*, while one copy of pre-fusion modules in *P. rhoeas* (**Fig. 3a, Supplementary Figs. 23-25**). We did not find any collinearity relations between *STORR* related regions and pre-fusion module related regions in neither *P. somniferum* nor *P. setigerum* (**Supplementary Figs. 12, 24**) indicating *STORR* formation was not a sole deletion event, ant may involve a translocation besides the proposed fusion event with unknown order, the so called ‘fusion, translocation’ (FT) event. We systematically examined the syntenic relations of the donor loci and recipient loci in three species (**Fig. 3a, Supplementary Fig. 25**), and found all four types of loci existed exactly once in *P. somniferum*, but twice in *P. setigerum*, while only the prior status of donor and recipient loci were observed once in *P. rhoeas* (**Fig. 3a, Supplementary Fig. 25**), confirming a translocation event involved in *STORR* formation.

Based on the WGD in three *Papaver* species, we proposed an evolutionary model to illustrate the birth of *STORR* at current BIA gene cluster (**Fig. 3b**). In the most recent common ancestor of the three species, only pre-fusion module presented at donor loci, which is preserved in *P. rhoeas*. After divergence from *P. rhoeas*, the ancestor of *P. somniferum* and *P. setigerum* underwent a WGD (WGD-1) at around 7.2 Mya resulting in two copies of pre-fusion modules at donor loci. Then, a FT event leading to the birth of *STORR* at recipient loci and resulting in one copy of pre-fusion modules at donor loci and one copy of *STORR* at recipient loci, which was inherited by *P. somniferum*. *P. setigerum* underwent a lineage specific WGD (WGD-2) after its divergence from *P. somniferum* giving rise to two copies of *STORR* at recipient loci and two copies of pre-fusion modules at donor loci (**Fig. 3b**). Moreover, we detected 17 types of DNA

transposons located at both donor loci and acceptor loci (**Supplementary Fig. 26**), suggesting transposable elements likely mediated the translocation event in the vicinity of *STORR*, although the exact mechanisms remain elusive.

We next investigated the evolution of genes encoding enzymes for catalyzing the subsequent steps of morphine biosynthesis after *STORR*. With a similar approach, we identified one copy of both *SALSYN* and *SALR* in *P. rhoeas*, four copies in *P. setigerum*, and two copies in *P. somniferum*, indicating the presence of *SALSYN* and *SALR* at the morphinan gene cluster in ancestor status of three species (**Fig. 4a, Supplementary Fig. 29**). We observed *THS* at both collinear copies in *P. somniferum*, therefore, most likely a single copy of *THS* at morphinan gene cluster loci was present before WGD-1 (**Fig. 4b, Supplementary Figs. 28, 29**). The fact that *P. setigerum* only has two copies of *THS* suggests one copy was lost after its divergence from *P. somniferum* but before WGD-2 (**Supplementary Figs. 28, 29**). Like *STORR*, one copy of *SALAT* in *P. somniferum*, two copies in *P. setigerum*, and none in *P. rhoeas*, indicates that *SALAT* was inserted at the morphinan gene cluster loci after WGD-1 but before the divergence of *P. setigerum* from *P. somniferum* (**Fig. 4, Supplementary Figs. 27, 29**). Moreover, we examined the *CODM*, *T6ODM*, and *COR* with a similar approach, and found the evolution of these genes was not affected by the WGD events, but more likely caused by lineage-specific local duplications (**Supplementary Data 4, Supplementary Fig. 42**).

As for the noscapine branch, we found four genes (*PSSDR1*, *CYP82X1*, *CYP719A21*, and *PSMT1*) have synteny copies in *P. setigerum* and *P. rhoeas* (**Fig. 4a**), indicating they were presented in the most recent common ancestor (MRCA) of the three *Papaver* species. For *PSAT1*, we did not find any syntenic pairs in three species. However, we found the best hit (BH) from BlastP results was *Pso04G13170.0* and six synteny copies of *Pso04G13170.0*, suggesting *Pso04G13170.0* was presented in the MRCA, and putatively duplicated as *PSAT1* after the divergence of *P. somniferum* from *P. setigerum* (**Supplementary Fig. 30**). For *PSCXE1*, we did not find any syntenic pairs in three species. However, we found the BH from BlastP results was *Pso04G00200.0* and one synteny copy of *Pso04G00200.0* in *P. setigerum* (*Pse16G13000.0*), suggesting *Pso04G00200.0* formation before the divergence of *P. somniferum* from *P. setigerum*, and then duplicated as *PSCXE1* by *P. somniferum* specific event (**Supplementary Fig. 31**). For *PSMT2*, we did not find any syntenic pairs in three species. We found the BH *Pso02G33600.0* with protein sequence identity as 58% from BlastP results. However, we did not find any nucleotide alignment between these two gene sequences by BlastN with e-value threshold as 1e-5 suggesting the origin of *PSMT2* was unclear (**Supplementary Fig. 32**). For *PSMT3*, we did not find any syntenic pairs in three species. However, we found the BH from BlastP results was *Pso05G43960.0* and six synteny copies of *Pso05G43960.0*, suggesting *Pso05G43960.0* was presented in the MRCA, and duplicated as *PSMT3* after the divergence of *P. somniferum* from *P. setigerum* (**Supplementary Fig. 33**). For *CYP82X2*, we did not find any syntenic pairs in three species. However, we found the reciprocal best hit (RBH) from BlastP results

was *CYP82X1*, suggesting a *P. somniferum* specific tandem duplication formed *CYP82X2* (**Supplementary Fig. 34**). For *CYP82Y1*, we found *STORR* P450 module was the best hit of with protein sequence identity of 60%. However, we did not find any nucleotide alignment between coding sequence of *CYP82Y1* and *STORR* P450 module by BlastN with e-value threshold as 1e-5 suggesting the origin of *CYP82Y1* was unclear (**Supplementary Fig. 35**). We also detected transposable elements around BIA gene cluster (**Supplementary Figs. 27-35**). However, about three fourth of *Papaver* genomes are repetitive elements and about half are TEs, making it difficult to associate specific TEs with the recruitment of individual BIA gene.

Alternatively, other explanations of the formation of the five genes (*SALAT*, *THS*, *PSAT1*, *PSCXE1*, and *PSMT3*) in BIA gene cluster based on old tandem duplications are also possible (**Supplementary Fig. 37**).

Supplementary Method 12. Gene tree construction

MEGA (v7.0)[61] was used to generate maximum likelihood phylogeny trees for *CYP82Y1*, *CYP82X2*, *PSCXE1*, *PSMT2*, *PSMT3*, *SALAT, PSAT1*, *STORR*, and *THS* located in BIA gene clusters with the JTT (Jones, Taylor, and Thorton) amino acid substitution model[62]. Statistical support for phylogenetic grouping was assessed by 100 bootstrap re-samplings.

Supplementary Method 13. Gene expression analysis

The RNA sequencing reads were subjected to quality control using FastQC (https://github.com/s-andrews/FastQC). Illumina sequencing adapters and poor-quality reads (quality score < 30) were trimmed using Trimommatic (v0.32)[63]. The cleaned high-quality RNA reads were used for *de novo* assembly of transcripts using Trinity (v2.1.1)[32], providing transcript evidence for genome annotations. To estimate the transcript abundance for annotated genes in three genomes, the cleaned RNA reads were aligned against reference genome using Hisat2 (v2.2.1)[64] and transcripts were discovered and quantified by Stringtie (v2.1.4)[65] and Ballgown[65] respectively with default parameters. We measured the gene expression level by TPM (Transcripts Per Million). The processed transcriptome data from different tissues in *P. rhoeas*, *P. somniferum* and *P. setigerum* were analyzed using in-house R scripts.

To compare the TPMs between different species, we normalized the TPM by calculating z-score in each species. Firstly, we calculated the mean and standard deviation values of all TPMs in each species. Then we calculated TPM_z of each TPM as $TPM_z = (TPM - \mu_{TPM}) / \sigma_{TPM}$, where μ_{TPM} is the mean value of all TPMs, and σ_{TPM} is the standard deviation of all TPMs. Furthermore, we calculated the

normalized TPM as $TPM_n = TPM_z + TPM_z^{\min}$ to make sure the normalized TPM non-negative, where $TPM_z^{\min}$ is the minimal value of all TPM_z.

Supplementary Method 14. Hi-C data analysis

Hi-C data alignment, filtering, and generation of Hi-C heatmap was conducted by Juicer software[66]. Raw Hi-C reads were aligned to the corresponding assembly by BWA (v0.7.17)[17] with Juicer default alignment parameters. Artifacts within Hi-C read pairs are filtered out by Juicer default filtering script. Hi-C matrixes are dumped by Juicer Tools Dump of 10k resolution. Tadtools (v0.76) was used for Hi-C interaction heatmap generation[67]. The chromatin loops were detected by HICCUPS[68] with parameter of '-r 5000,10000,25000'.

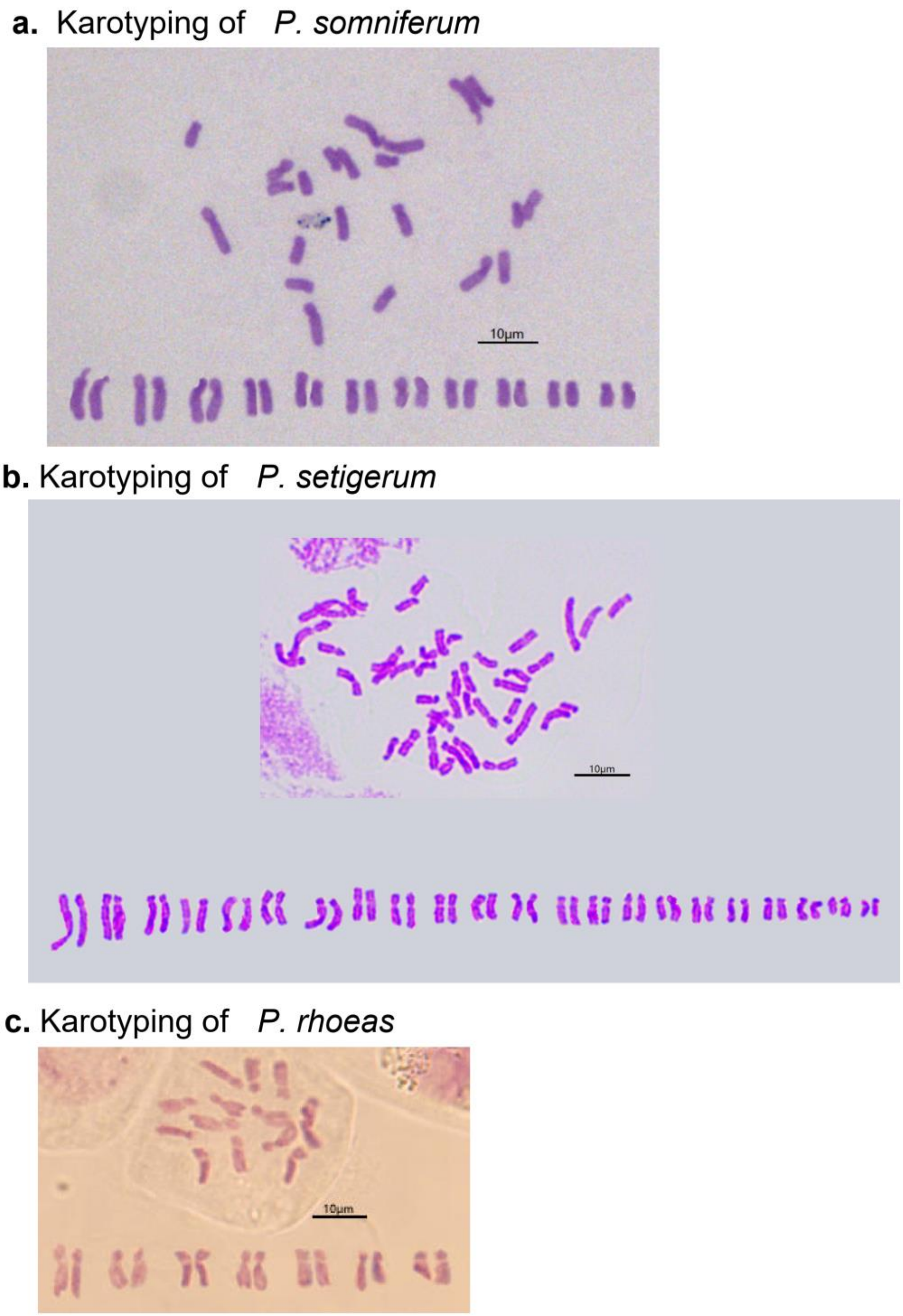

Supplementary Fig. 1. Karyotyping of *P. somniferum* (a)*,* *P. setigerum* (b)*, and P. rhoeas* (c)*.* For each karyotyping experiment, we repeated three times independently with the similar results.

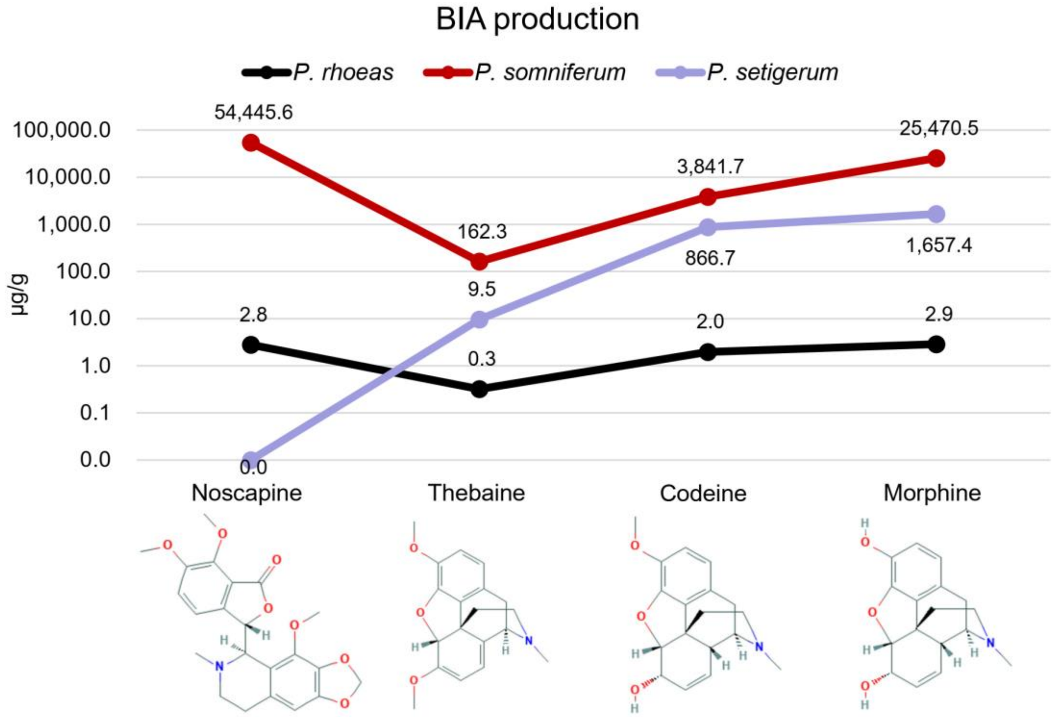

Supplementary Fig. 2. Quantification of four benzylisoquinoline alkaloids (BIA) (µg/g) of three *Papaver* species using HPLC-MS. The chemical structure of each BIA is from https://pubchem.ncbi.nlm.nih.gov.

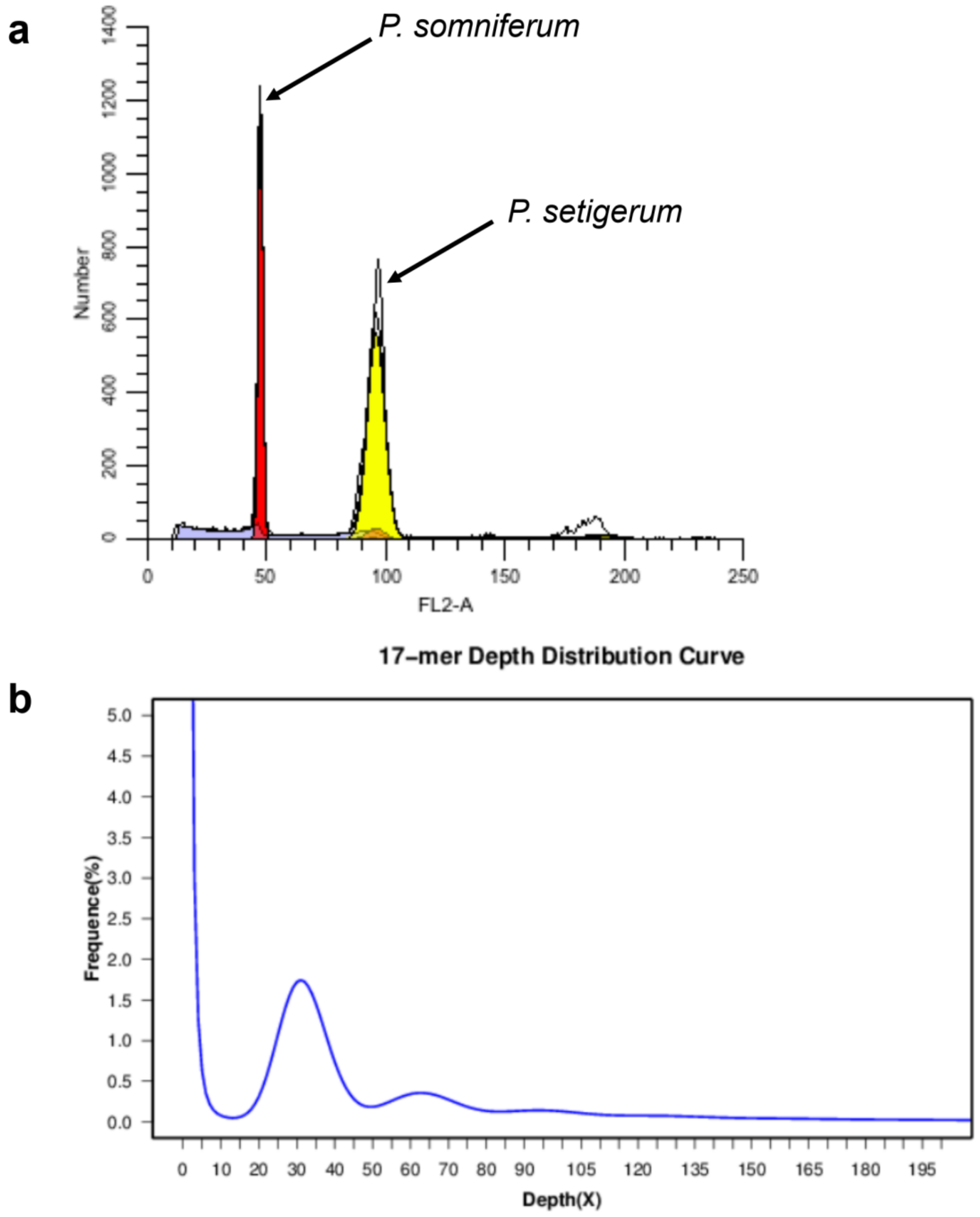

Supplementary Fig. 3. Genome survey of *P. setigerum*. **a**. Flow cytometry of *P. setigerum* nuclei using *P. somniferum* HN1 as reference. **b.** K-mer frequency distributions from base error corrected reads. With K=17, there is a frequency peak value at 32 which is used for genome size estimation.

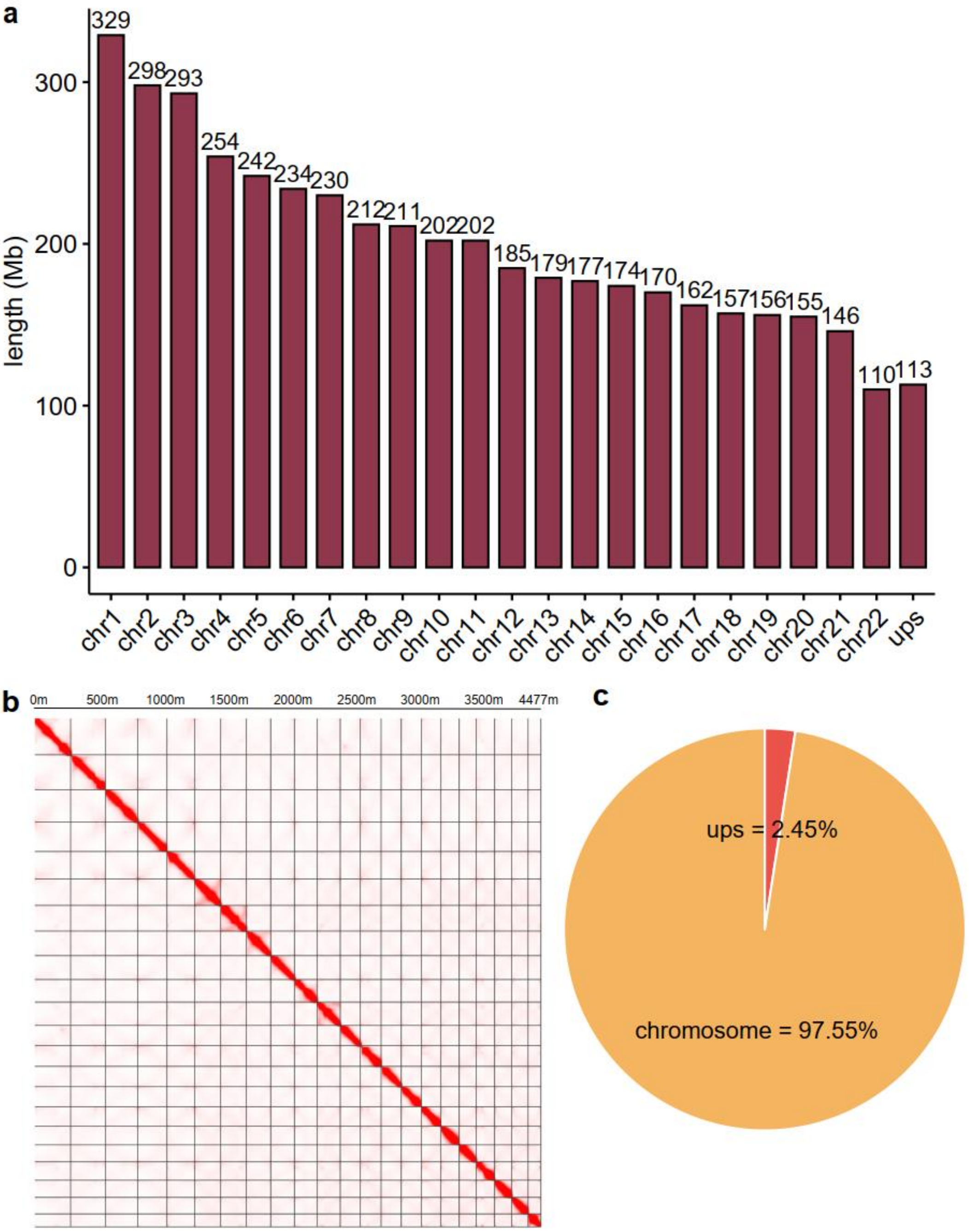

Supplementary Fig. 4. Summary of assembled genome of *P. setigerum*. a. the chromosome lengths of *P. setigerum*; **b.** Hi-C heatmap of *P. setigerum* is generated by juicebox[69]. **c.** The proportions of chromosomes and unplaced scaffolds for *P. setigerum.* ups: unplaced scaffolds. Source data underlying Supplementary Figure 4a is provided as a Source Data file.

a

b

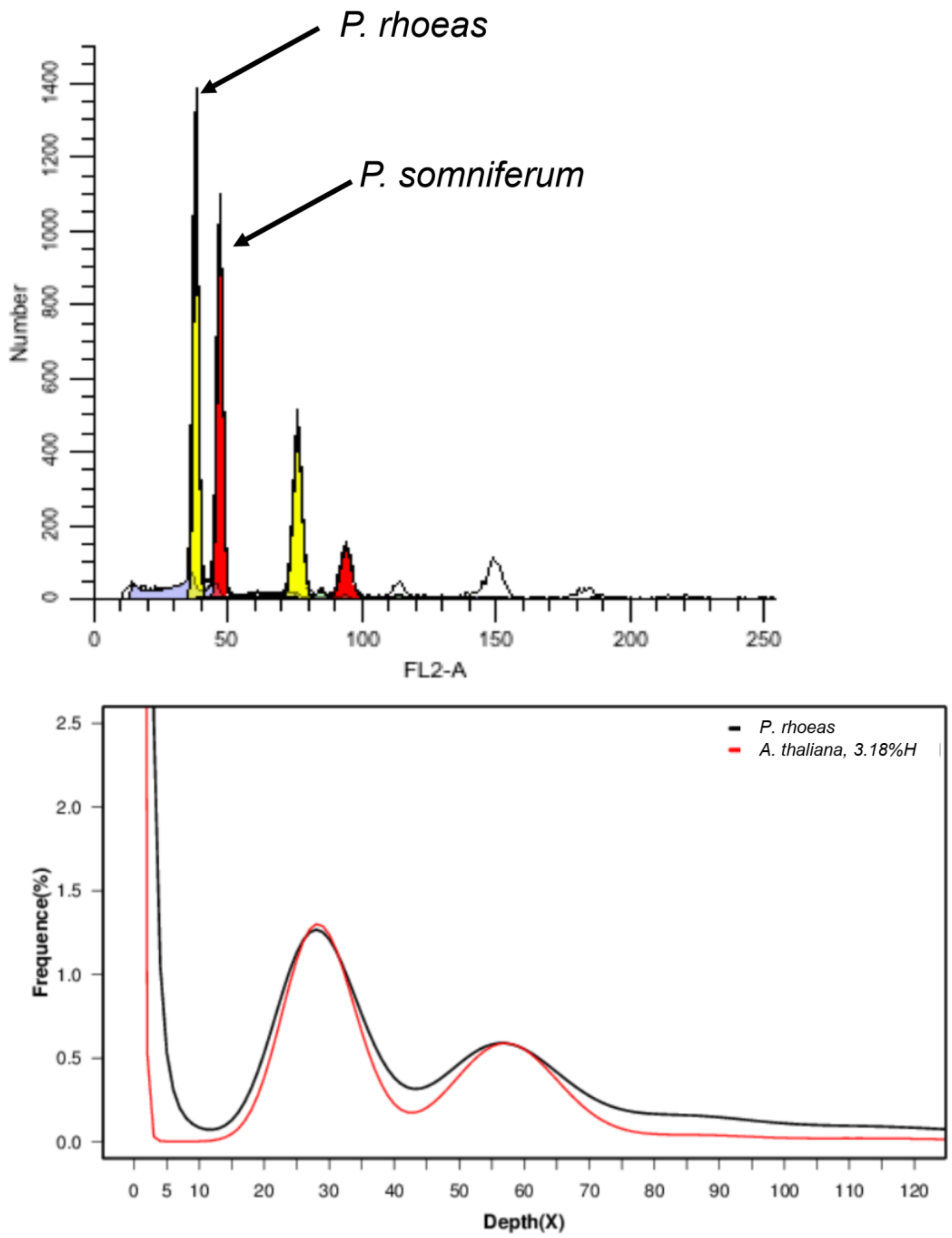

Supplementary Fig. 5. Genome survey of *P. rhoeas.* a. Flow cytometry of *P. rhoeas* nuclei using *P. somniferum* HN1 as reference. **b**. K-mer frequency distributions from base error corrected reads. With K=17, there is a major peak value at 28 and a minor peak at 55 indicating the high heterozygosity of P. rhoeas. The red line is K-mer frequency distribution of simulated 57X Illumina paired-end sequencing of Arabidopsis thaliana with 3.18% heterozygosity.

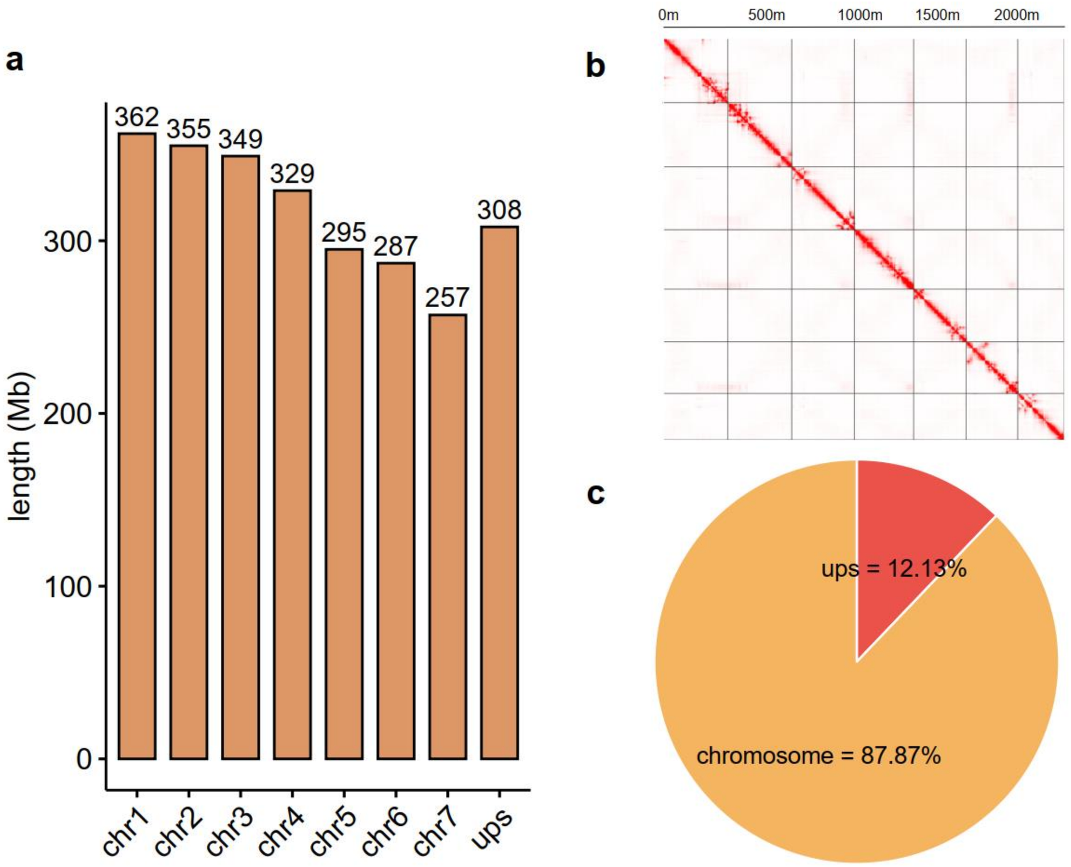

Supplementary Fig. 6. Summary of assembled genome of *P. rhoeas*. a. the chromosome lengths of *P. rhoeas*; **b.** Hi-C heatmap of *P. rhoeas* is generated by juicebox. **c.** The proportions of chromosomes and unplaced scaffolds for *P. rhoeas.* ups: unplaced scaffolds. Source data underlying Supplementary Figure 6a is provided as a Source Data file.

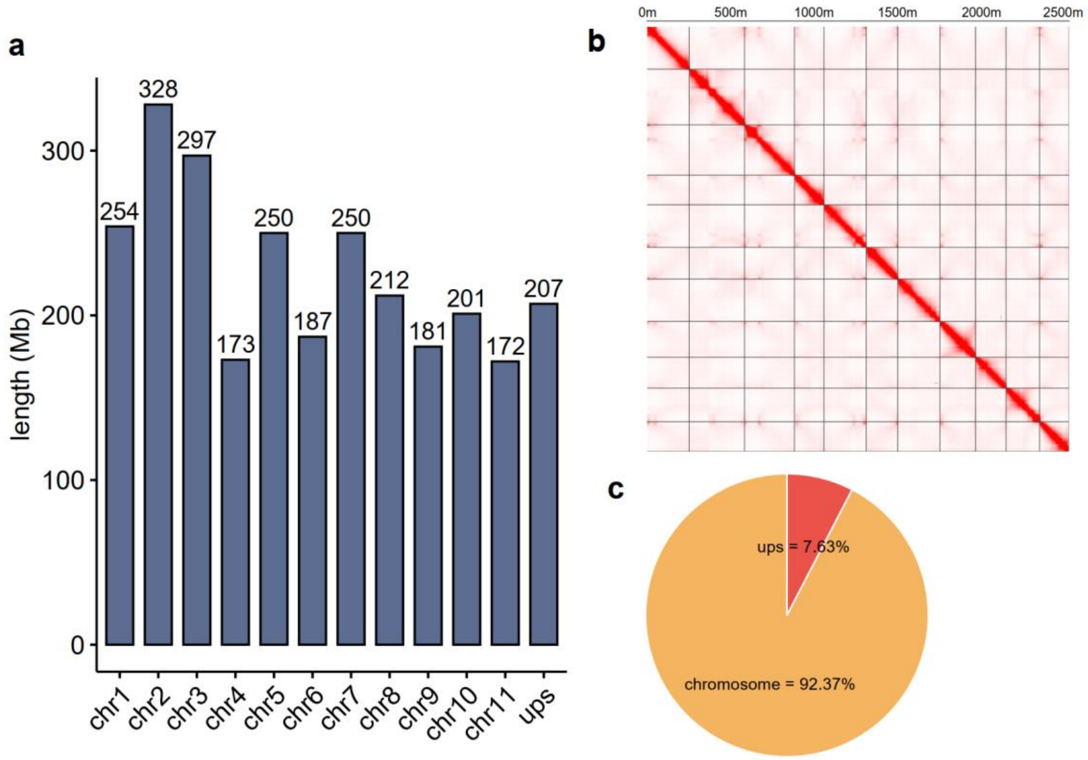

Supplementary Fig. 7. Summary of improved *P. somniferum* assembly. a. the chromosome lengths of *P. somniferum*; **b.** Hi-C heatmap of *P. somniferum* is generated by juicebox. **c.** The proportions of chromosomes and unplaced scaffolds for *P. somniferum.* ups: unplaced scaffolds. Source data underlying Supplementary Figure 7a is provided as a Source Data file.

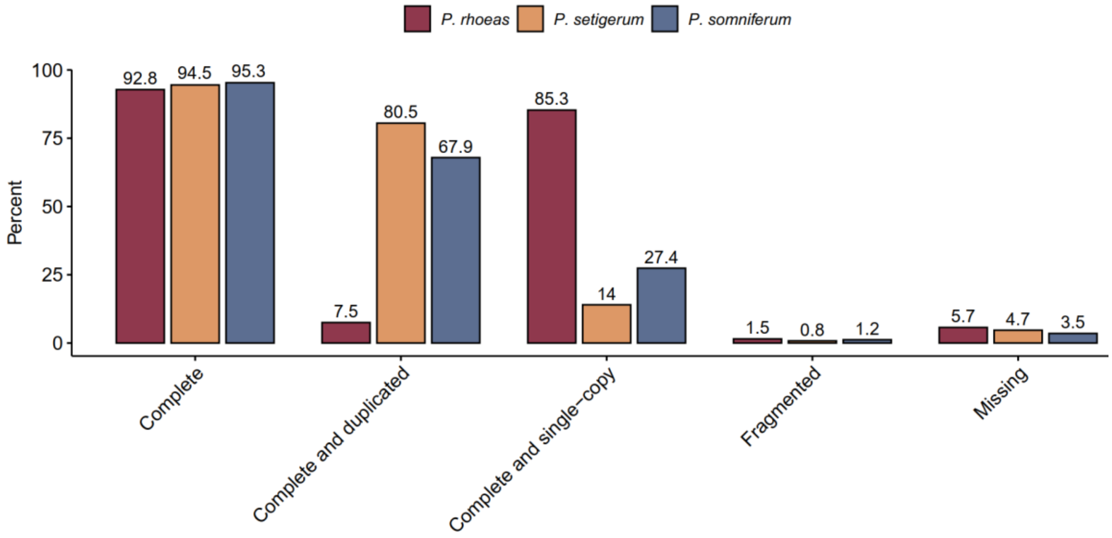

Supplementary Fig. 8. Benchmarking Universal Single-Copy Orthologs (BUSCO) evaluation of genome assembly of three species based on the plant early release version (v1.1b1, release May 2015) database, indicating the completeness of the genome assemblies are 95.3%, 94.5% and 92.8% for *P. somniferum*, *P. setigerum*, and *P. rhoeas*, respectively.

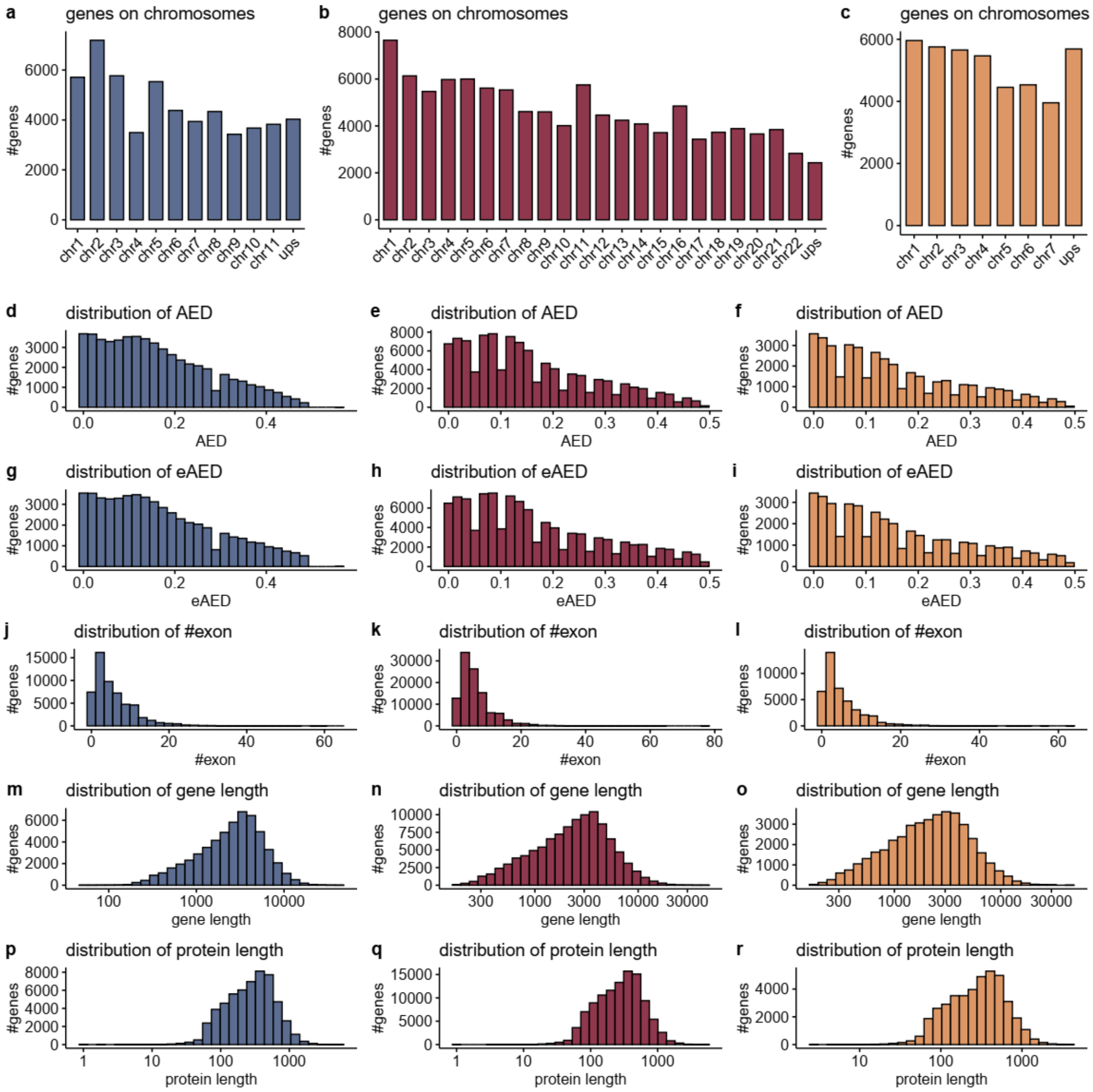

Supplementary Fig. 9. Statistics of protein-coding gene annotation of three species. The gene counts on different chromosomes and unplaced scaffolds (ups) for *P. somniferum* (**a**), *P. setigerum* (**b**), and *P. rhoeas* (**c**); The distribution of annotation evidence distance (AED) of *P. somniferum* (**d**), *P. setigerum* (**e**), and *P. rhoeas* (**f**); The distribution of exon annotation evidence distance (eAED) of *P. somniferum* (**g**), *P. setigerum* (**h**), and *P. rhoeas* (**i**); The distribution of exon numbers of *P. somniferum* (**j**), *P. setigerum* (**k**), and *P. rhoeas* (**l**); The gene length distribution of *P. somniferum* (**m**), *P. setigerum* (**n**), and *P. rhoeas* (**o**); The protein sequence length distribution of *P. somniferum* (**p**), *P. setigerum* (**q**), and *P. rhoeas* (**r**).

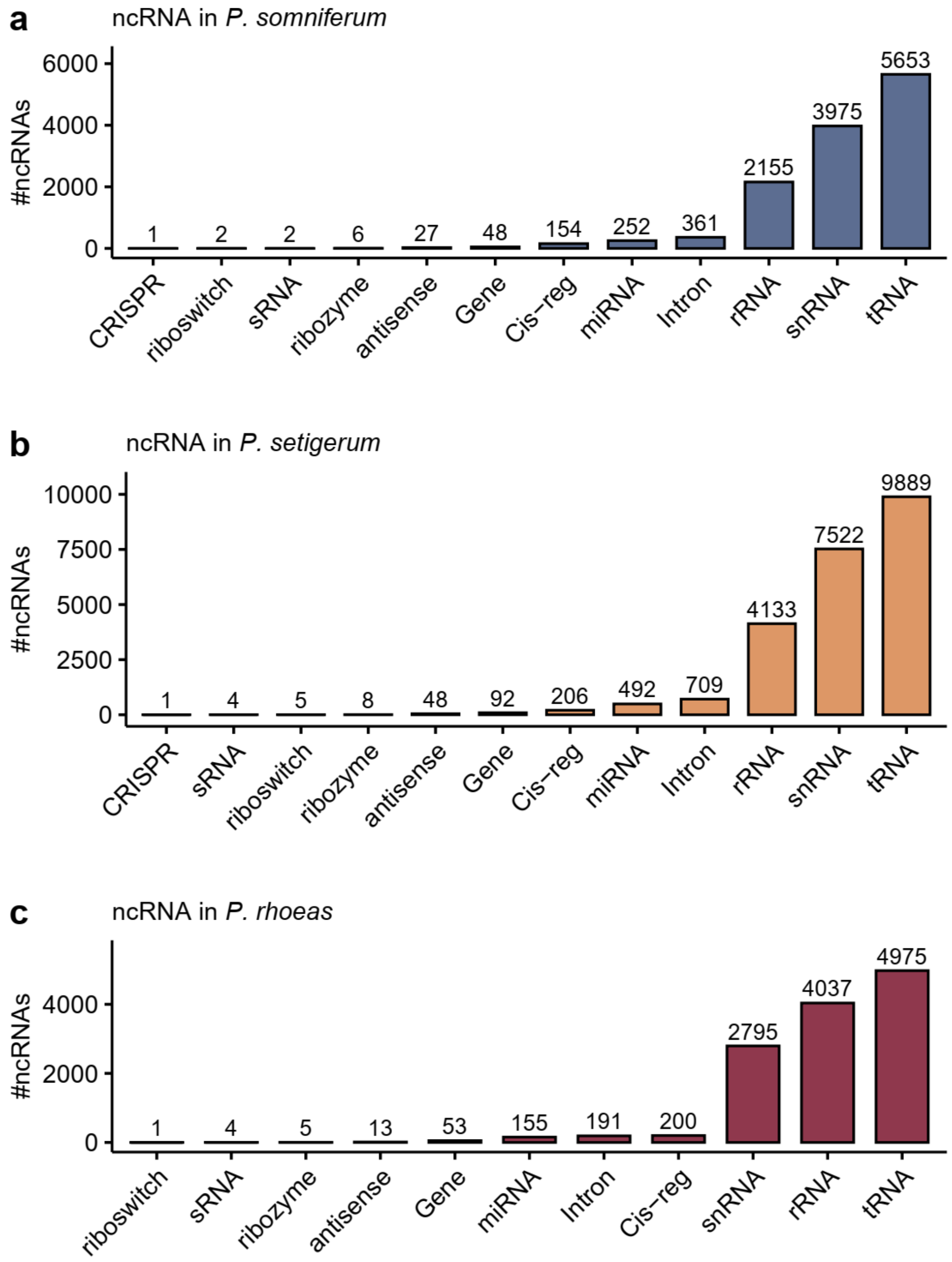

Supplementary Fig. 10. Summary of ncRNA annotation in *P. somniferum* (**a**), *P. setigerum* (**b**), and *P. rhoeas* (**c**).

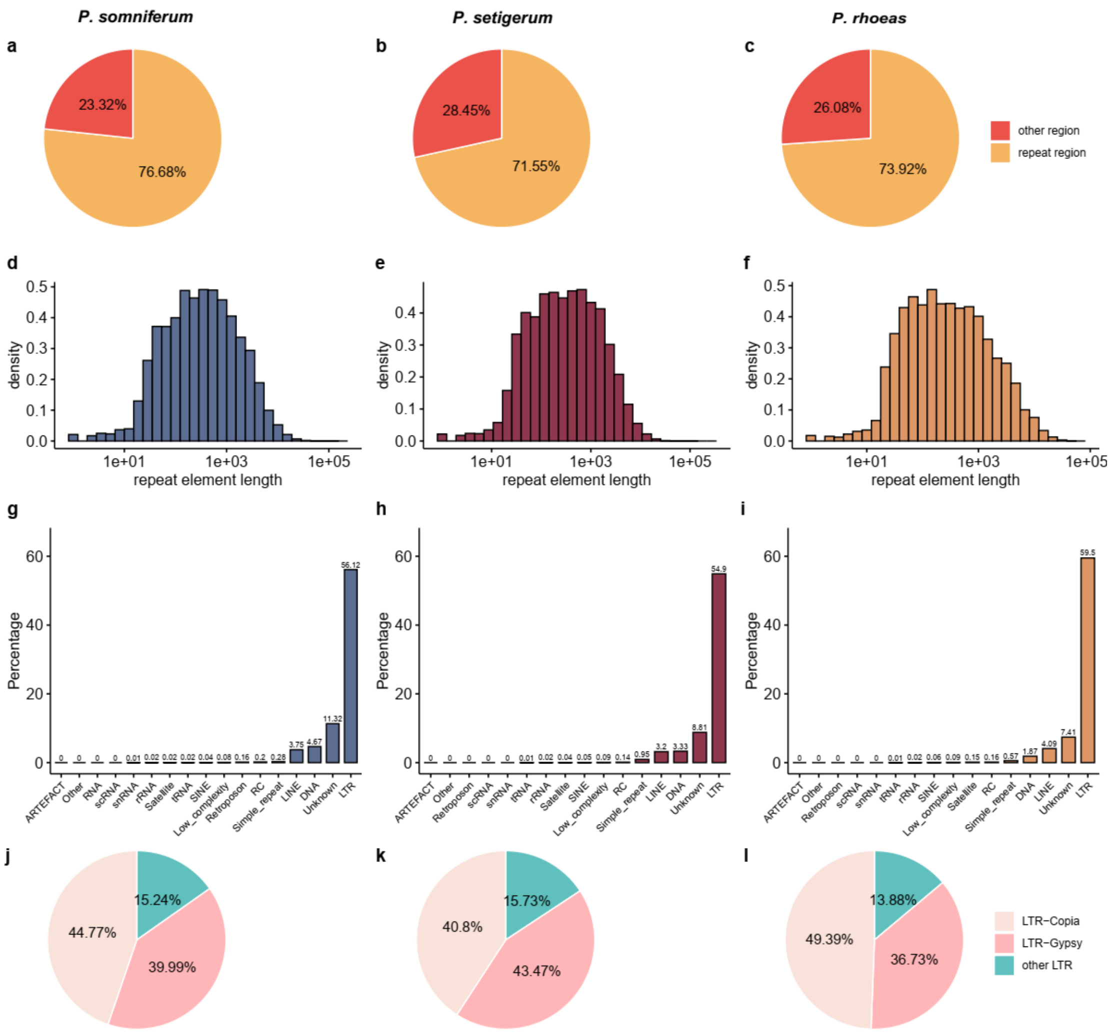

Supplementary Fig. 11. Summary of repetitive element annotations. The proportions of repetitive elements to genome of *P. somniferum* (**a**), *P. setigerum* (**b**) and *P. rhoeas* (**c**); The length distribution of the repetitive elements in *P. somniferum* (**d**), *P. setigerum* (**e**) and *P. rhoeas* (**f**); The proportions of different classes of repetitive elements in *P. somniferum* (**g**), *P. setigerum* (**h**) and *P. rhoeas* (**i**), the LTR (long terminal repeats) are the most abundant repetitive elements in three species; The proportions of different LTR species in *P. somniferum* (**j**), *P. setigerum* (**k**) and *P. rhoeas* (**l**).

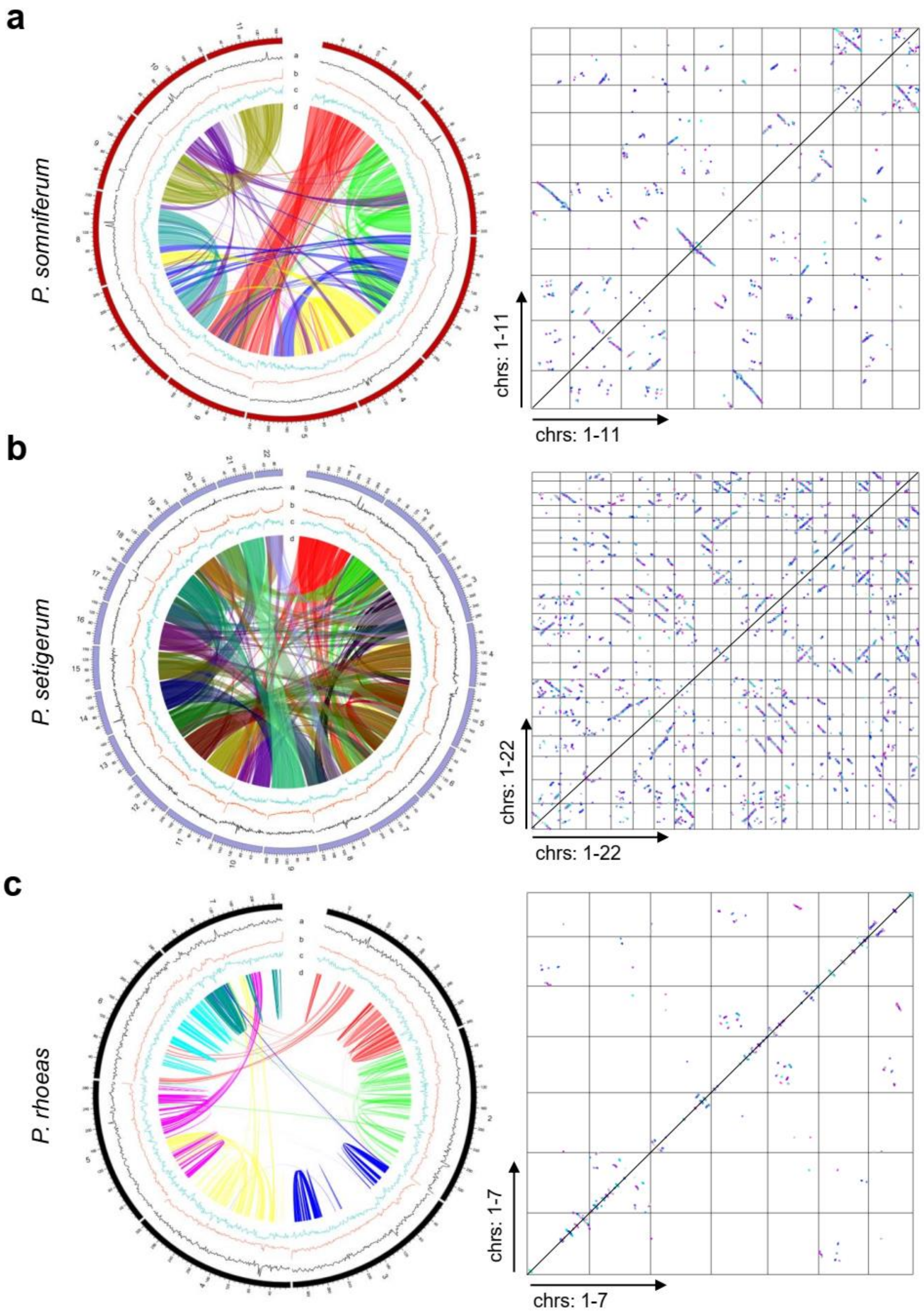

Supplementary Fig. 12. Synteny analysis of *P. somniferum* (**a**), *P. setigerum* (**b**), and *P. rhoeas* (**c**). The left and right panels are the circus plots and dotplots to show the duplication events in three genomes, such as one whole genome duplication (WGD) event in *P. somniferum*, two WGD events in *P. setigerum*, and segmental duplications in *P. rhoeas*. In the circus plots, the tracks a, b, and c represent the distribution of gene density, repeat density, and GC density, respectively (calculating in 2-Mb windows). Track d shows syntenic blocks. Band width is proportional to syntenic block size. Different colors in dotplots indicate different synteny blocks automatically generated by MCScanX. Source data are provided as a Source Data file.

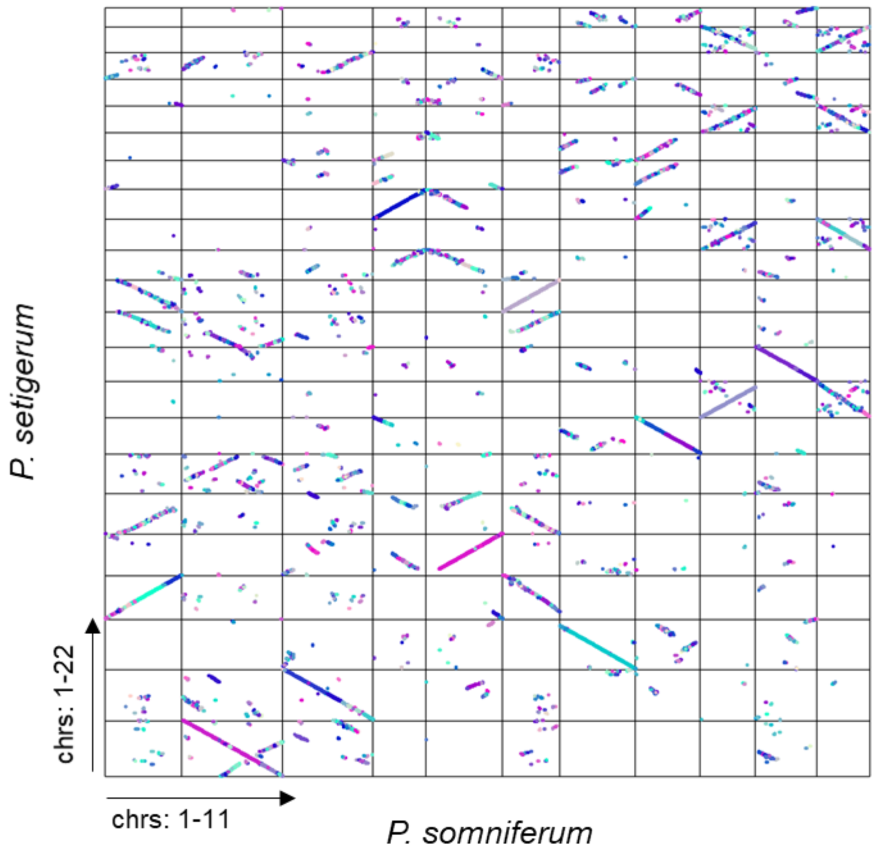

Supplementary Fig. 13. Synteny dotplot between *P. somniferum* (the *x*-axis) and *P. setigerum* (the *y*-axis), indicating the 2:4 syntenic relationship between *P. somniferum* and *P. setigerum*. Different colors indicate different synteny blocks automatically generated by MCScanX. Source data is provided as a Source Data file.

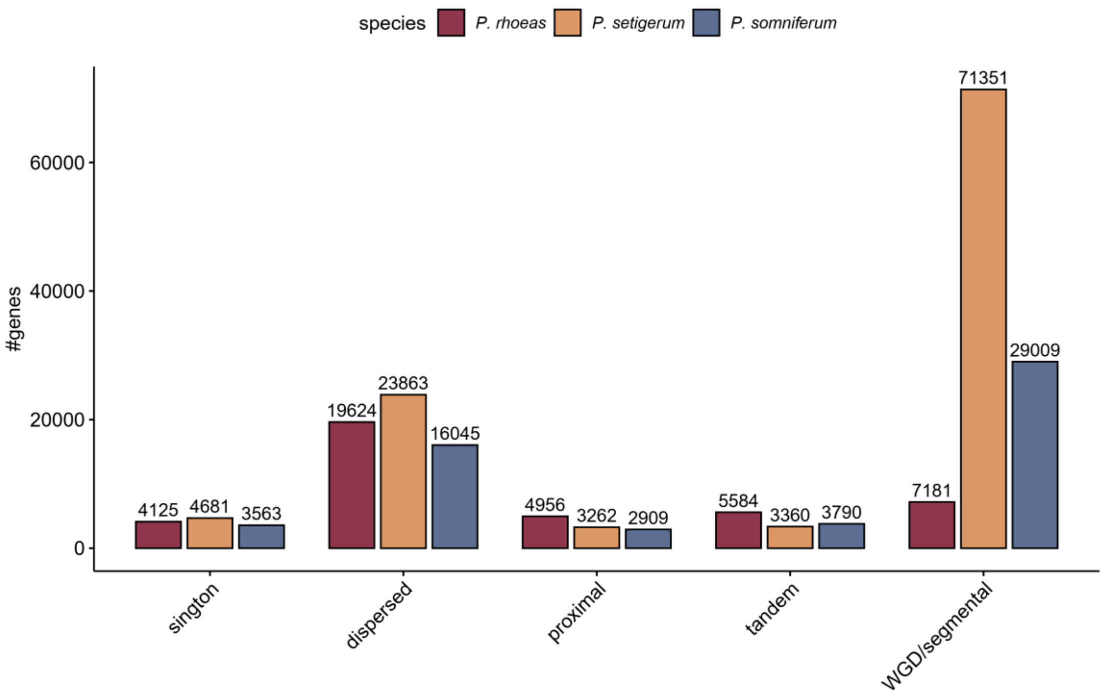

Supplementary Fig. 14. Summary of genes with different duplication types in three *Papaver* genomes. The duplication types are detected by MCScanX, and the definitions are: Singleton: no duplication; WGD/segmental: whole genome or segmental duplications (collinear genes in collinear blocks); Tandem: consecutive duplication; Proximal: duplications in nearby chromosomal region but not adjacent; Dispersed: duplications of modes other than tandem, proximal, or WGD/segmental. WGD: whole genome duplication. Source data is provided as a Source Data file.

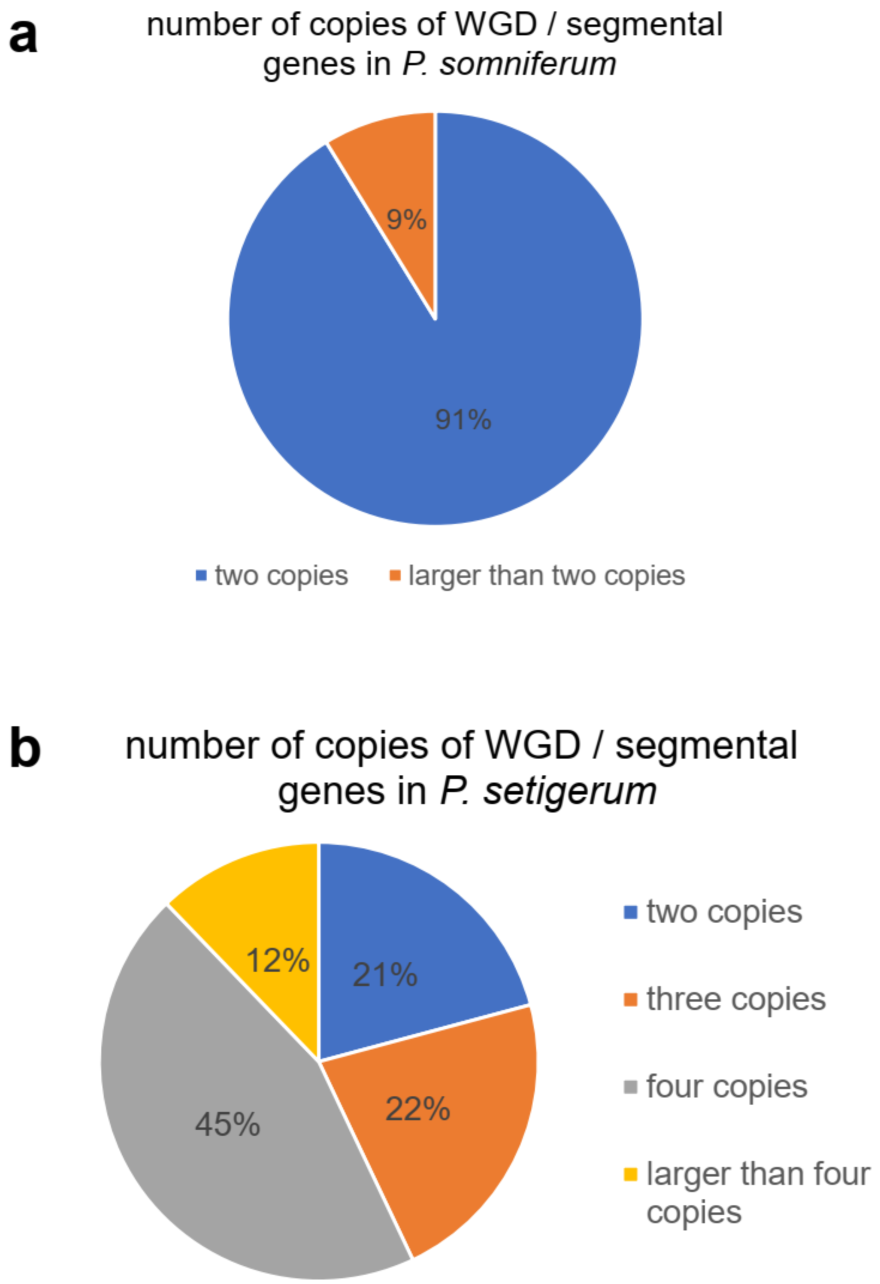

Supplementary Fig. 15. Distribution of copy numbers of genes resulting from WGD / segmental duplications in *P. somniferum* (a) and *P. setigerum* (b). WGD: whole genome duplication. Source data is provided as a Source Data file.

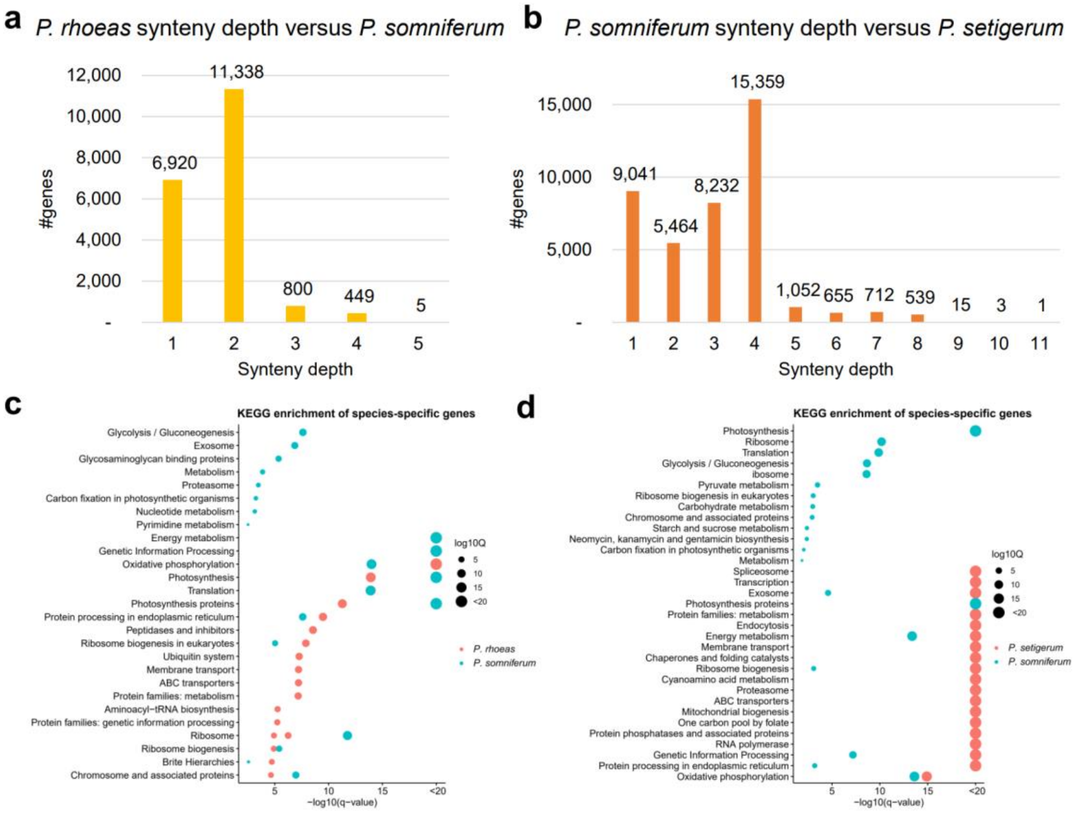

Supplementary Fig. 16. The synteny depth of *P. rhoeas* versus *P. somniferum* (**a**) and *P. somniferum* versus *P. setigerum* (**b**). **c.** The pathway enrichment of *P. rhoeas* specific genes and *P. somniferum* specific genes based on the comparison between *P. rhoeas* and *P. somniferum*. **d.** The pathway enrichment of *P. setigerum* specific genes and *P. somniferum* specific genes based on the comparison between *P. setigerum* and *P. somniferum*. We selected the top20 significantly enriched pathways in this figure. Source data is provided as a Source Data file.

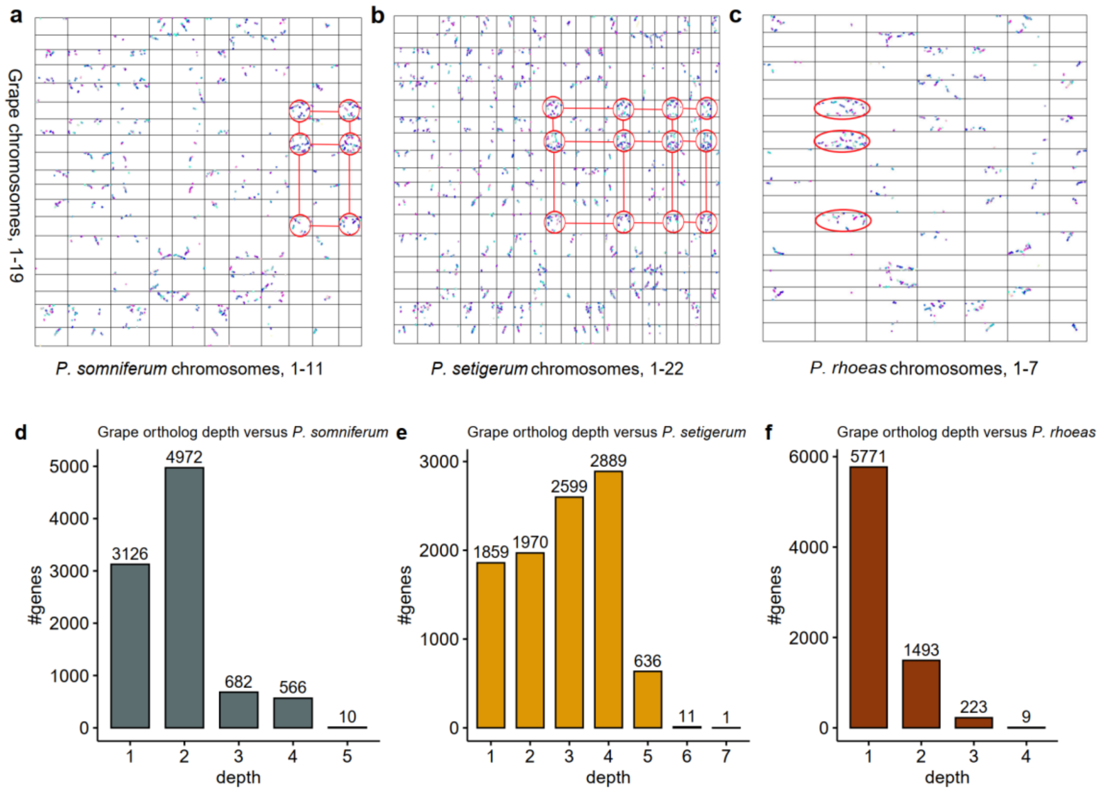

Supplementary Fig. 17. Synteny analysis between three *Papaver* genomes with grape genome. a-c. Dotplot to show the synteny between grape and three *Papaver* genomes; **d-f.** Ortholog depth density plots showing the number of *Papaver* orthologs per grape gene. Each dot in the dotplot indicates a syntenic gene pair detected by MCScanX. Different color indicates different synteny block. Source data underlying Supplementary Figure 17a-c are provided as a Source Data file.

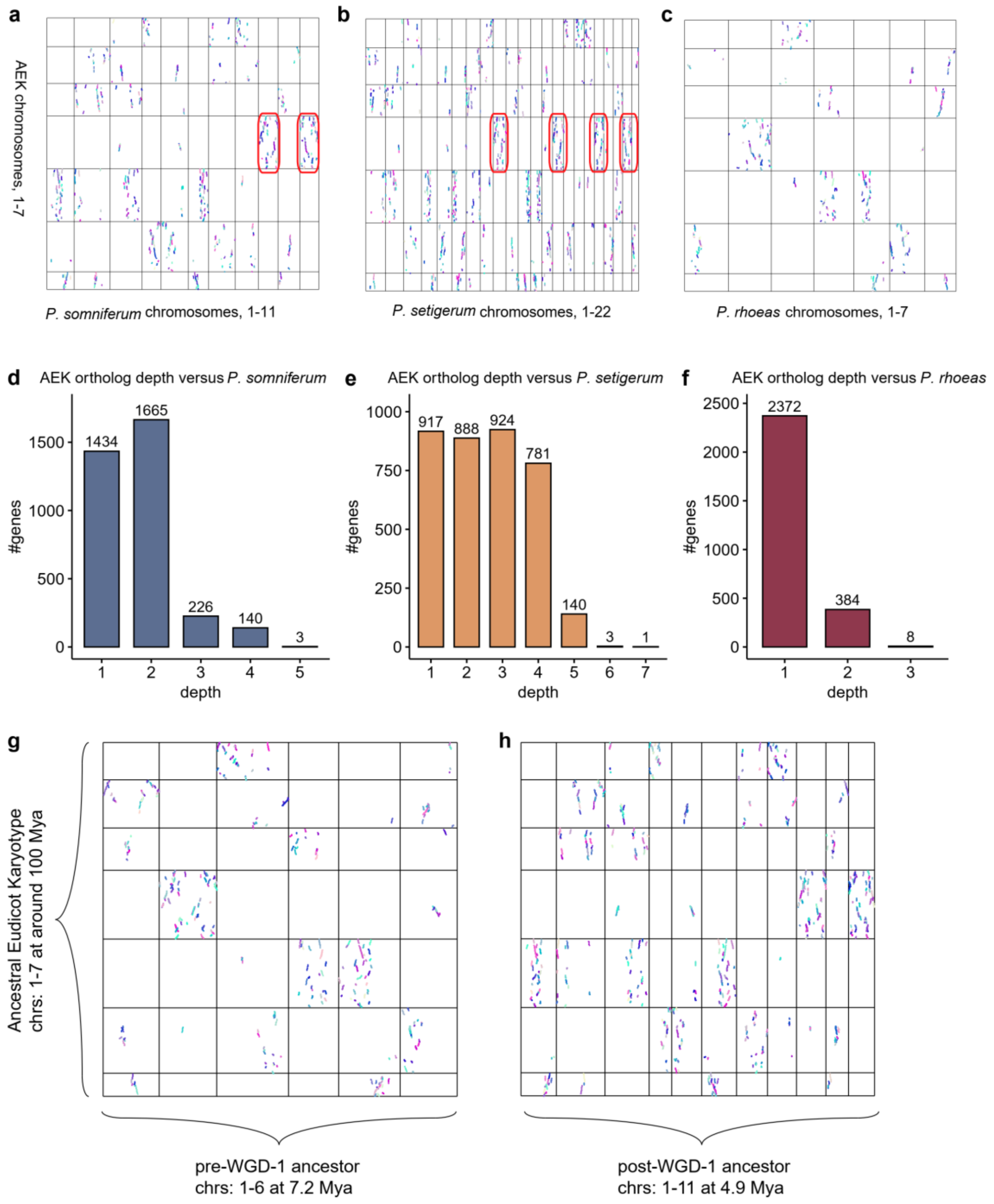

Supplementary Fig. 18. Synteny analysis between three *Papaver* genomes with Ancestral Eudicot Karyotype (AEK) genome. a-c. Dotplot to show the synteny results between AEK and three *Papaver* genomes **d-f.** Ortholog depth density plot showing the number of *Papaver* orthologs per AEK gene. **g.** Dotplot between pre-WGD-1 ancestor (the x-axis) and AEK (the y-axis). **h**. Dotplot between post-WGD-1 ancestor (the x-axis) and AEK (the y-axis). Each dot in dotplots indicates a syntenic gene pair detected by MCScanX. Different color indicates different synteny block. Mya: million years ago. Source data underlying Supplementary Figure 18a-c, 18g, and 18h are provided as a Source Data file.

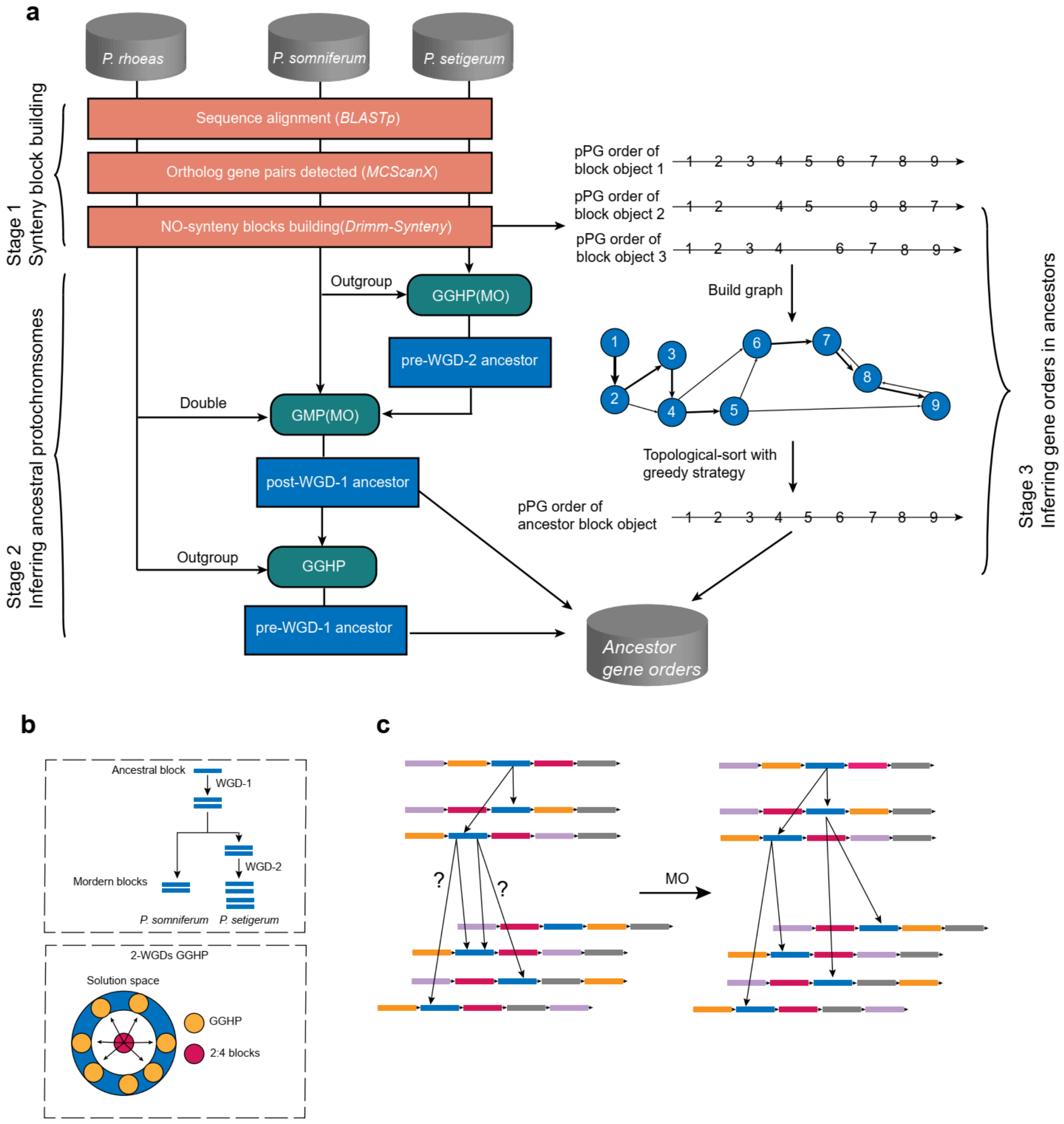

Supplementary Fig. 19. Ancestral genome reconstruction. a. Workflow for reconstruction of ancestor genomes of three *Papaver* species, including synteny block building, inferring ancestral protochromosomes and inferring gene orders in ancestor. NO: non-overlapping. GMP: Genome median problem. GGHP: Guided genome halving problem. MO: Matching optimization. pPGs: putative protogenes which are ortholog gene groups. The grey cylinders are genome annotation data. The orange rectangles are three steps in stage 1. The dark green rounded rectangles are computational models. The blue rectangles are ancestral syntenic block sequences for the corresponding computational models. The arrow lines with numbers are pPG order for each block copy (block object). **b.** A cartoon to simulate the block evolution in *P. somniferum* and *P. setigerum* (top). The ancestral block was duplicated after the first whole genome duplication (WGD-1) and the second WGD (WGD-2), resulting in two modern copies in *P. somniferum* and four modern copies in *P. setigerum*. In this simulation, we only consider the conserved blocks without losing. The blue rectangles

represent synteny blocks. Inferring the ancestral block based on 2:4 modern blocks is a 2-WGDs guided genome halving problem (GGHP) (bottom). In this problem, different modern block matching will obtain different GGHP solution. The annular represents computational solution space for 2:4 synteny block (*P. somniferum* and *P. setigerum*). Each orange circle represents a GGHP solution. **c**. Matching optimization (MO) strategy solves the 2-WGDs GGHP and obtain the optimized solution. MO can help us find block matching relation between *P. somniferum* and *P. setigerum* based on minimized genomic distance. Then we can transform complex problem into traditional GMP and GGHP. The different color of rectangles and arrows represent the order and direction of each block.

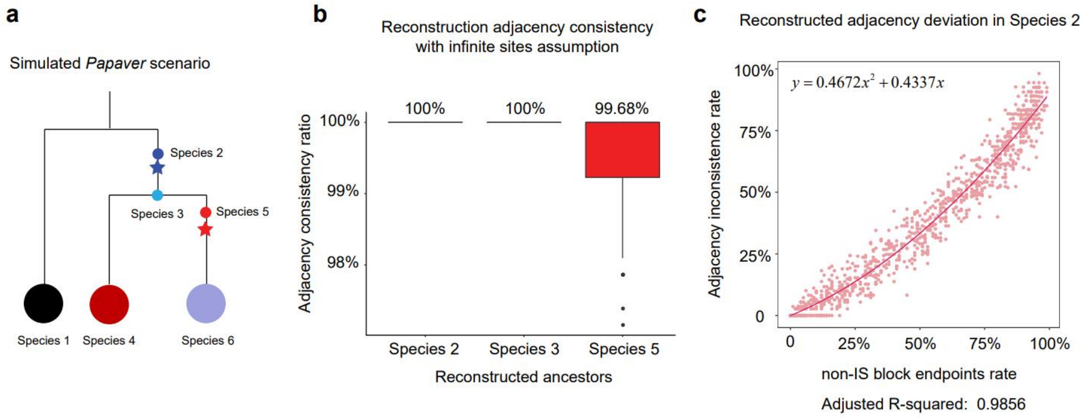

Supplementary Fig. 20. Evaluation for reconstructed ancestral protochromosomes in simulated *Papaver* scenario. a. Simulated *Papaver* evolutionary scenario. The stars are whole genome duplication (WGD) events. The small points indicated the ancestors. The big circles are species in evolution trees. **b.** Reconstructed adjacency consistency with infinite sites assumption for 200 repeat tests. Reconstructed Species 2 and Species 3 (represent pre- and post-WGD-1 ancestors) can be correctly reconstructed in 200 times. Reconstructed Species 5 (pre-WGD-2 ancestor) can be reconstructed with average 99.68% block adjacency consistency compared with simulated result in 200 times. **c.** Quadratic polynomial fitting the relationship between non-IS block endpoints rate and adjacency inconsistence rate for reconstructed Species 2 in non-infinite sites simulation. Source data are provided as a Source Data file.

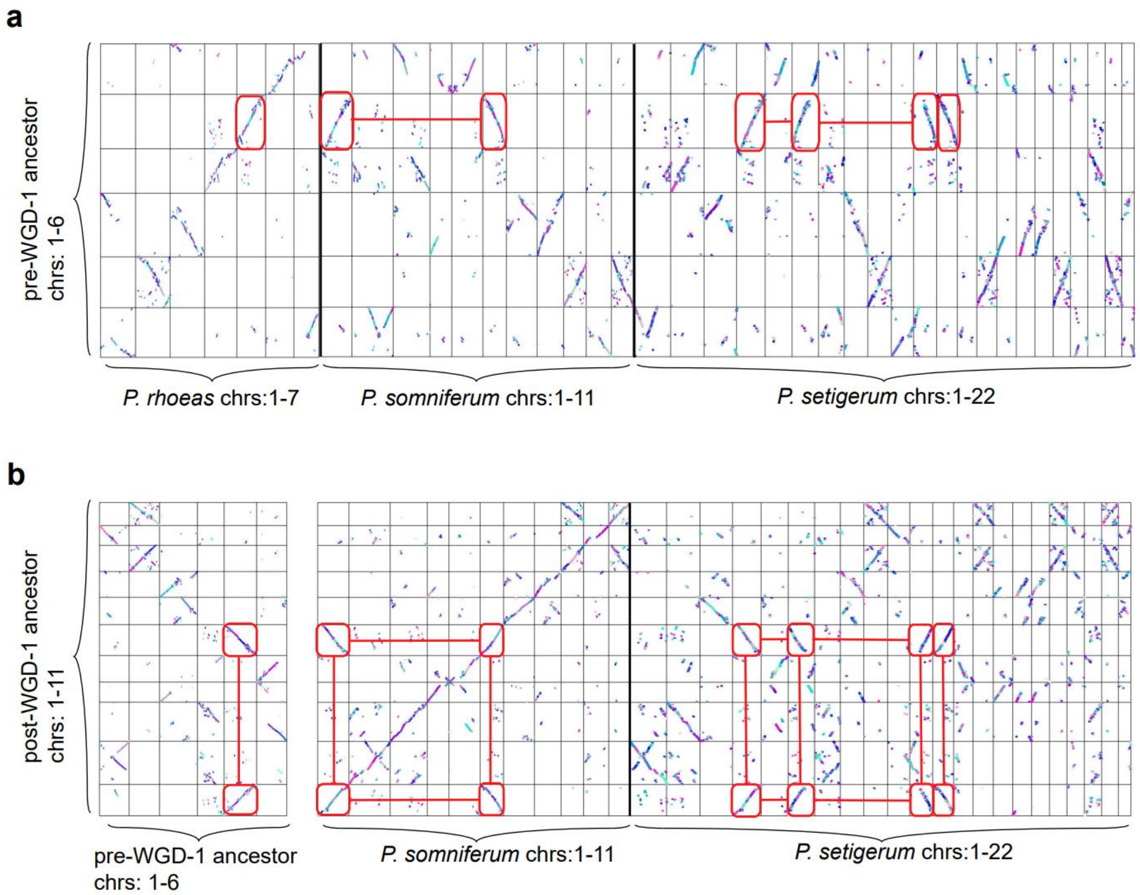

Supplementary Fig. 21. Dotplot between ancestors with three species genomes. a. Dotplot between pre-WGD-1 ancestor and *P. somniferum*, *P. setigerum*, and *P. rhoeas*. **b**. Dotplot between post-WGD-1 ancestor and *P. somniferum*, *P. setigerum*, and pre-WGD-1 ancestor. Comparisons between three *Papaver* genomes and two reconstructed ancestors (pre-WGD-1 ancestor and post-WGD-1 ancestor) reveal the differences between the ancestor genomes and the modern *Papaver* genomes. Different colors in dotplots indicate different synteny blocks automatically generated by MCScanX. Source data are provided as a Source Data file.

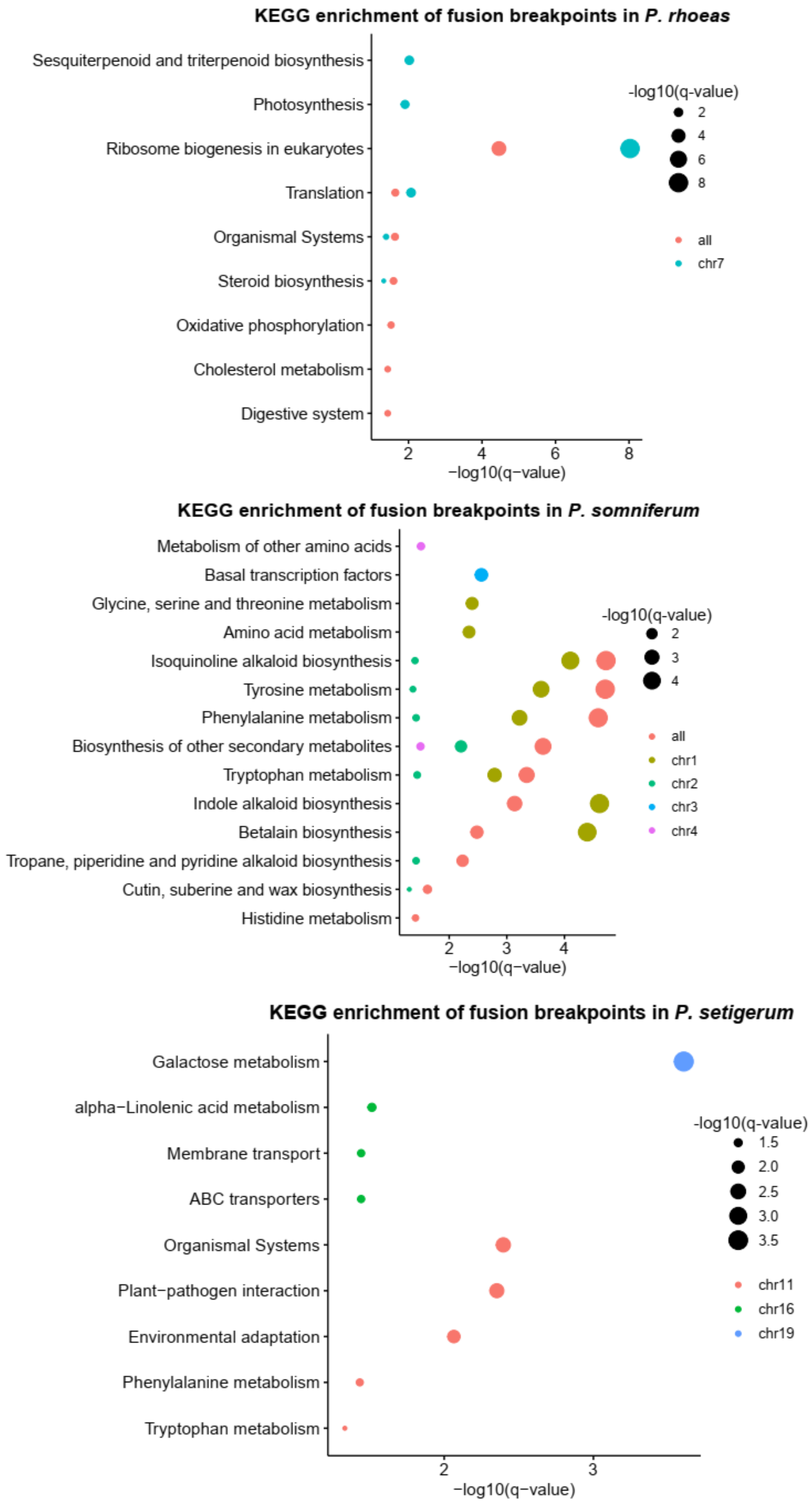

Supplementary Fig. 22. KEGG pathway enrichment of fusion breakpoints related genes in three *Papaver* species. all: genes around all fusion breakpoints; different chromosome means used fusion breakpoints related genes at the corresponding chromosome; unlisted chromosomes mean no fusion breakpoints or no significantly enriched pathway. KEGG: Kyoto Encyclopedia of Genes and Genomes. Source data are provided as a Source Data file.

a

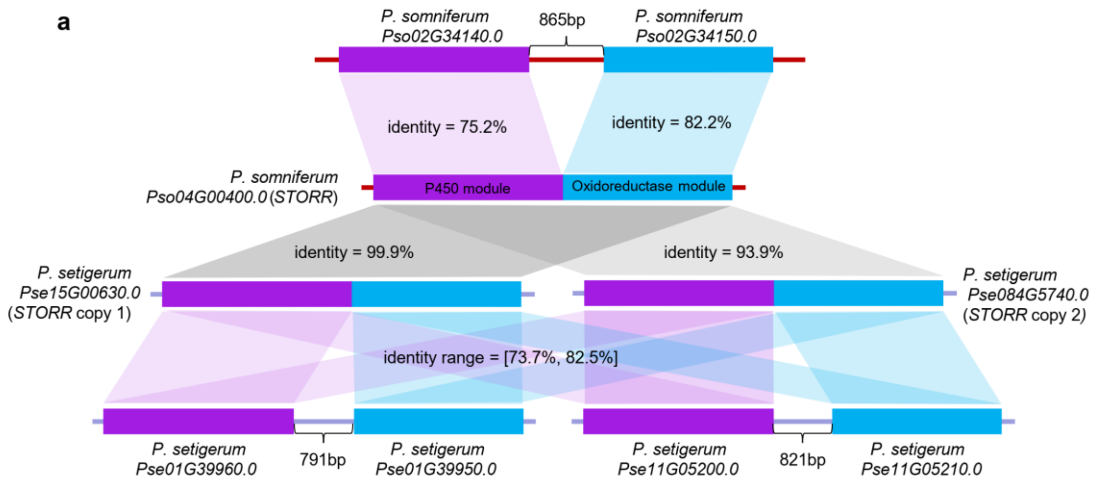

b

Gene_1	Gene_2	Identity	Gene_1	Gene_2	Identity	Gene_1	Gene_2	Identity
Pso04G00400.0 (*P. somniferum, STORR*)	Pso02G34140.0	75.2%	Pse15G00630.0 (*P. setigerum, STORR* copy 1)	Pso02G34140.0	75.2%	Pse08G45740.0 (*P. setigerum, STORR* copy 2)	Pso02G34140.0	75.2%
	Pso02G34150.0	82.2%		Pso02G34150.0	82.2%		Pso02G34150.0	76.6%
	Pse01G39950.0	82.2%		Pse01G39950.0	82.2%		Pse013G9950.0	76.6%
	Pse01G39960.0	75.4%		Pse01G39960.0	75.4%		Pse01G39960.0	75.4%
	Pse11G05200.0	73.9%		Pse11G05200.0	73.9%		Pse11G05200.0	73.7%
	Pse11G05210.0	82.5%		Pse11G05210.0	82.5%		Pse11G05210.0	76.9%
	Prh07G32030.0	75.2%		Prh07G32030.0	77.8%		Prh07G32030.0	78.0%
	Prh07G32040.0	79.1%		Prh07G32040.0	79.1%		Prh07G32040.0	69.6%

Supplementary Fig. 23. Alignment of *STORR* with pre-fusion genes in *P. setigerum* and *P. somniferum*. a. Sequence identity between *STORR* copies in *P. somniferum* and *P. setigerum* and the relations between *STORR* and its ancestor corresponding to the P450 and oxidoreductase modules in *P. setigerum and P. somniferum*. **b**. The protein sequence identity of each *STORR* to each pre-fusion gene calculated by BlastP.

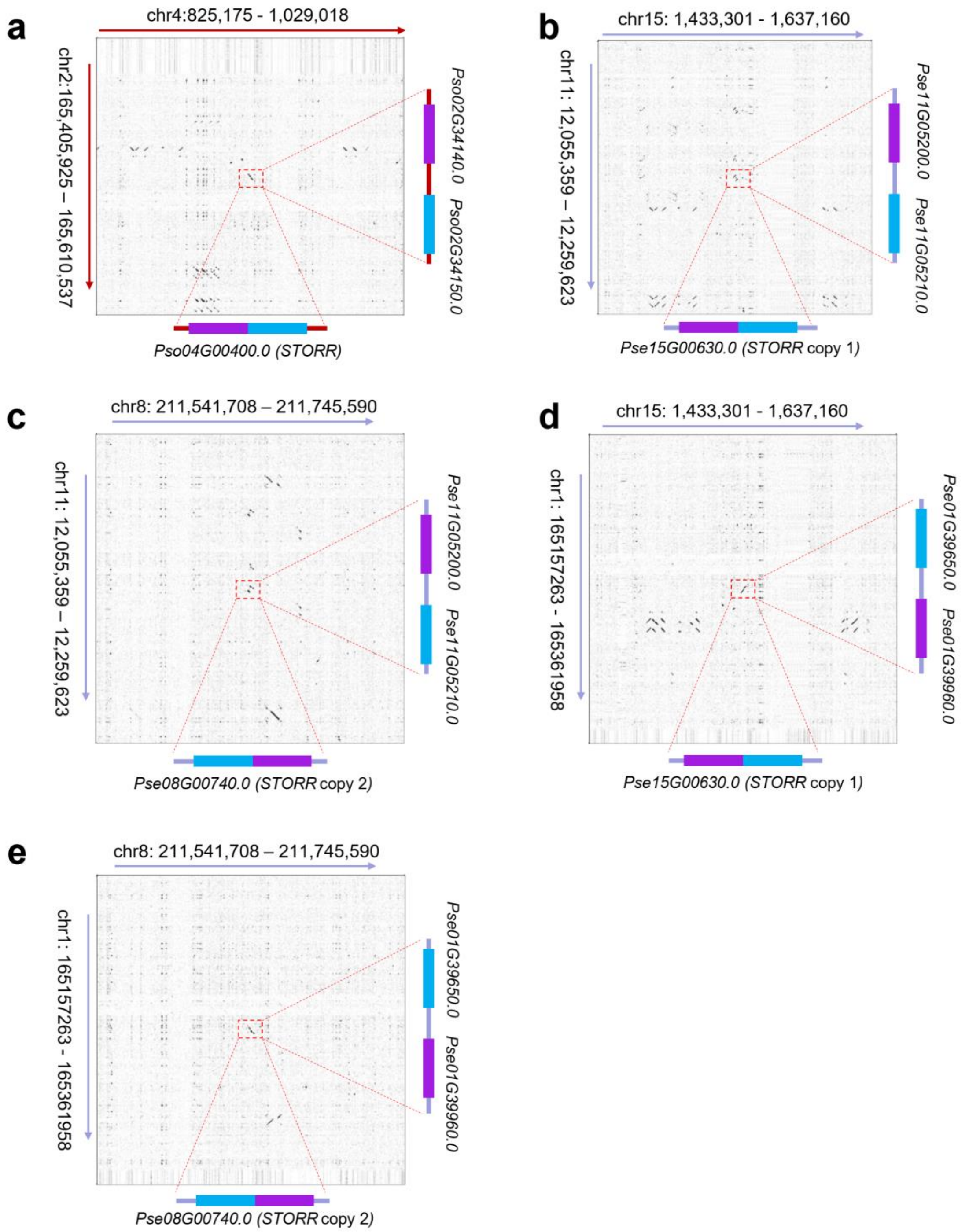

Supplementary Fig. 24. Dotplots of sequence alignment showing the lack of synteny relations between *STORR* locus and the pre-fusion locus in *P. somniferum* (**a**) and *P. setigerum* (**b-e**).

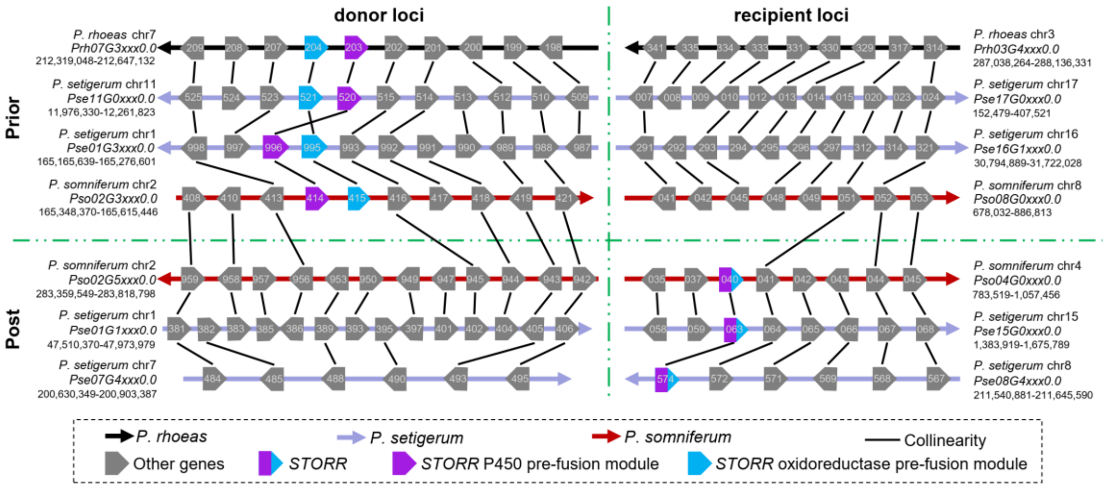

Supplementary Fig. 25. The synteny relations of *STORR* donor and recipient loci. The genomic evidences of the 'fusion, translocation' event leading to *STORR* formation at morphinan gene cluster. The syntenic relations of genes in the donor loci and the recipient loci with both prior and post statues of 'fusion, translocation' event were illustrated in three *Papaver* species. The directions of arrow indicated the chromosome from 5' to 3', and the last few digits of the gene ID were labeled on the open reading frames. Source data are provided as a Source Data file.

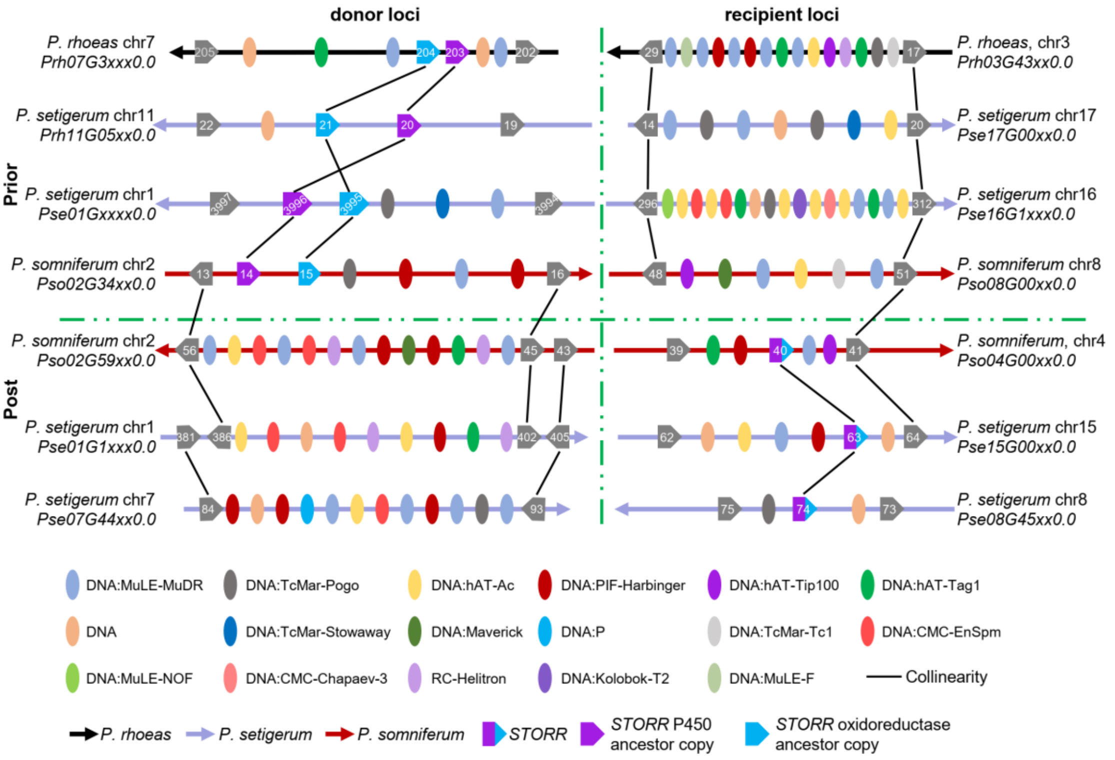

Supplementary Fig. 26. A map of DNA transposable elements in the vicinity of *STORR* donor and recipient loci in three *Papaver* species. Source data are provided as a Source Data file.

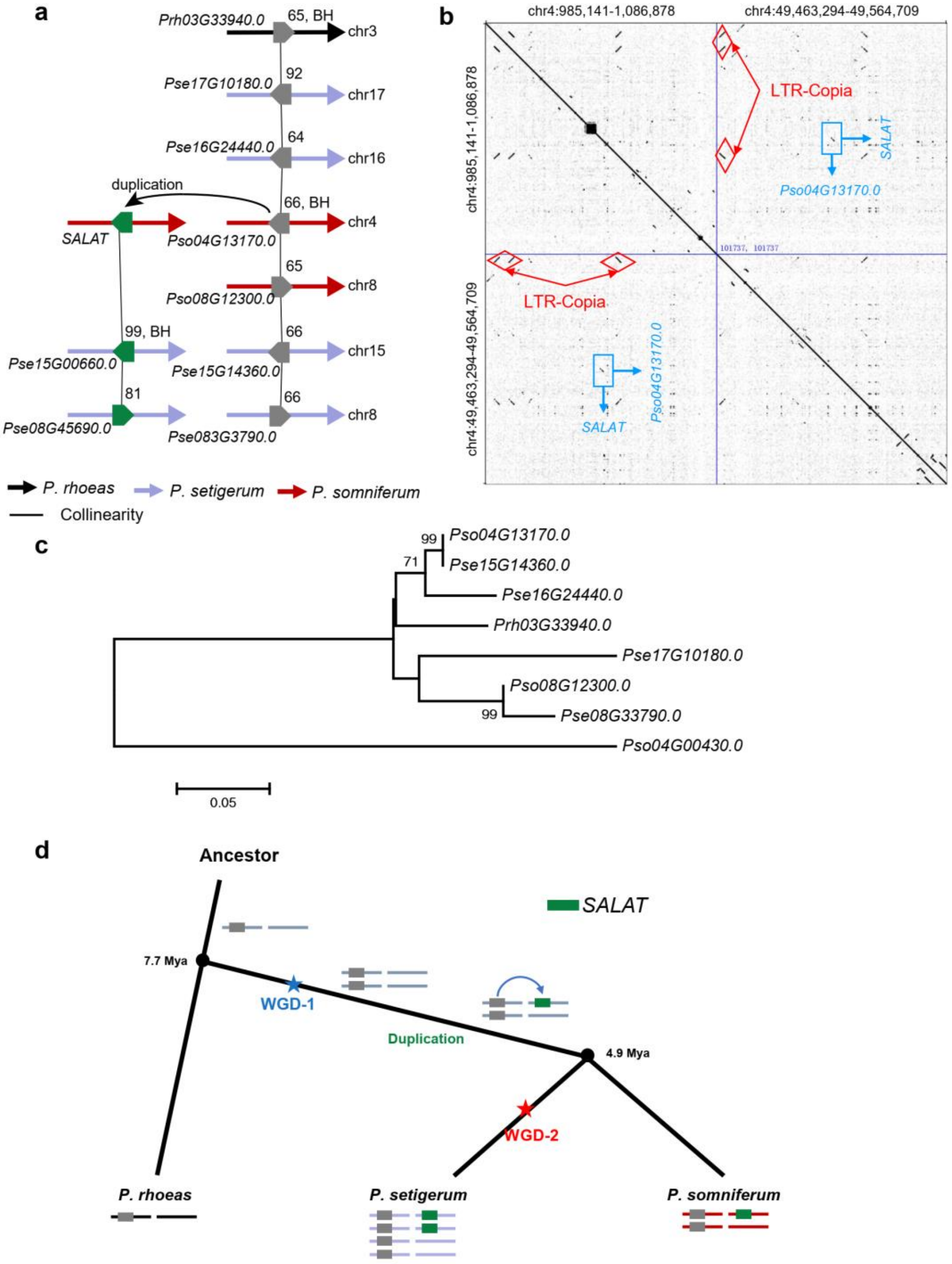

Supplementary Fig. 27. Putative origin of *SALAT* in *Papaver* species. **a.** The synteny and homology of genes related with *SALAT* in three *Papaver* species indicating *SALAT* was duplicated from *Pso04G13170.0* between WGD-2 and the divergence of *P. somniferum* and *P. setigerum*. **b.** The dotplot of *SALAT* and *Pso04G13170.0* sequences. Genes were extended 50kb up- and downstream and gene positions and the annotated *LTR-Copia* positions were labeled on the dotplot. **c**. The gene tree which was constructed based on the protein sequences of gene present in panel a. **d.** The evolutionary model of *SALAT*. BH: best hit in BlastP result. * represents the non-syntenic BH. WGD: whole genome duplication; Mya: million years ago. Source data underlying Supplementary Figure 27a is provided as Source Data file.

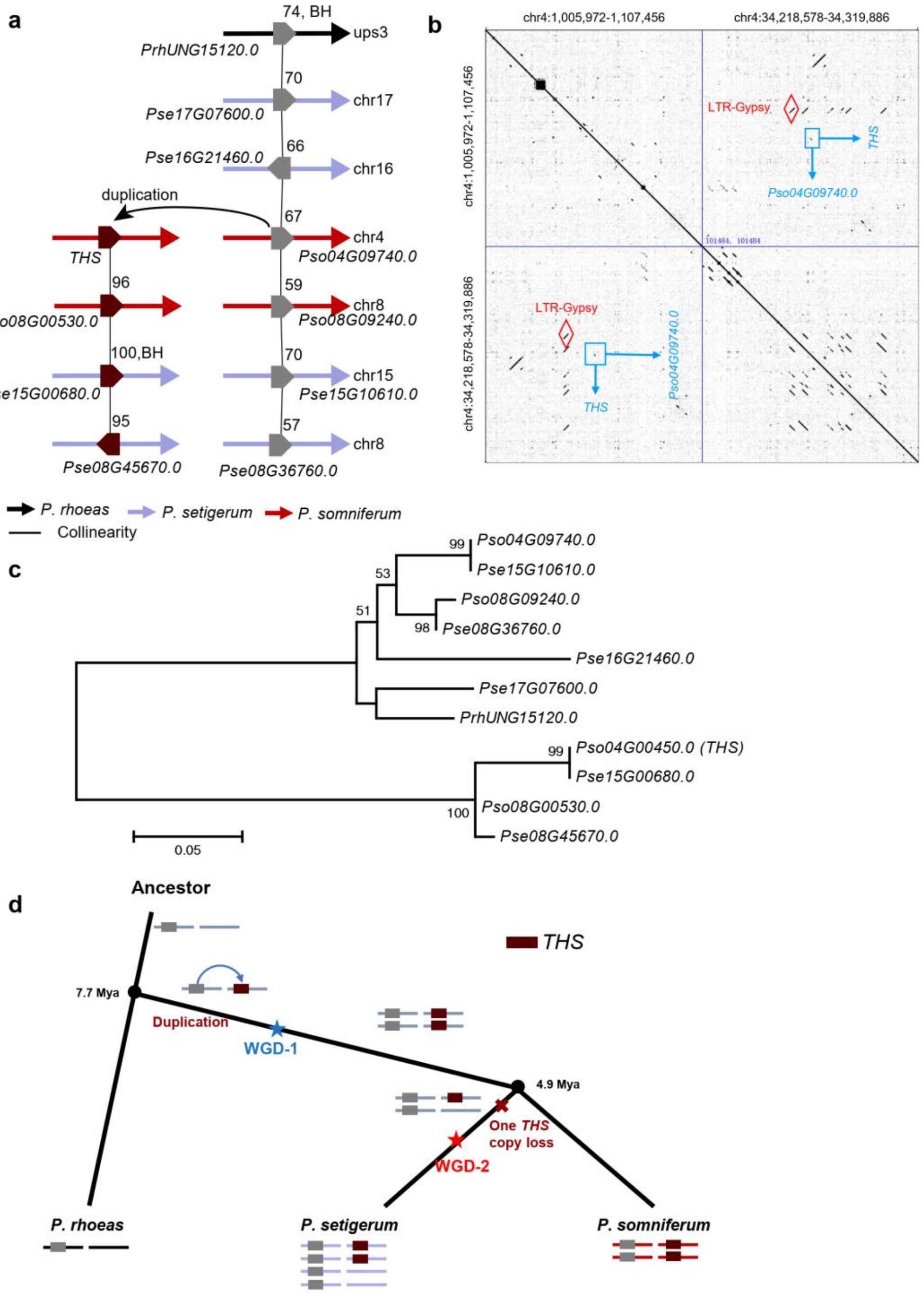

Supplementary Fig. 28. Putative origin of *THS* in *Papaver* species. **a.** The synteny and homology of genes related with *THS* in three *Papaver* species indicating *THS* was duplicated from *Pso04G09740.0* between WGD-1 and the divergence of *P. somniferum* and *P. rhoeas*. **b.** The dotplot of *THS* and *Pso04G09740.0* sequences. Genes were extended 50kb up- and downstream and gene positions and the annotated *LTR-Gypsy* positions were labeled on the dotplot. **c**. The gene tree which was constructed based on the protein sequences of gene present in panel a. **d.** The evolutionary model of *THS*. BH: best hit in BlastP result. * represents the non-syntenic BH. WGD: whole genome duplication; Mya: million years ago. Source data underlying Supplementary Figure 28a is provided as Source Data file.

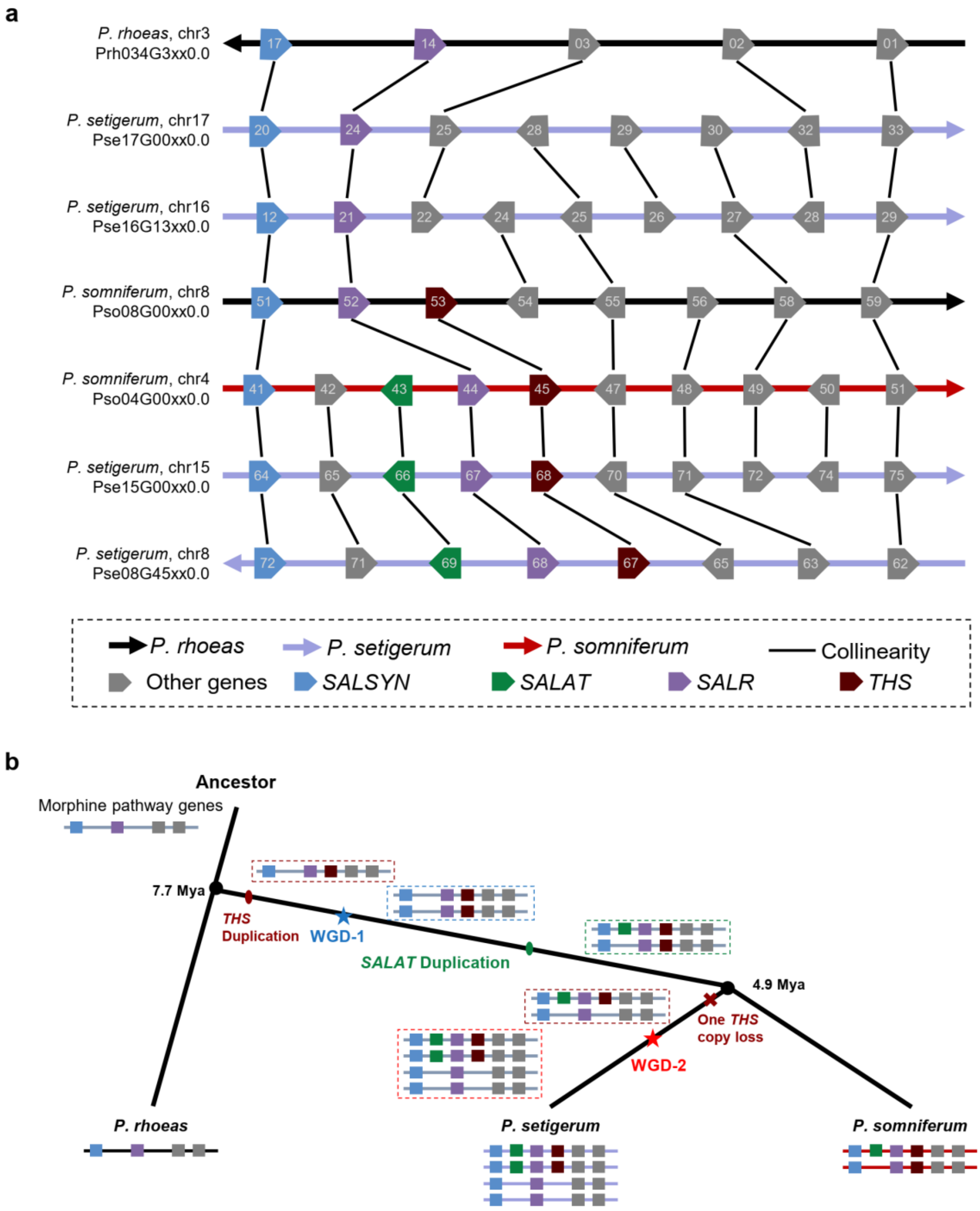

Supplementary Fig. 29. The evolutionary history of morphinan branch genes. **a.** The syntenic relations of the morphinan gene cluster locus harboring *SALSYN*, *SALAT*, *SALR*, and *THS* were illustrated in three *Papaver* species. The direction of arrows indicates the chromosome from 5' to 3', and the last few digits of the gene ID were labeled on the ORFs. **b.** The proposed evolutionary models to indicate the evolutionary history of *SALSYN, SALAT, SALR,* and *THS*. WGD: whole genome duplication; Mya: million years ago. Source data underlying Supplementary Figure 29a is provided as a Source Data file.

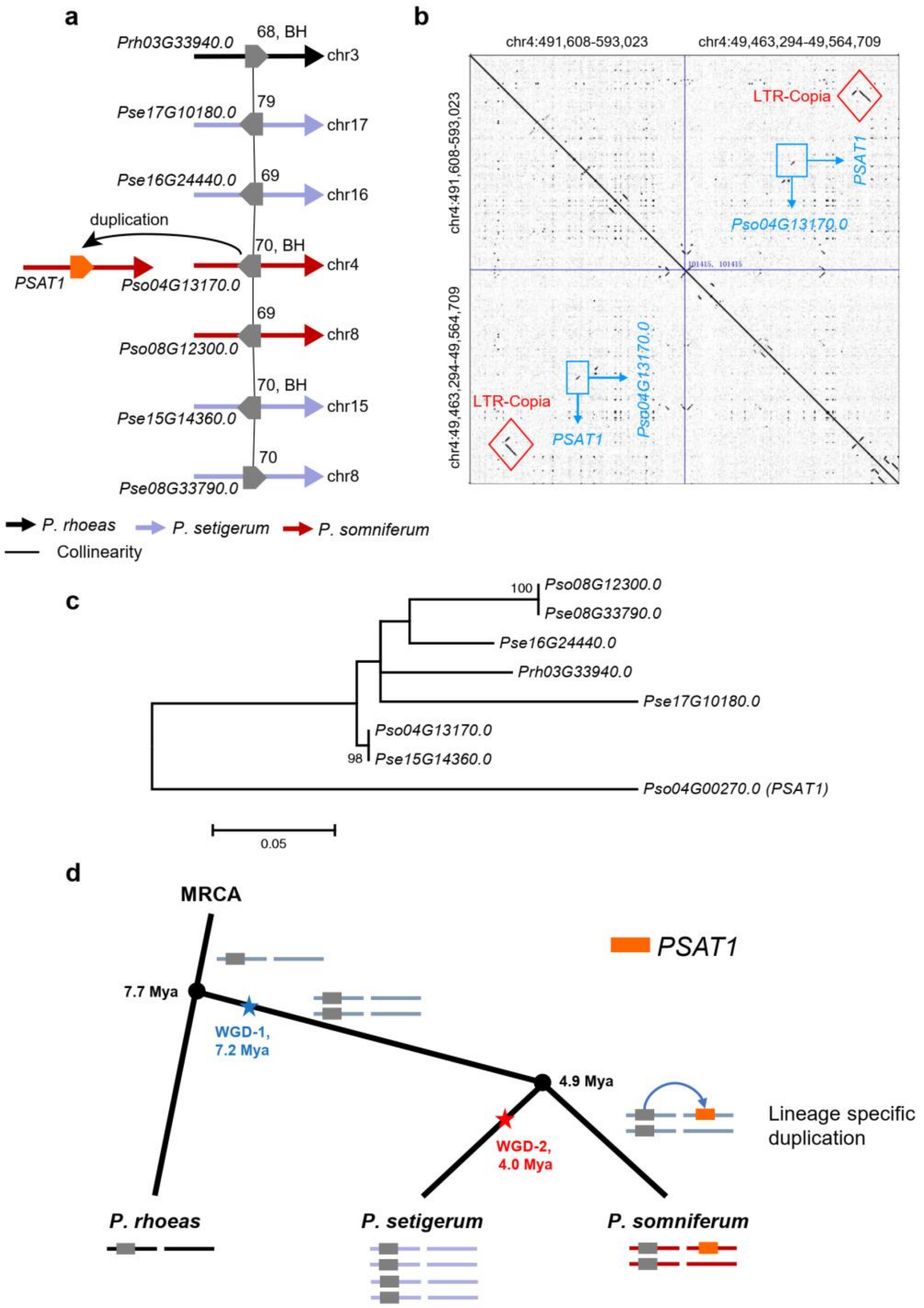

Supplementary Fig. 30. Putative origin of *PSAT1* in three *Papaver* species. **a.** The synteny and homology of genes related with *PSAT1* in three *Papaver* species indicating *PSAT1* was duplicated from *Pso04G13170.0* as a *P. somniferum* specific event. **b.** The dotplot of *PSAT1* and *Pso04G13170.0* sequences. Genes were extended 50kb up- and downstream and gene positions and the annotated *LTR-Copia* positions were labeled on the dotplot. **c**. The gene tree which was constructed based on the protein sequences of gene present in panel a. **d.** The evolutionary model of *PSAT1*. BH: best hit in BlastP result. MRCA: most recent common ancestor; WGD: whole genome duplication; Mya: million years ago. Source data underlying Supplementary Figure 30a is provided as a Source Data file.

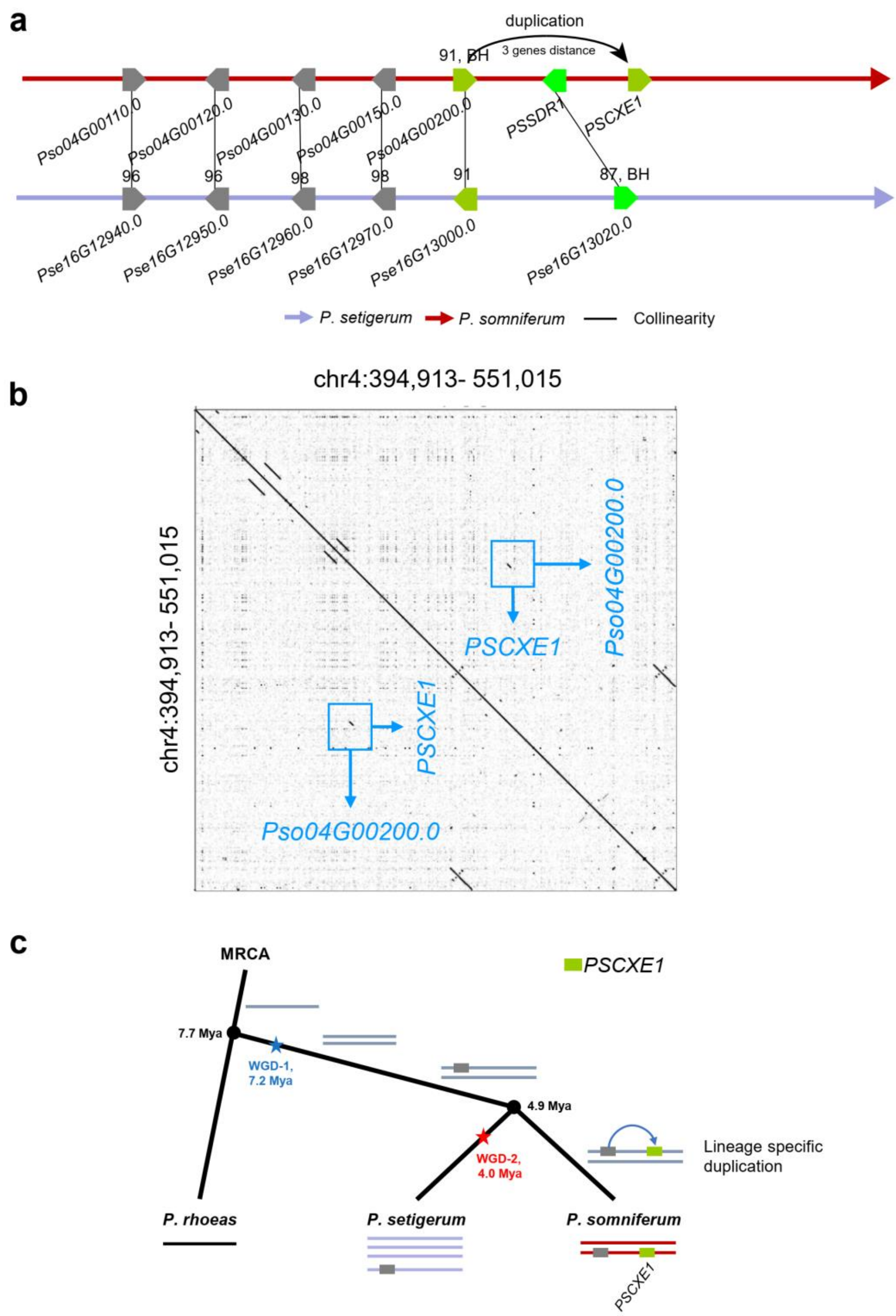

Supplementary Fig. 31. Putative origin of *PSCXE1* in three *Papaver* species. **a.** The synteny and homology of genes related with *PSCXE1* in three *Papaver* species indicating *PSCXE1* was duplicated from *Pso04G00200.0* as a *P. somniferum* specific event. **b.** The dotplot of *PSCXE1* and *Pso04G00200.0* sequences. Genes were extended 50kb up- and downstream and gene positions were labeled on the dotplot. **c**. The evolutionary model of *PSCXE1*. BH: best hit in BlastP result. MRCA: most recent common ancestor; WGD: whole genome duplication; Mya: million years ago. Source data underlying Supplementary Figure 31a is provided as a Source Data file.

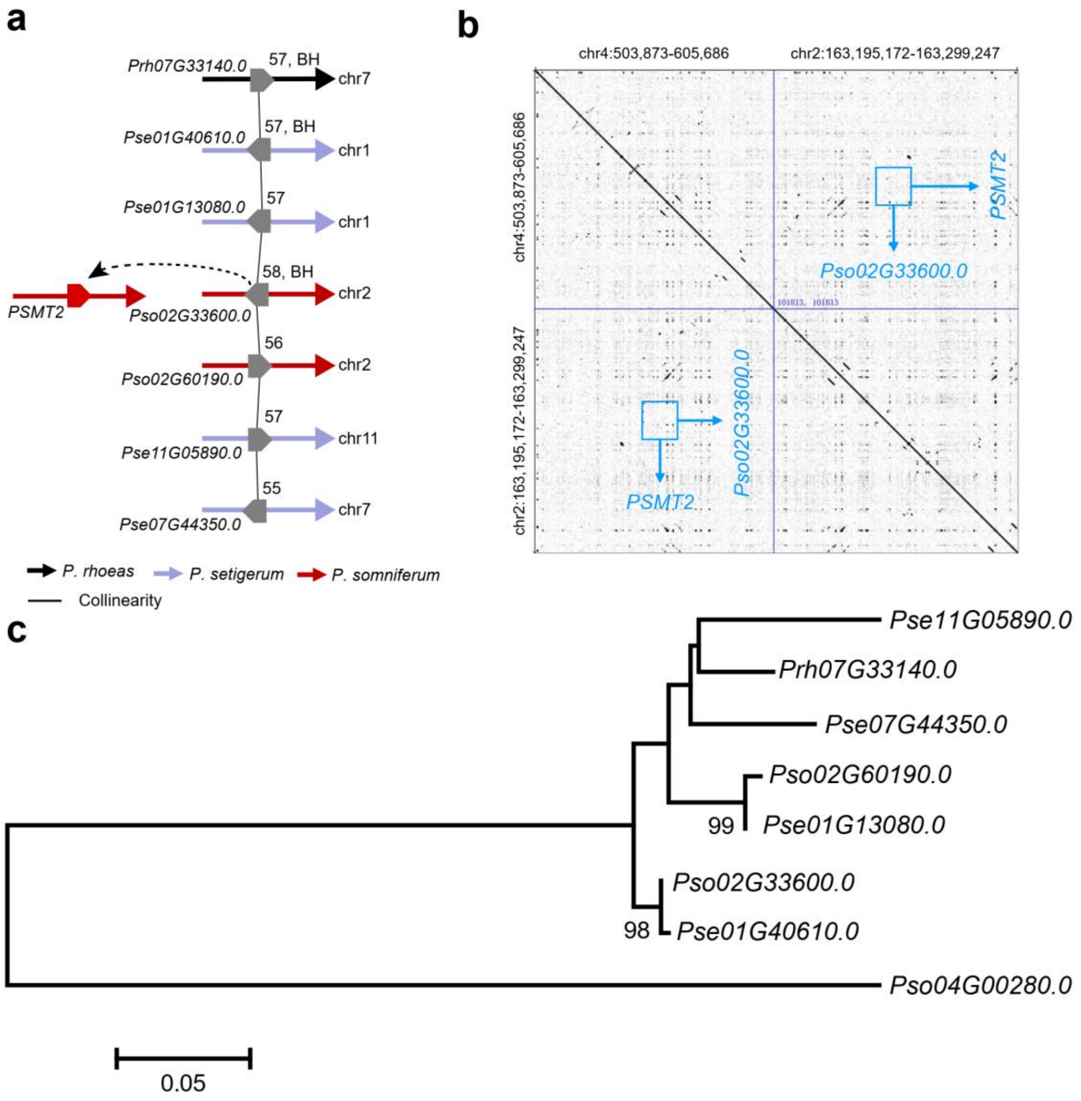

Supplementary Fig. 32. Putative origin of *PSMT2* in three Papaver species. **a.** The synteny homology of genes related with *PSMT2* in three *Papaver* species. *Pso02G33600.0* is the best hit gene of *PSMT2* with protein sequence identity as 58%. However, we did not find any nucleotide alignment between these two gene sequences by BlastN with e-value threshold as 1e-5 suggesting the origin of *PSMT2* was unclear. **b.** The dotplot of *PSMT2* and *Pso04G13170.0* sequences. Genes were extended 50kb up- and downstream and gene positions were labeled on the dotplot. We did not find the alignment of nucleotides in this dotplot. **c**. The gene tree which was constructed based on the protein sequences of gene present in panel a. Source data underlying Supplementary Figure 32a is provided as a Source Data file.

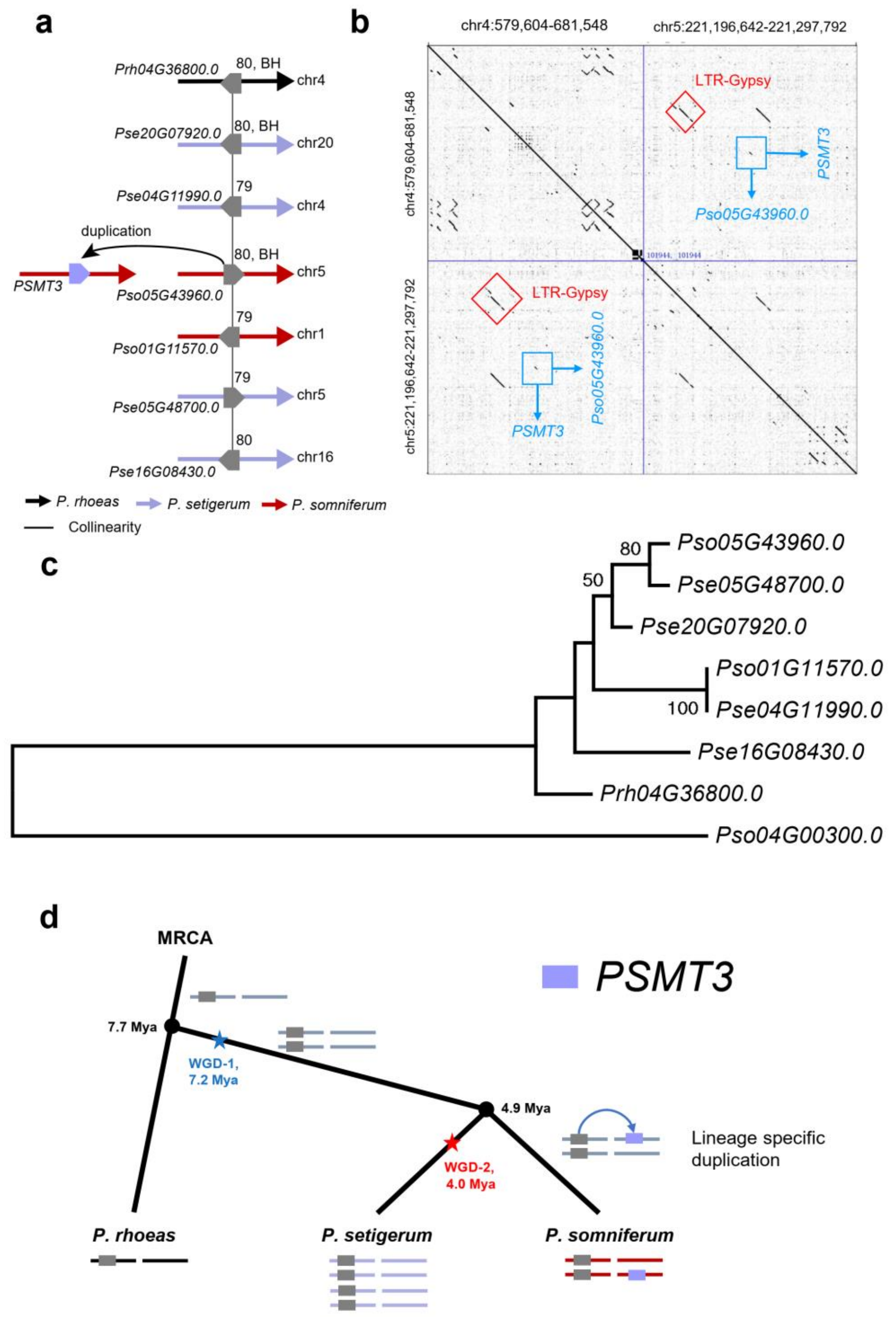

Supplementary Fig. 33. Putative origin of *PSMT3* in three *Papaver* species. **a.** The synteny homology of genes related with *PSMT3* in three *Papaver* species indicating *PSMT3* was duplicated from *Pso05G43960.0* as a *P. somniferum* specific event. **b.** The dotplot of *PSMT3* and *Pso05G43960.0* sequences. Genes were extended 50kb up- and downstream and gene positions and the annotated *LTR-Gypsy* positions were labeled on the dotplot. **c**. The gene tree which was constructed based on the protein sequences of gene present in panel a. **d.** The evolutionary model of *PSMT3*. BH: best hit in BlastP result. MRCA: most recent common ancestor; WGD: whole genome duplication; Mya: million years ago. Source data underlying Supplementary Figure 33a is provided as a Source Data file.

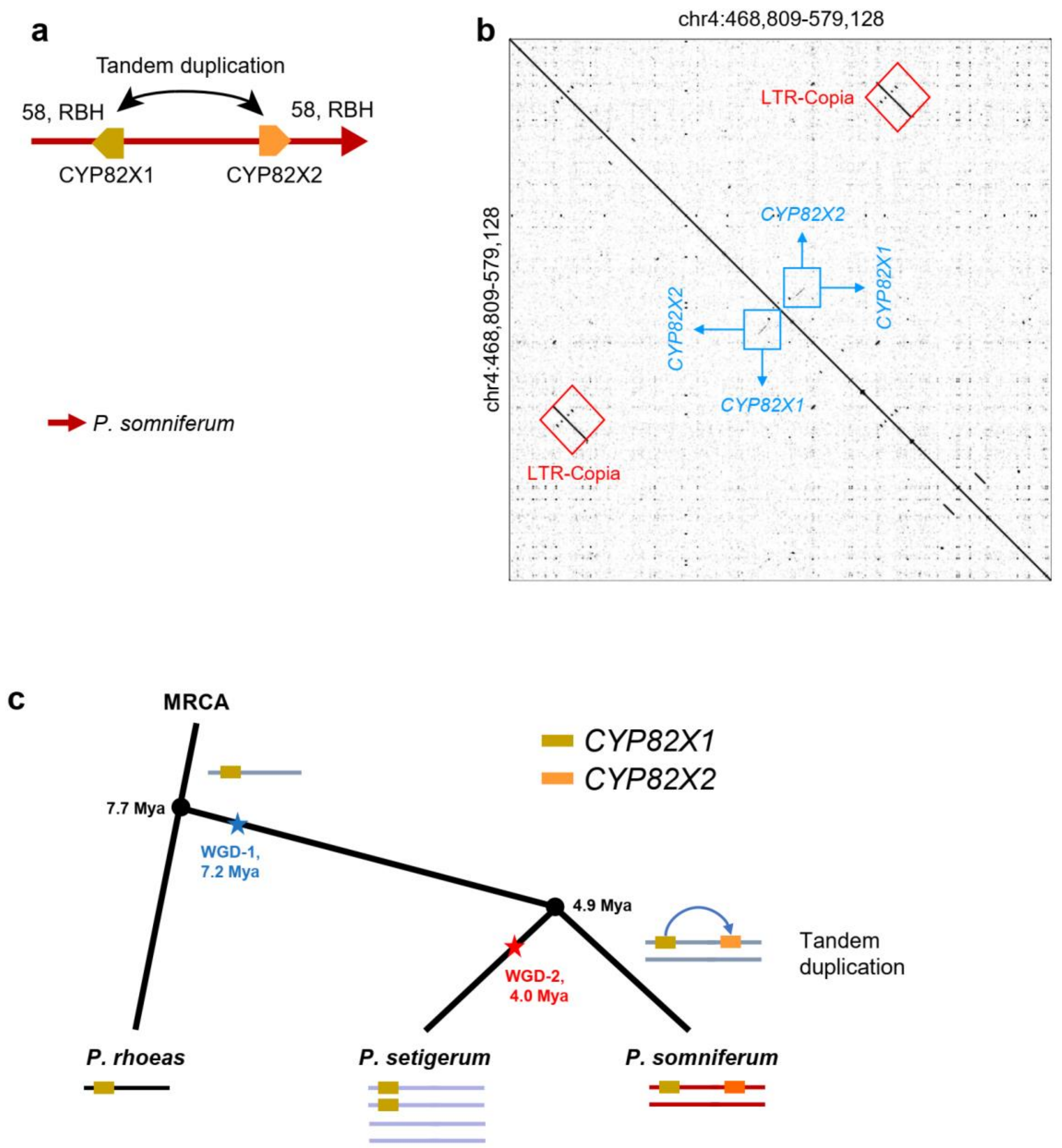

Supplementary Fig. 34. Putative origin of *CYP82X2* in three *Papaver* species. **a.** The homology of *CYP82X2*. *CYP82X1* is the reciprocal best hit (RBH) of *CYP82X2*, and no synteny pairs associated with *CYP82X2* in three species. These suggest *CYP82X2* was tandem duplicated from *CYP82X1* as a *P. somniferum* specific event. **b.** The dotplot of *CYP82X2* and *CYP82X1* sequences. Genes were extended 50kb up- and downstream and gene positions and the annotated *LTR-Copia* positions were labeled on the dotplot. **c.** The evolutionary model of *CYP82X2*. MRCA: most recent common ancestor; WGD: whole genome duplication; Mya: million years ago. Source data underlying Supplementary Figure 34a is provided as a Source Data file.

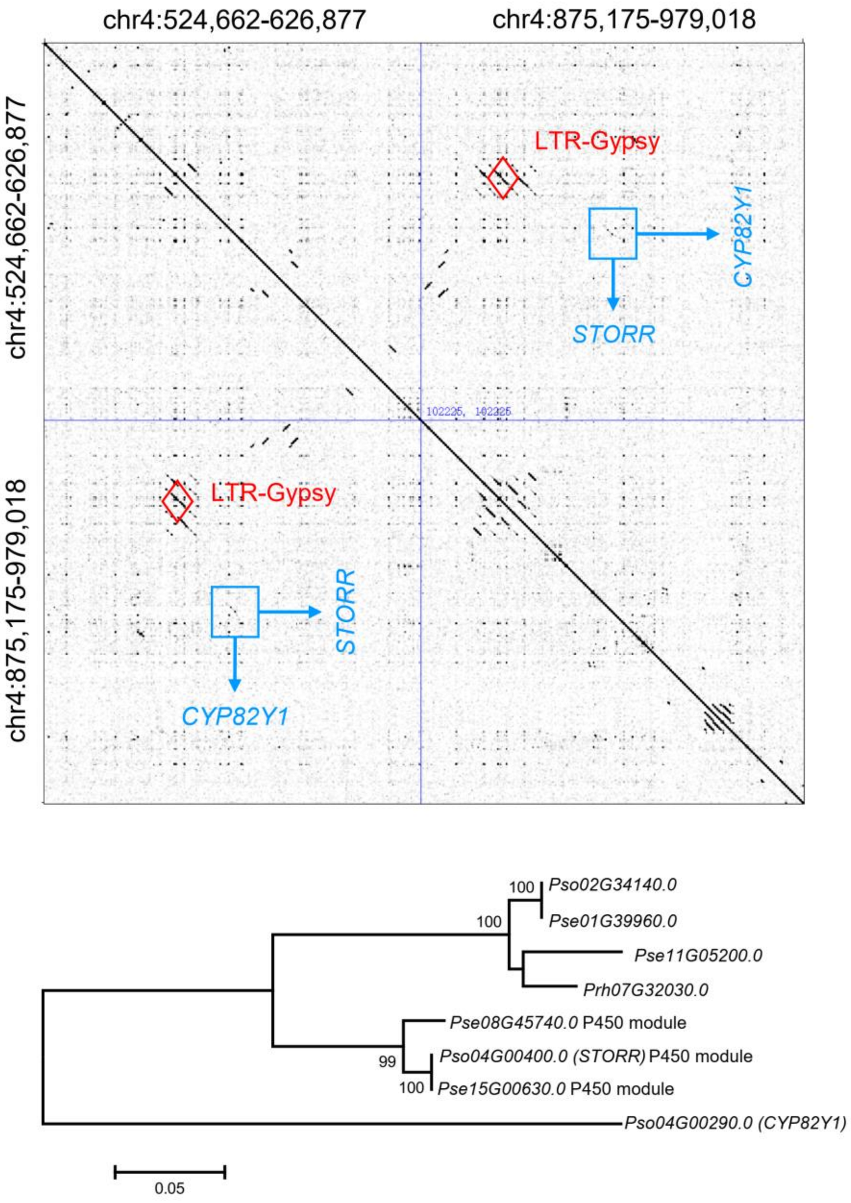

Supplementary Fig. 35. Putative origin of *CYP82Y1* in three *Papaver* species. **a.** The dotplot of *CYP82Y1* and *STORR* sequences. Genes were extended 50kb up- and downstream and gene positions and the annotated *LTR-Gypsy* positions were labeled on the dotplot. *STORR* P450 module is the best hit of *CYP82Y1* with protein sequence identity of 60%. However, we did not find any nucleotide alignment between coding sequence of *CYP82Y1* and STORR P450 module by BlastN with e-value threshold as 1E-5 suggesting the origin of *CYP82Y1* was unclear. **b.** The gene tree which was constructed based on the protein sequences of *CYP82Y1*, *STORR* P450 modules in *P. somniferum* and *P. setigerum*, and the pre-fusion P450 module in three *Papaver* species.

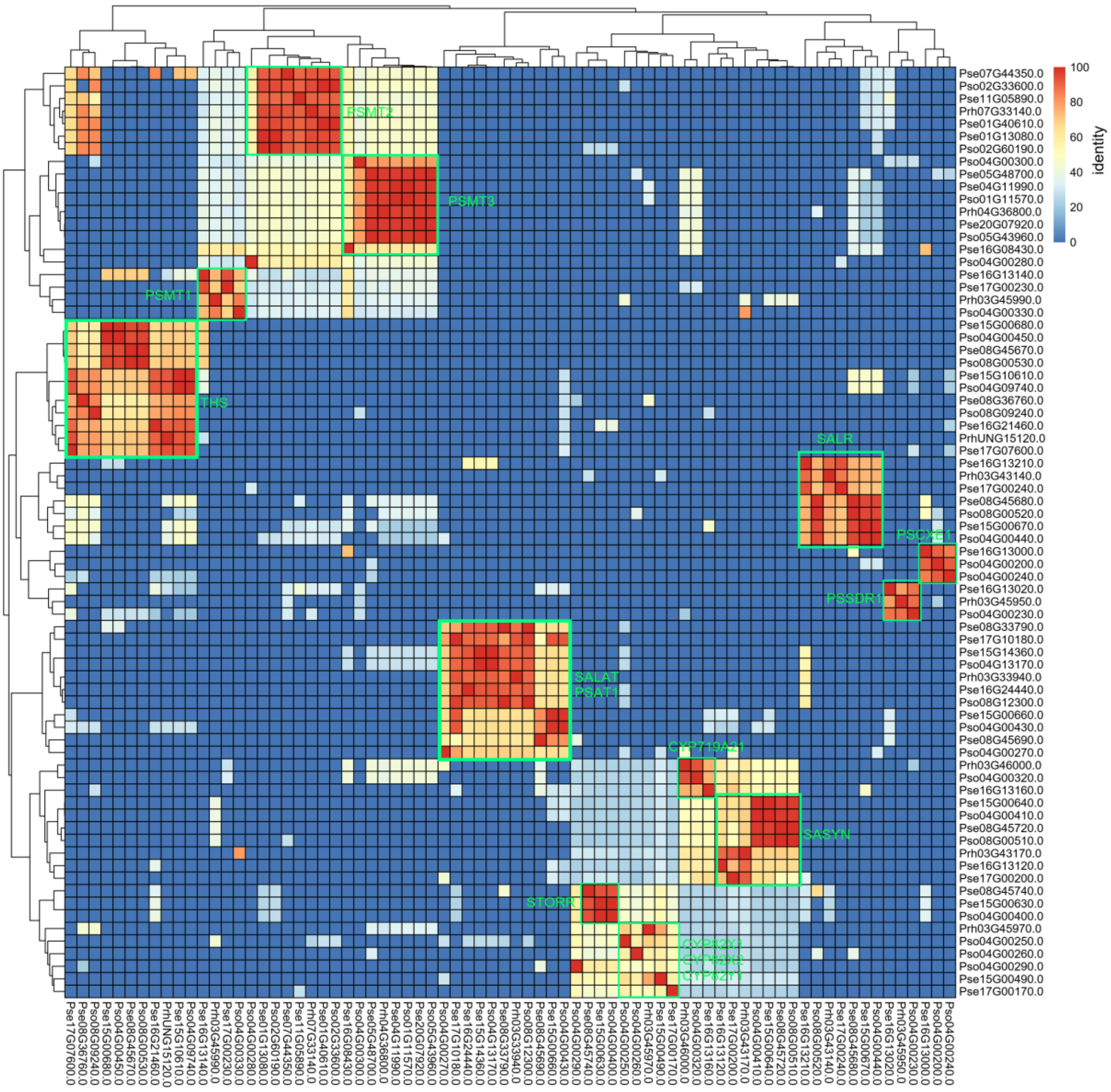

Supplementary Fig. 36. The heatmap of identities between genes related with BIA gene cluster. Source data is provided as a Source Data file.

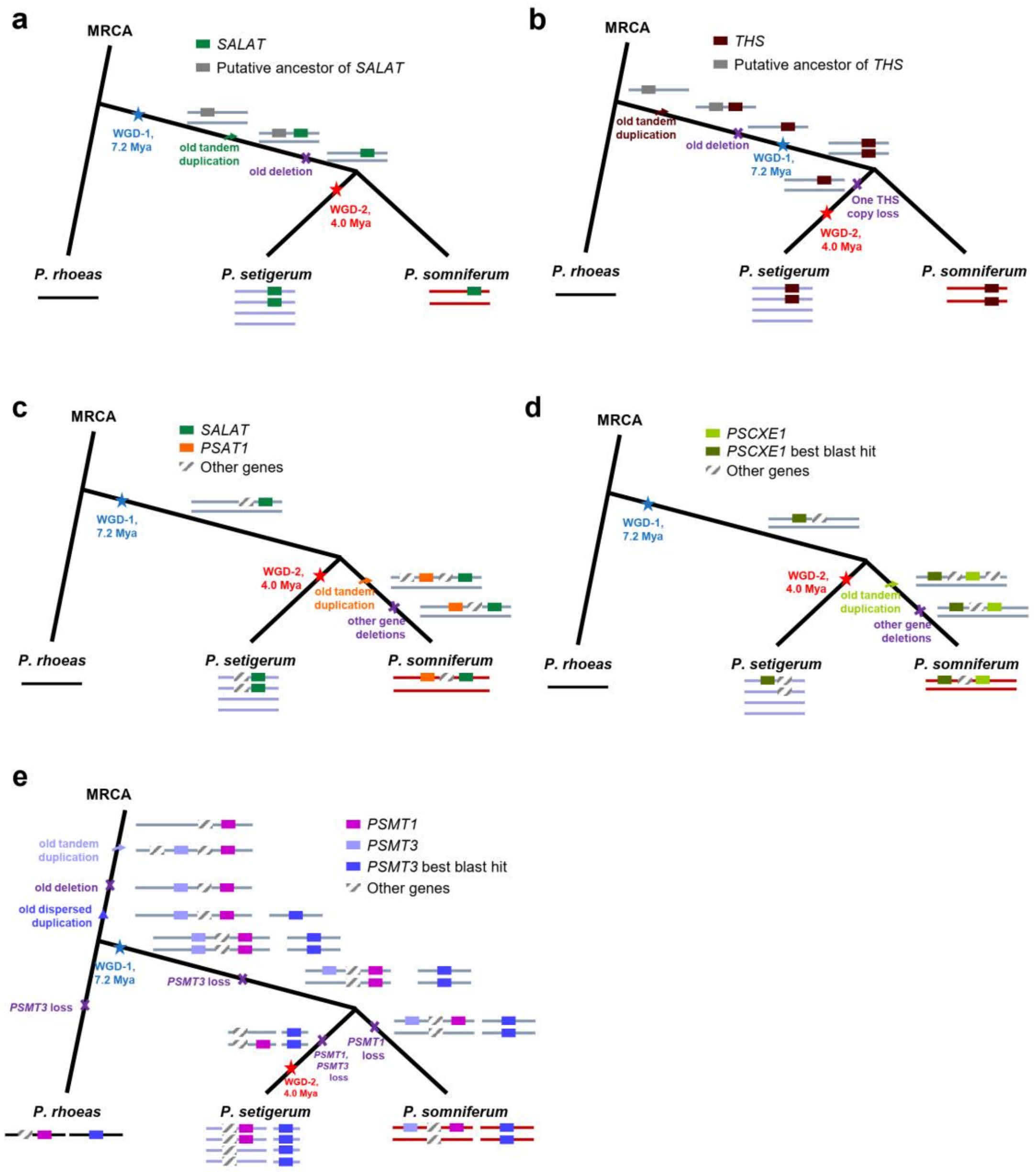

Supplementary Fig. 37. The alternative explanations based on old tandem duplications of the formation of *SALAT* (a), *THS* (b), *PSAT1* (c), *PSCXE1* (d), and *PSMT3* (e). MRCA: most recent common ancestor; WGD: whole genome duplication; Mya: million years ago.

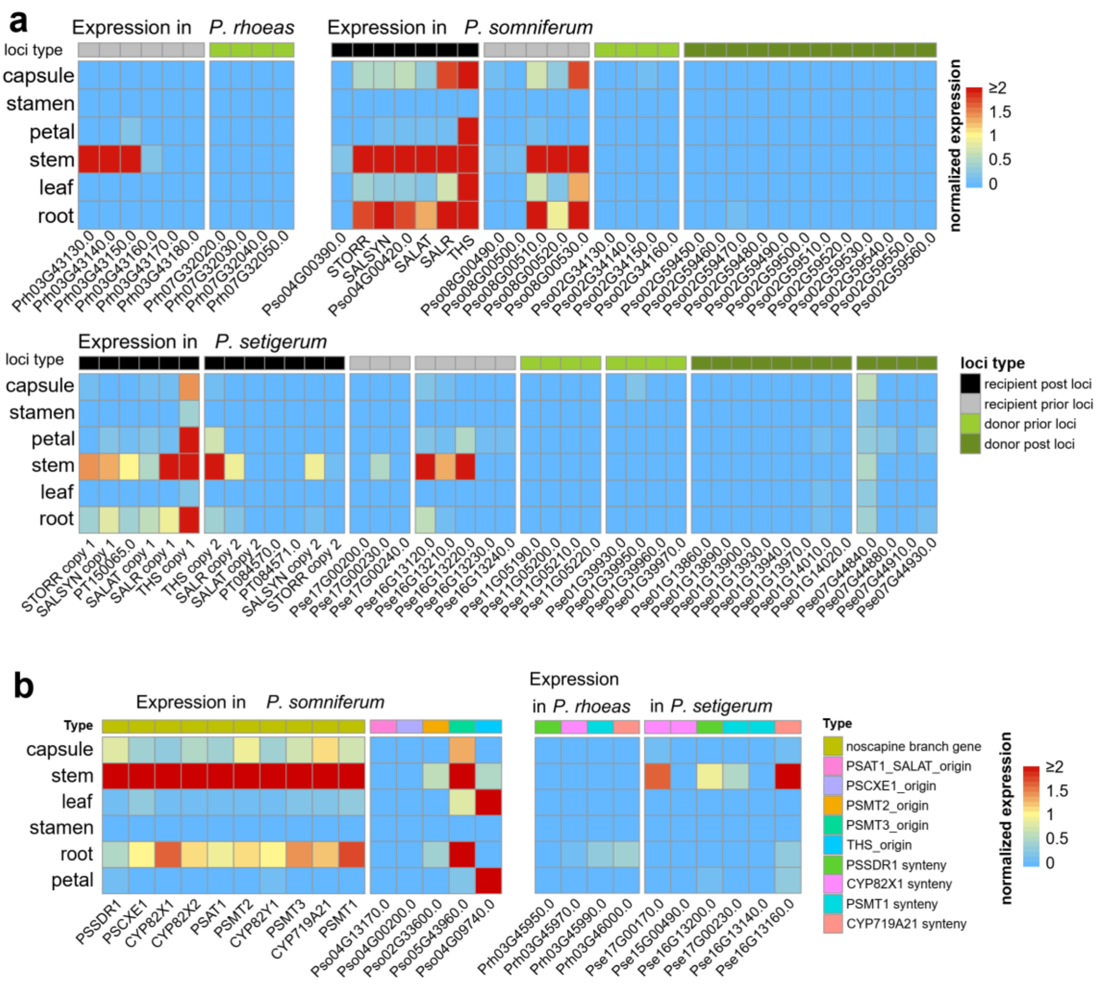

Supplementary Fig. 38. The normalized expression of genes at BIA gene cluster in three species. **a.** Expression of genes at the donor loci and the recipient loci in different tissues of the three species. **b**. Expression of genes related with noscapine branch in different tissues of the three species. Source data is provided as a Source Data file.

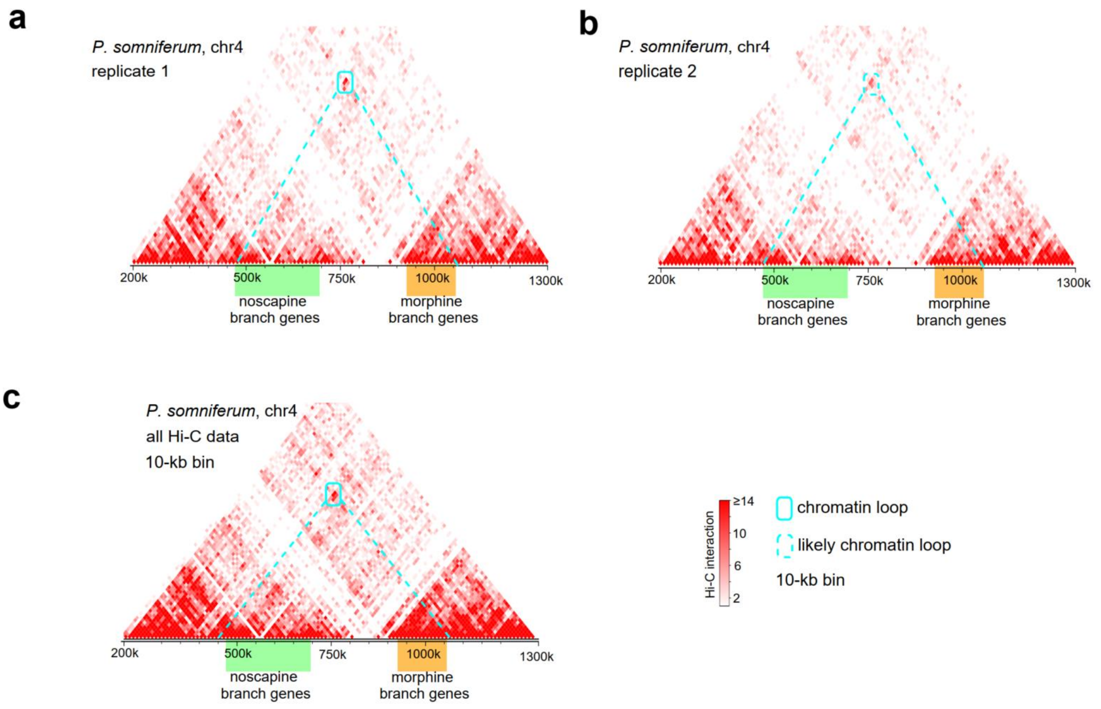

Supplementary Fig. 39. Hi-C interaction heatmap of the region including BIA gene cluster in *P. somniferum*. **a**. The heatmap for replicate 1; **b**. the heatmap for replicate 2; **c**. the heatmap for merged data. Source data is provided as a Source Data file.

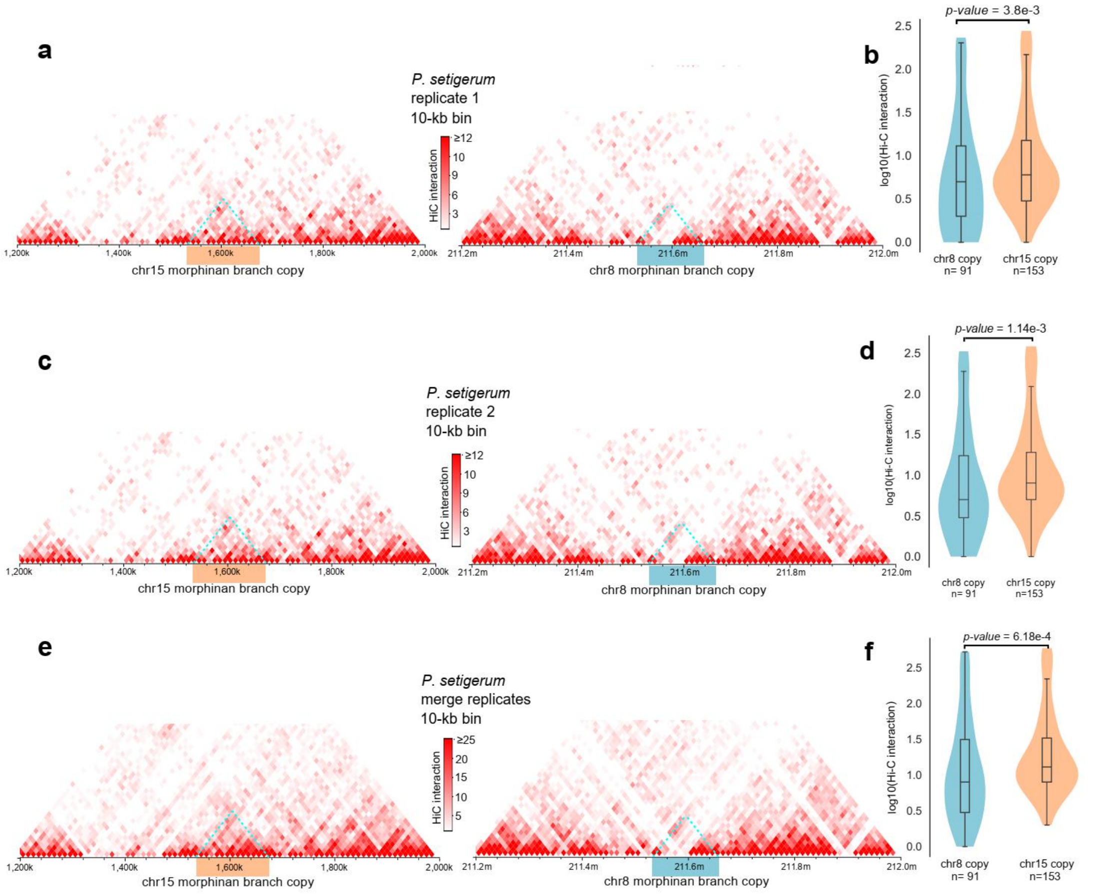

Supplementary Fig. 40. Hi-C interactions of morphinan gene copies in *P. setigerum*. **a.** Hi-C interaction heatmap of regions including two copies of morphinan gene cluster on chr15 (left) and chr8 (right) of *P. setigerum* replicate 1. The morphinan gene cluster regions are marked as orange boxes. **b.** The comparison of the interactions from replicate 1 between two morphinan gene cluster copies in *P. setigerum*. The *p-value* is calculated by two-sided Wilcoxon rank-sum test. **c.** Hi-C interaction heatmap of regions including two copies of morphinan gene cluster on chr15 (left) and chr8 (right) of *P. setigerum* replicate 2. The morphinan gene cluster regions are marked as orange boxes. **d.** The comparison of the interactions from replicate 2 between two morphinan gene cluster copies in *P. setigerum*. The *p-value* is calculated by two-sided Wilcoxon rank-sum test. **e.** Hi-C interaction heatmap of regions including two copies of morphinan gene cluster on chr15 (left) and chr8 (right) of *P. setigerum* merged replicate. The morphinan gene cluster regions are marked as orange boxes. **f.** The comparison of the interactions from merged replicate between two morphinan gene cluster copies in *P. setigerum*. The *p-value* is calculated by two-sided Wilcoxon rank-sum test. For the boxplot, the centre line, median; box limits, upper and lower quartiles; whiskers, data range. Source data are provided as a Source Data file.

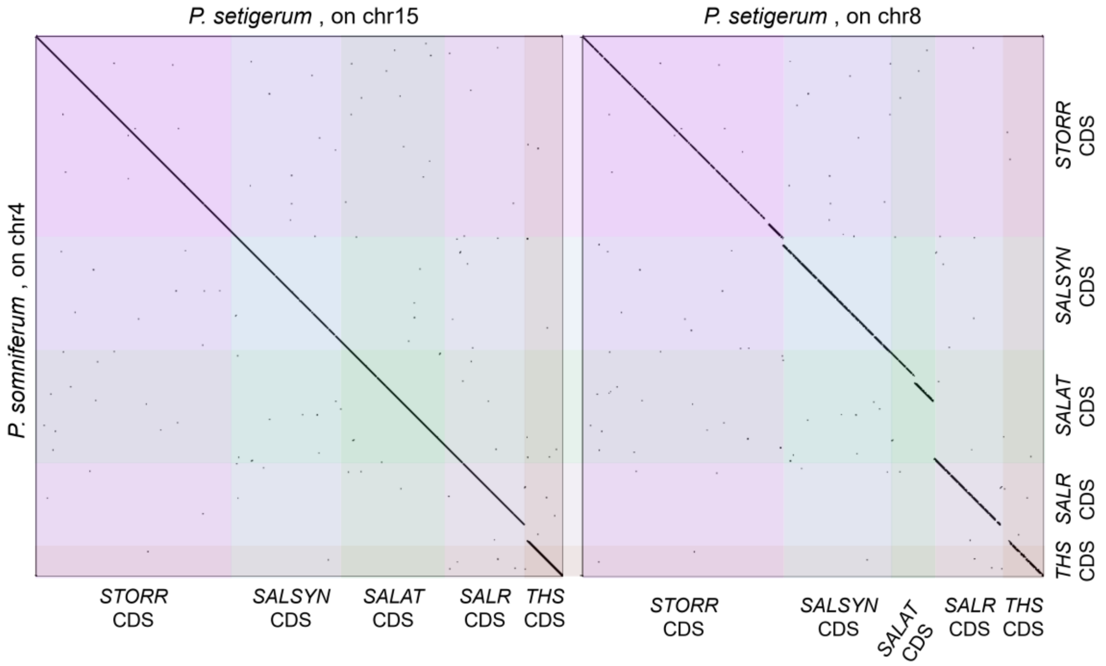

Supplementary Fig. 41. Dotplots of *STORR*, *SALSYN*, *SALAT*, *SALR*, and *THS* CDS sequences between *P. somniferum* (one copy) and *P. setigerum* (two copies). CDS: coding sequence. Source data is provided as a Source Data file.

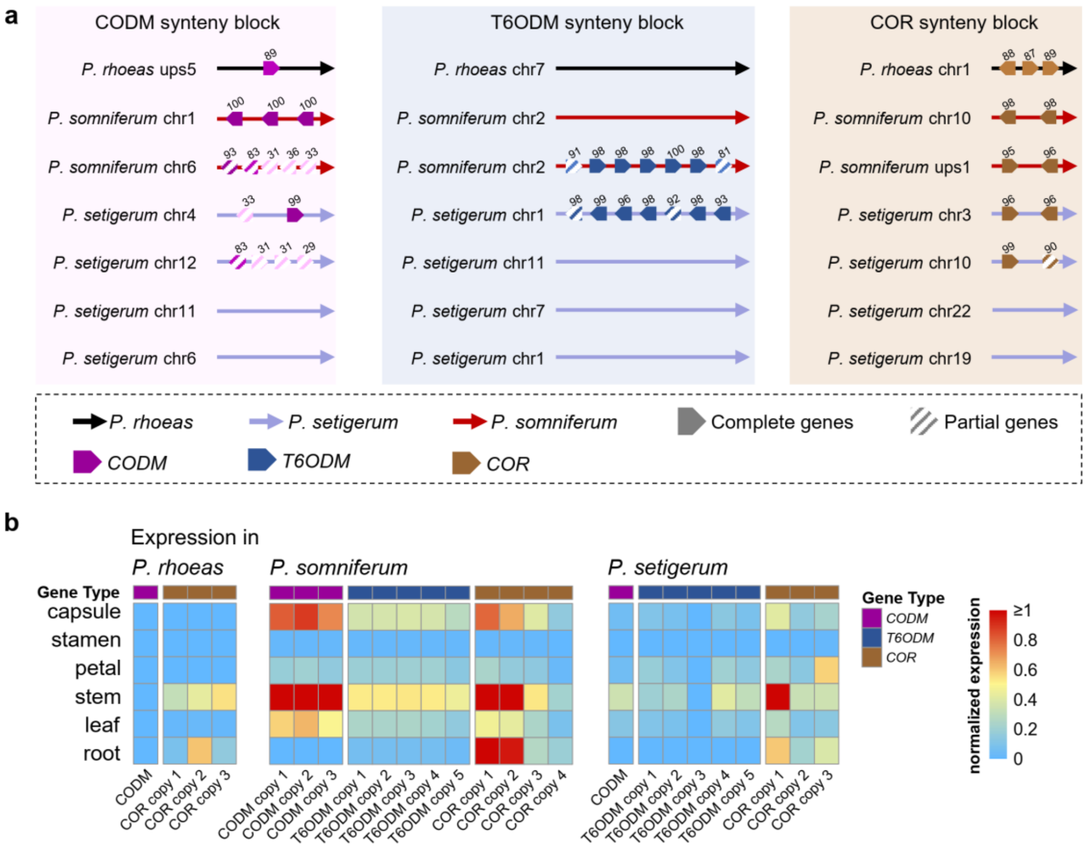

Supplementary Fig. 42. Syntenic block and expression of *CODM*, *T6DOM*, and *COR* in three species. a. The copies of *CODM*, *T6ODM*, and *COR* in the corresponding synteny blocks in three species. **b.** The normalized gene expression of each gene copy in three species. ups: unplaced-scaffold. The number indicates the BlastP identity. Source data are provided as a Source Data file.

Supplementary references

1 Guo, L. *et al.* The opium poppy genome and morphinan production. *Science* **362**, 343-347 (2018).

2 Choe, S. *et al.* Species identification of *Papaver* by metabolite profiling. *Forensic Sci Int* **211**, 51-60 (2011).

3 Hrishi, N. J. Cytogenetical studies on *Papaver somniferum* L. and *Papaver setigerum* DC. and their hybrid. *Genetica* **31**, 1-130 (1960).

4 Claudia, V., Mădălina, V. & Ion, B.I. The study of mitotic chromosomes at *Papaver rhoeas* L. (2n= 14) species. *Analele tiin ifice ale Universit. Cuza din Ia (serie nou), Sec iunea I, Genetic Biologie Molecular*, 188-190 (2004).

5 Asghari-Zakaria, R., Razmi, S., Madadi, R. & Fathi, M. Karyological study of the medicinal plant *Papaver rhoeas* from northwest of Iran. *African Journal of Biotechnology* **10**, 11173-11177 (2011).

6 Dolezel, J., Greilhuber, J. & Suda, J. Estimation of nuclear DNA content in plants using flow cytometry. *Nat Protoc* **2**, 2233-2244 (2007).

7 Zhou, J., Bruns, M. A. & Tiedje, J. M. DNA recovery from soils of diverse composition. *Appl Environ Microbiol* **62**, 316-322 (1996).

8 Louwers, M. *et al.* Tissue- and expression level-specific chromatin looping at maize b1 epialleles. *Plant Cell* **21**, 832-842 (2009).

9 Belton, J. M. *et al.* Hi-C: A comprehensive technique to capture the conformation of genomes. *Methods* **58**, 268-276 (2012).

10 Lander, E. S. & Waterman, M. S. Genomic mapping by fingerprinting random clones: A mathematical analysis. *Genomics* **2**, 231-239 (1988).

11 Hu, X. *et al.* pIRS: Profile-based Illumina pair-end reads simulator. *Bioinformatics* **28**, 1533-1535 (2012).

12 Wick, R. R. & Holt, K. E. Benchmarking of long-read assemblers for prokaryote whole genome sequencing. *F1000Res* **8**, 2138 (2019).

13 Hu, J., Fan, J., Sun, Z. & Liu, S. NextPolish: A fast and efficient genome polishing tool for long-read assembly. *Bioinformatics* **36**, 2253-2255 (2020).

14 Guan, D. *et al.* Identifying and removing haplotypic duplication in primary genome assemblies. *Bioinformatics* **36**, 2896-2898 (2020).

15 Dudchenko, O. *et al. De novo* assembly of the *Aedes aegypti* genome using Hi-C yields chromosome-length scaffolds. *Science* **356**, 92-95 (2017).

16 Simao, F. A., Waterhouse, R. M., Ioannidis, P., Kriventseva, E. V. & Zdobnov, E. M. BUSCO: Assessing genome assembly and annotation completeness with single-copy orthologs. *Bioinformatics* **31**, 3210-3212 (2015).

17 Li, H. & Durbin, R. Fast and accurate short read alignment with Burrows-Wheeler transform. *Bioinformatics* **25**, 1754-1760 (2009).

18 Li, H. *et al.* The Sequence Alignment/Map format and SAMtools. *Bioinformatics* **25**, 2078-2079 (2009).

19 McKenna, A. *et al.* The Genome Analysis Toolkit: A MapReduce framework for analyzing next-generation DNA sequencing data. *Genome Res* **20**, 1297-1303 (2010).

20 Bao, W., Kojima, K. K. & Kohany, O. Repbase update, a database of repetitive elements in eukaryotic genomes. *Mob DNA* **6** (2015).
21 Xu, Z. & Wang, H. LTR_FINDER: An efficient tool for the prediction of full-length LTR retrotransposons. *Nucleic Acids Res* **35**, W265-268 (2007).
22 Ellinghaus, D., Kurtz, S. & Willhoeft, U. LTRharvest, an efficient and flexible software for de novo detection of LTR retrotransposons. *BMC Bioinformatics* **9**, 18 (2008).
23 Ou, S. & Jiang, N. LTR_retriever: A highly accurate and sensitive program for identification of long terminal repeat retrotransposons. *Plant Physiol* **176**, 1410-1422 (2018).
24 Campbell, M. S., Holt, C., Moore, B. & Yandell, M. Genome annotation and curation using MAKER and MAKER-P. *Curr Protoc Bioinformatics* **48**, 4 11 11-39 (2014).
25 Slater, G. S. & Birney, E. Automated generation of heuristics for biological sequence comparison. *BMC Bioinformatics* **6**, 31 (2005).
26 Keller, O., Kollmar, M., Stanke, M. & Waack, S. A novel hybrid gene prediction method employing protein multiple sequence alignments. *Bioinformatics* **27**, 757-763 (2011).
27 Korf, I. Gene finding in novel genomes. *BMC Bioinformatics* **5**, 59 (2004).
28 Lomsadze, A., Ter-Hovhannisyan, V., Chernoff, Y. O. & Borodovsky, M. Gene identification in novel eukaryotic genomes by self-training algorithm. *Nucleic Acids Res* **33**, 6494-6506 (2005).
29 Lamesch, P. *et al.* The Arabidopsis Information Resource (TAIR): Improved gene annotation and new tools. *Nucleic Acids Res* **40**, D1202-1210 (2012).
30 Dohm, J. C. *et al.* The genome of the recently domesticated crop plant sugar beet (*Beta vulgaris*). *Nature* **505**, 546-549 (2014).
31 Jaillon, O. *et al.* The grapevine genome sequence suggests ancestral hexaploidization in major angiosperm phyla. *Nature* **449**, 463-467 (2007).
32 Grabherr, M. G. *et al.* Full-length transcriptome assembly from RNA-Seq data without a reference genome. *Nat Biotechnol* **29**, 644-652 (2011).
33 Jones, P. *et al.* InterProScan 5: Genome-scale protein function classification. *Bioinformatics* **30**, 1236-1240 (2014).
34 Nawrocki, E. P. Annotating functional RNAs in genomes using Infernal. *Methods Mol Biol* **1097**, 163-197 (2014).
35 Nawrocki, E. P. *et al.* Rfam 12.0: Updates to the RNA families database. *Nucleic Acids Res* **43**, D130-137 (2015).
36 Wang, Y. *et al.* MCScanX: A toolkit for detection and evolutionary analysis of gene synteny and collinearity. *Nucleic Acids Res* **40**, e49 (2012).
37 Wang, D., Zhang, Y., Zhang, Z., Zhu, J. & Yu, J. KaKs_Calculator 2.0: a toolkit incorporating gamma-series methods and sliding window strategies. *Genomics Proteomics Bioinformatics* **8**, 77-80 (2010).
38 Murat, F., Armero, A., Pont, C., Klopp, C. & Salse, J. Reconstructing the genome of the most recent common ancestor of flowering plants. *Nat Genet* **49**, 490-496 (2017).

39 International Rice Genome Sequencing, P. The map-based sequence of the rice genome. *Nature* **436**, 793-800 (2005).

40 Filiault, D. L. *et al.* The *Aquilegia* genome provides insight into adaptive radiation and reveals an extraordinarily polymorphic chromosome with a unique history. *eLife* **7**, e36426 (2018).

41 Liu, X. *et al.* The genome of medicinal plant *Macleaya cordata* provides new insights into benzylisoquinoline alkaloids metabolism. *Mol Plant* **10**, 975-989 (2017).

42 Cheng, C. Y. *et al.* Araport11: A complete reannotation of the *Arabidopsis thaliana* reference genome. *Plant J* **89**, 789-804 (2017).

43 Emms, D. M. & Kelly, S. OrthoFinder: Phylogenetic orthology inference for comparative genomics. *Genome Biol* **20**, 238 (2019).

44 Katoh, K. & Standley, D. M. MAFFT multiple sequence alignment software version 7: improvements in performance and usability. *Mol Biol Evol* **30**, 772-780 (2013).

45 Castresana, J. Selection of conserved blocks from multiple alignments for their use in phylogenetic analysis. *Mol Biol Evol* **17**, 540-552 (2000).

46 Stamatakis, A. RAxML-VI-HPC: Maximum likelihood-based phylogenetic analyses with thousands of taxa and mixed models. *Bioinformatics* **22**, 2688-2690 (2006).

47 Sanderson, M. J. r8s: inferring absolute rates of molecular evolution and divergence times in the absence of a molecular clock. *Bioinformatics* **19**, 301-302 (2003).

48 Kumar, S., Stecher, G., Suleski, M. & Hedges, S. B. TimeTree: A resource for timelines, timetrees, and divergence times. *Mol Biol Evol* **34**, 1812-1819 (2017).

49 Scrucca, L., Fop, M., Murphy, T. B. & Raftery, A. E. mclust 5: Clustering, classification and density estimation using gaussian finite mixture models. *R J* **8**, 289-317 (2016).

50 Han, M. V., Thomas, G. W., Lugo-Martinez, J. & Hahn, M. W. Estimating gene gain and loss rates in the presence of error in genome assembly and annotation using CAFE 3. *Mol Biol Evol* **30**, 1987-1997 (2013).

51 Pathan, M. *et al.* FunRich: An open access standalone functional enrichment and interaction network analysis tool. *Proteomics* **15**, 2597-2601 (2015).

52 Rai, A. *et al.* Chromosome-level genome assembly of *Ophiorrhiza pumila* reveals the evolution of camptothecin biosynthesis. *Nat Commun* **12**, 405 (2021).

53 Pham, S. K. & Pevzner, P. A. DRIMM-Synteny: decomposing genomes into evolutionary conserved segments. *Bioinformatics* **26**, 2509-2516 (2010).

54 Tannier, E., Zheng, C. & Sankoff, D. Multichromosomal median and halving problems under different genomic distances. *BMC Bioinformatics* **10**, 120 (2009).

55 Sankoff, D. & Blanchette, M. in *International Computing and Combinatorics Conference.* 251-263 (Springer).

56 Zheng, C., Zhu, Q., Adam, Z. & Sankoff, D. Guided genome halving: hardness, heuristics and the history of the *Hemiascomycetes*. *Bioinformatics* **24**, i96-104

(2008).
57 Feijao, P. & Meidanis, J. SCJ: a breakpoint-like distance that simplifies several rearrangement problems. *IEEE/ACM Trans Comput Biol Bioinform* **8**, 1318-1329 (2011).
58 Yang, X. *et al.* Three chromosome-scale *Papaver* genomes reveal punctuated patchwork evolution of the morphinan and noscapine biosynthesis pathway. *Zenodo*, doi:https://doi.org/10.5281/zenodo.5528515 (2021).
59 Aganezov, S. & Raphael, B. J. Reconstruction of clone- and haplotype-specific cancer genome karyotypes from bulk tumor samples. *Genome Res* **30**, 1274-1290 (2020).
60 Chen, C. *et al.* TBtools: An integrative toolkit developed for interactive analyses of big biological data. *Mol Plant* **13**, 1194-1202 (2020).
61 Kumar, S., Stecher, G. & Tamura, K. MEGA7: Molecular Evolutionary Genetics Analysis version 7.0 for bigger datasets. *Mol Biol Evol* **33**, 1870-1874 (2016).
62 Jones, D. T., Taylor, W. R. & Thornton, J. M. The rapid generation of mutation data matrices from protein sequences. *Comput Appl Biosci* **8**, 275-282 (1992).
63 Bolger, A. M., Lohse, M. & Usadel, B. Trimmomatic: a flexible trimmer for Illumina sequence data. *Bioinformatics* **30**, 2114-2120 (2014).
64 Kim, D., Paggi, J. M., Park, C., Bennett, C. & Salzberg, S. L. Graph-based genome alignment and genotyping with HISAT2 and HISAT-genotype. *Nat Biotechnol* **37**, 907-915 (2019).
65 Pertea, M., Kim, D., Pertea, G. M., Leek, J. T. & Salzberg, S. L. Transcript-level expression analysis of RNA-seq experiments with HISAT, StringTie and Ballgown. *Nat Protoc* **11**, 1650-1667 (2016).
66 Durand, N. C. *et al.* Juicer provides a one-click system for analyzing loop-resolution Hi-C experiments. *Cell Syst* **3**, 95-98 (2016).
67 Kruse, K., Hug, C. B., Hernandez-Rodriguez, B. & Vaquerizas, J. M. TADtool: visual parameter identification for TAD-calling algorithms. *Bioinformatics* **32**, 3190-3192 (2016).
68 Rao, S. S. *et al.* A 3D map of the human genome at kilobase resolution reveals principles of chromatin looping. *Cell* **159**, 1665-1680 (2014).
69 Robinson, J. T. *et al.* Juicebox.js provides a cloud-based visualization system for Hi-C data. *Cell Syst* **6**, 256-258 e251 (2018).
