# Supplementary material for: Three chromosome-scale Papaver genomes reveal punctuated patchwork evolution of the morphinan and noscapine biosynthesis pathway
Source: Nat Commun. 2021 Oct 15;12:6030. doi: 10.1038/s41467-021-26330-8 (PMC8521590; doi:10.1038/s41467-021-26330-8)
Supplement: Supplementary file 3 — Description of Additional Supplementary Files [file 41467_2021_26330_MOESM3_ESM.pdf]

Description of Additional Supplementary Files

Title: Supplementary Data 1.

Description: Details of the sequencing data of three *Papaver* species.

Title: Supplementary Data 2.

Description: Assembly statistics at different stages.

Title: Supplementary Data 3.

Description: Summary of the peaks in Ks distribution and the time estimation.

Title: Supplementary Data 4.

Description: The detail of genes on BIA biosythesis pathway in *P. somniferum* and *P. setigerum*.

Title: Supplementary Data 5.

Description: The morphinan biosythesis pathway related gene alignments and gene expression in three Papaver genomes based on BlastP.

Title: Supplementary Data 6.

Description: The protein family enrichment of expanded families in *P. setigerum* by FunRich.

Title: Supplementary Data 7.

Description: The protein family enrichment of expanded families in *P. somniferum* by FunRich.

Title: Supplementary Data 8.

Description: The protein family enrichment of expanded families in P. rhoeas by FunRich.

Title: Supplementary Data 9.

Description: The fasta file of promoter sequence of genes in the BIA gene cluster.

Title: Supplementary Data 10.

Description: The motifs predicted by FIMO for BIA gene promoters.
