# Supplementary material for: Three chromosome-scale Papaver genomes reveal punctuated patchwork evolution of the morphinan and noscapine biosynthesis pathway
Source: Nat Commun. 2021 Oct 15;12:6030. doi: 10.1038/s41467-021-26330-8 (PMC8521590; doi:10.1038/s41467-021-26330-8)
Supplement: Supplementary file 14 — Reporting Summary [file 41467_2021_26330_MOESM14_ESM.pdf]

nature portfolio

Corresponding author(s): Kai Ye
Last updated by author(s): Sep 24, 2021

Reporting Summary

Nature Portfolio wishes to improve the reproducibility of the work that we publish. This form provides structure for consistency and transparency in reporting. For further information on Nature Portfolio policies, see our Editorial Policies and the Editorial Policy Checklist.

Statistics

For all statistical analyses, confirm that the following items are present in the figure legend, table legend, main text, or Methods section.

n/a	Confirmed	
☐	☒	The exact sample size (*n*) for each experimental group/condition, given as a discrete number and unit of measurement
☐	☒	A statement on whether measurements were taken from distinct samples or whether the same sample was measured repeatedly
☐	☒	The statistical test(s) used AND whether they are one- or two-sided<br>*Only common tests should be described solely by name; describe more complex techniques in the Methods section.*
☒	☐	A description of all covariates tested
☐	☒	A description of any assumptions or corrections, such as tests of normality and adjustment for multiple comparisons
☐	☒	A full description of the statistical parameters including central tendency (e.g. means) or other basic estimates (e.g. regression coefficient) AND variation (e.g. standard deviation) or associated estimates of uncertainty (e.g. confidence intervals)
☐	☒	For null hypothesis testing, the test statistic (e.g. *F*, *t*, *r*) with confidence intervals, effect sizes, degrees of freedom and *P* value noted<br>*Give P values as exact values whenever suitable.*
☒	☐	For Bayesian analysis, information on the choice of priors and Markov chain Monte Carlo settings
☒	☐	For hierarchical and complex designs, identification of the appropriate level for tests and full reporting of outcomes
☒	☐	Estimates of effect sizes (e.g. Cohen's *d*, Pearson's *r*), indicating how they were calculated

Our web collection on statistics for biologists contains articles on many of the points above.

Software and code

Policy information about availability of computer code

Data collection	Sequencing data were collected from Oxford Nanopore and Illumina sequencing machines. NovoExpress v1.2.4.1602 - flow cytometry; Guppy v3.2.2 - nanopore base calling; FastQC (version 0.11.8) - quality control; No additional software was used to collect data.
Data analysis	Lander Waterman algorithm, pIRS v2.0.2 - Genome size estimation; NextDenovo v2.2, Nextpolish v1.2.0, purge_dups (version 1.0.0), breakhic v1.1, 3d-DNA v180922 - Genome assembly; BWA v0.7.17r1188 - Alignment; BUSCO v3 - Assembly and annotation evaluation; SAMTools v1.9 - read depth calculation; GATK v4.1.8 - SNP and indel calling; RepeatModeler vopen-1.0.8, RepeatMasker vopen-4.0.7 - repeat annotation; LTR_Finder v1.1, LTRHarvest v1.5.9, LTR_retriever v2.8.5 - LTR annotation; MAKER2 v2.31.8, AUGUSTUS (v3.3), SNAP (v2006-07-28), GeneMark_ES (v3.48), Blast (ncbi-blast-2.2.28+), Rfam database (v14.1), Trinity v2.1.1 - annotation; InterProScan v5.25-64.0 - function annotation; INFERNAL v1.1.2 - ncRNA annotation; MCScanX (version n/a), DRIMM-synteny (version n/a) - synteny analysis; KaKs_Calculator v2.0 - ka/ks calculation; Mclust R package (version 5.4.7) - time estimation; OrthoFinder v2.3.4, MAFFT v7, Gblocks v0.91b, RAxML v8.2.12, r8s v.1.8 - Phylogenomic analysis; GUROBI solver v9.0.2 - optimization solver; TBtools v.1.0692 - enrichment analysis; Viterbi algorithm, CAFE v3, FunRich v3.1.3 - gene family analysis, Trimommatic v0.32, Hisat2 v2.2.1, Stringtie v2.1.4 - gene expression analysis; MEGA v7.0 - tree construction; Juicer v1.6, HICCUPS (version n/a), Tadtools (v0.76) - HiC data analysis; Customized codes for data analyses are accessible through GitHub https://github.com/yangxiaofeill/IAG and https://github.com/yangxiaofeill/Papaver-Genomics/tree/main/analysis_scripts.

For manuscripts utilizing custom algorithms or software that are central to the research but not yet described in published literature, software must be made available to editors and reviewers. We strongly encourage code deposition in a community repository (e.g. GitHub). See the Nature Portfolio guidelines for submitting code & software for further information.

Data

Policy information about availability of data

All manuscripts must include a data availability statement. This statement should provide the following information, where applicable:

- Accession codes, unique identifiers, or web links for publicly available datasets
- A description of any restrictions on data availability
- For clinical datasets or third party data, please ensure that the statement adheres to our policy

The Oxford Nanopore, Illumina paired-end, Hi-C, transcriptome sequencing data generated in this study have been deposited in the NCBI Sequence Read Archive (SRA) database under accession code PRJNA720042 [https://www.ncbi.nlm.nih.gov/bioproject/PRJNA720042/] and the National Genomics Data Center under accession code PRJCA004217 [https://ngdc.cncb.ac.cn/bioproject/browse/PRJCA004217]. This study used previously published RNA-seq data under accession number GSE111119 [https://www.ncbi.nlm.nih.gov/geo/query/acc.cgi?acc=GSE111119]. The genome assembly data are available at the Genome Warehouse in National Genomics Data Center under accession number GWHAZPI00000000 [https://ngdc.cncb.ac.cn/gwh/Assembly/17874/show], GWHAZPH00000000 [https://ngdc.cncb.ac.cn/gwh/Assembly/17873/show], and GWHAZPJ00000000 [https://ngdc.cncb.ac.cn/gwh/Assembly/17875/show] for P. rhoeas genome, P. setigerum genome and P. somniferum genome, respectively. The genome annotations of three Papaver genomes are available from GitHub [https://xjtu-omics.github.io/Papaver-Genomics]. The used genomes and annotations of Oryza sativa [http://plants.ensembl.org/Oryza_sativa/Info/Index], Arabidopsis thaliana [http://plants.ensembl.org/Arabidopsis_thaliana/Info/Index], and Vitis vinifera [http://plants.ensembl.org/Vitis_vinifera/Info/Index] are downloaded from EnsemblPlants database. The used genomes and annotations of Aquilegia coerulea are downloaded from Phytozome database [https://phytozome-next.jgi.doe.gov/info/Acoerulea_v3_1]. The used genomes and annotations of Macleaya cordata are downloaded from GenBank database under accession number GCA_002174775.1 [https://www.ncbi.nlm.nih.gov/assembly/GCA_002174775.1]. Data supporting the findings of this work are available within the paper and its Supplementary Information files. A reporting summary for this Article is available as a Supplementary Information file. Source data are provided with this paper.

Field-specific reporting

Please select the one below that is the best fit for your research. If you are not sure, read the appropriate sections before making your selection.

☒ Life sciences ☐ Behavioural & social sciences ☐ Ecological, evolutionary & environmental sciences

For a reference copy of the document with all sections, see nature.com/documents/nr-reporting-summary-flat.pdf

Life sciences study design

All studies must disclose on these points even when the disclosure is negative.

Sample size	One individual plant is sufficient for the genome sequencing project for each species. For P. setigerum Hi-C analysis, we compared the Hi-C contacts between chr8 (n=91) and chr15 (n=153) morphinan branch copies. The n is defined as the bin numbers included in the morphinan brach copy.
Data exclusions	no data exclusions
Replication	The replication is not neccessary to a genome sequencing project, since one individual is engough for genome assembly and analysis. Moreover, we repeat three times for karyotyping of each Papvaer species with the similar results. We also did boostrapping in phylogenetic analyses, where 100 replications were used and all of these replications were successful.
Randomization	Randomization was not relevant in the context of the types of statistical analyses performed, where predominantly genomic features or expression data was being analysed. There were no field or lab based experiment that might require randomization.
Blinding	No need to be blinding. This is a plant genome sequencing project, so blinding is not neccessary.

Reporting for specific materials, systems and methods

We require information from authors about some types of materials, experimental systems and methods used in many studies. Here, indicate whether each material, system or method listed is relevant to your study. If you are not sure if a list item applies to your research, read the appropriate section before selecting a response.

Materials & experimental systems

n/a	Involved in the study
☒	☐ Antibodies
☒	☐ Eukaryotic cell lines
☒	☐ Palaeontology and archaeology
☒	☐ Animals and other organisms
☒	☐ Human research participants
☒	☐ Clinical data
☒	☐ Dual use research of concern

Methods

n/a	Involved in the study
☒	☐ ChIP-seq
☐	☒ Flow cytometry
☒	☐ MRI-based neuroimaging

Flow Cytometry

Plots

Confirm that:

☒ The axis labels state the marker and fluorochrome used (e.g. CD4-FITC).

☒ The axis scales are clearly visible. Include numbers along axes only for bottom left plot of group (a 'group' is an analysis of identical markers).

☒ All plots are contour plots with outliers or pseudocolor plots.

☒ A numerical value for number of cells or percentage (with statistics) is provided.

Methodology

Sample preparation	One to two young fresh leaves (equivalent to 300–500 mg) of P. setigerum, P. rhoeas and P. somniferum (internal reference) were collected from four weeks old seedlings, and placed into a 100 mm Petri dish.
Instrument	NovoCyte machine (ACEA Biosciences, Inc.).
Software	NovoExpress software (Version 1.2.4.1602).
Cell population abundance	We use flow cytometry of nuclei. The flow of at least 10000 nuclei was measured in the sample.
Gating strategy	Regarding the gating strategy, there are two gates. GATE1 selects all particles (cells) of FSC/SSC, and GATE2 FL2A/SSC selects the boundaries according to the measured sample mass excluding adhesive particles (cells) and impurities.

☒ Tick this box to confirm that a figure exemplifying the gating strategy is provided in the Supplementary Information.
